# Calpain-mediated TBP cleavage disturbs neuronal AMOT-YAP1 signalling

**DOI:** 10.1038/s41467-026-79055-x

**Published:** 2026-11-09

**Authors:** Rana Dilara Incebacak Eltemur, Florian Fath, Chrisovalantou Huridou, Max Duong Phu, Till Ivo Stoltenow, Jacob Helm, Priscila Pereira Sena, Nicolas Casadei, Ana Velic, Roshan Priyarangana Perera, Boris Macek, Olaf Riess, Stefan Hauser, Huu Phuc Nguyen, Melanie Philipp, Jonasz Jeremiasz Weber

**Affiliations:** 1 Department of Human Genetics, Medical Faculty, Ruhr University Bochum, Bochum, Germany; 2 Institute of Medical Genetics and Applied Genomics, University of Tübingen, Tübingen, Germany; 3 Department of Neurology, Huntington Center NRW, St. Josef-Hospital Bochum, Ruhr University Bochum, Bochum, Germany; 4 Department of Experimental and Clinical Pharmacology and Pharmacogenomics, Section of Pharmacogenomics, University of Tübingen, Tübingen, Germany; 5 German Center for Neurodegenerative Diseases (DZNE), Tübingen, Germany; 6 Hertie Institute for Clinical Brain Research, University of Tübingen, Tübingen, Germany; 7 NGS Competence Center Tübingen, University of Tübingen, Tübingen, Germany; 8 Proteome Center Tübingen, University of Tübingen, Tübingen, Germany

**Keywords:** Molecular neuroscience, Proteolysis, Transcriptomics, Spinocerebellar ataxia, Developmental neurogenesis

## Abstract

The transcription factor TATA-box-binding protein (TBP) plays a crucial role in neurons, as highlighted by its involvement in spinocerebellar ataxia type 17 (SCA17), where pathological polyglutamine expansions disrupt its function. While the molecular pathomechanisms remain unclear, our previous study showed that calcium-activated calpains cleave TBP in SCA17 cell and animal models. Here, we pinpoint a major calpain cleavage site after residue T106, C-terminal to TBP’s polyglutamine tract. The resulting polyglutamine-containing N-terminal fragments mislocalise to the cytoplasm and exhibit increased aggregation. Both the N-terminal and the nuclear-localised C-terminal fragments contribute to calpain overactivation. SCA17 cell line-based multi-omics analyses revealed that TBP fragments alter pathways linked to neuronal function and neurodegeneration, identifying AMOT as a key player. Further analyses in SCA17 cell lines, patient-derived fibroblasts and induced neurons as well as in rat and zebrafish models confirmed AMOT’s pivotal role in neurons, regulated by TBP and mediated via YAP1-dependent signalling. These findings offer further insights into TBP’s neuronal function and suggest broader implications of AMOT for neurodegenerative and neurodevelopmental disorders.

## Abstract (web summary)

TBP fragments activate calpains and disrupt AMOT-YAP1 signalling, revealing a pathway involved in normal neural development and implicated in spinocerebellar ataxia type 17.

## Introduction

The TATA-box-binding protein (TBP) is an essential subunit of the transcription factor IID (TFIID) complex. Its highly conserved C-terminal core domain recognises TATA-box motifs, facilitating RNA polymerase II recruitment for transcription initiation. TBP also contributes to RNA polymerase I and III activity, underscoring its critical role in eukaryotic gene expression^1–3^. Complete loss of TBP is lethal, causing early embryonic arrest^4^.

TBP is crucial for neurological function, supporting neuronal stem cell renewal and differentiation^5^. Moreover, several TBP-associated factors participate in neuronal differentiation and nervous system development^6^. Mutations in TBP have been associated with neurological disorders such as Alzheimer’s disease (AD), Parkinson’s disease (PD), and schizophrenia^7–9^. Notably, polyglutamine (polyQ) expansion mutations in TBP’s exonic CAG/CAA repeat cause spinocerebellar ataxia type 17 (SCA17), an autosomal dominant neurodegenerative disorder characterised by ataxia, dementia, involuntary movements, and psychiatric symptoms. Normal TBP alleles contain 25-40 repeats, while pathogenic alleles exceed 41 repeats, reaching full penetrance at 50. A hallmark of polyQ disorders is the aggregation propensity of expanded polyQ tracts, forming inclusion bodies^10–12^. Despite its discovery over two decades ago^13^, the molecular pathomechanisms driving neuronal loss in SCA17 remain unclear.

SCA17 serves as a valuable model disease for TBP’s role in neuronal processes. PolyQ expansions impair TBP’s ability to dimerise, bind DNA, interact with transcription factors, and regulate transcription^14–17^. Under the *toxic fragment hypothesis*, proteolytic cleavage of disease-linked proteins is a key event in neurodegeneration^18–20^, as seen in AD, PD, and polyQ disorders such as Huntington’s disease (HD), and spinocerebellar ataxia type 3 (SCA3)^21–27^. Interestingly, an internal deletion in the C-terminal region of TBP caused increased aggregation, disrupted TFIID function, and a worsened neurological phenotype in mice^15^.

While earlier studies failed to link TBP fragmentation to caspase activity^25^, we recently demonstrated that calpains generate C-terminal TBP fragments in SCA17 cell and rodent models and that inhibiting calpains mitigates molecular pathology^28^. Here, we investigated the physiological and pathological consequences of TBP cleavage by calpains, identifying specific cleavage sites and generating TBP fragment constructs. Using a multi-omics approach in cells, combined with analysis of neurons differentiated from an induced pluripotent stem cell (iPSC) line of an SCA17 patient, brain tissue from SCA17 rats, and experiments in zebrafish embryos, we investigated the consequences of TBP fragments and identified a TBP-calpain-AMOT regulatory relationship that contributes to normal neural development.

## Results

### Calpain cleavage of TBP occurs C-terminally of its polyQ tract

**Fig. 1:**
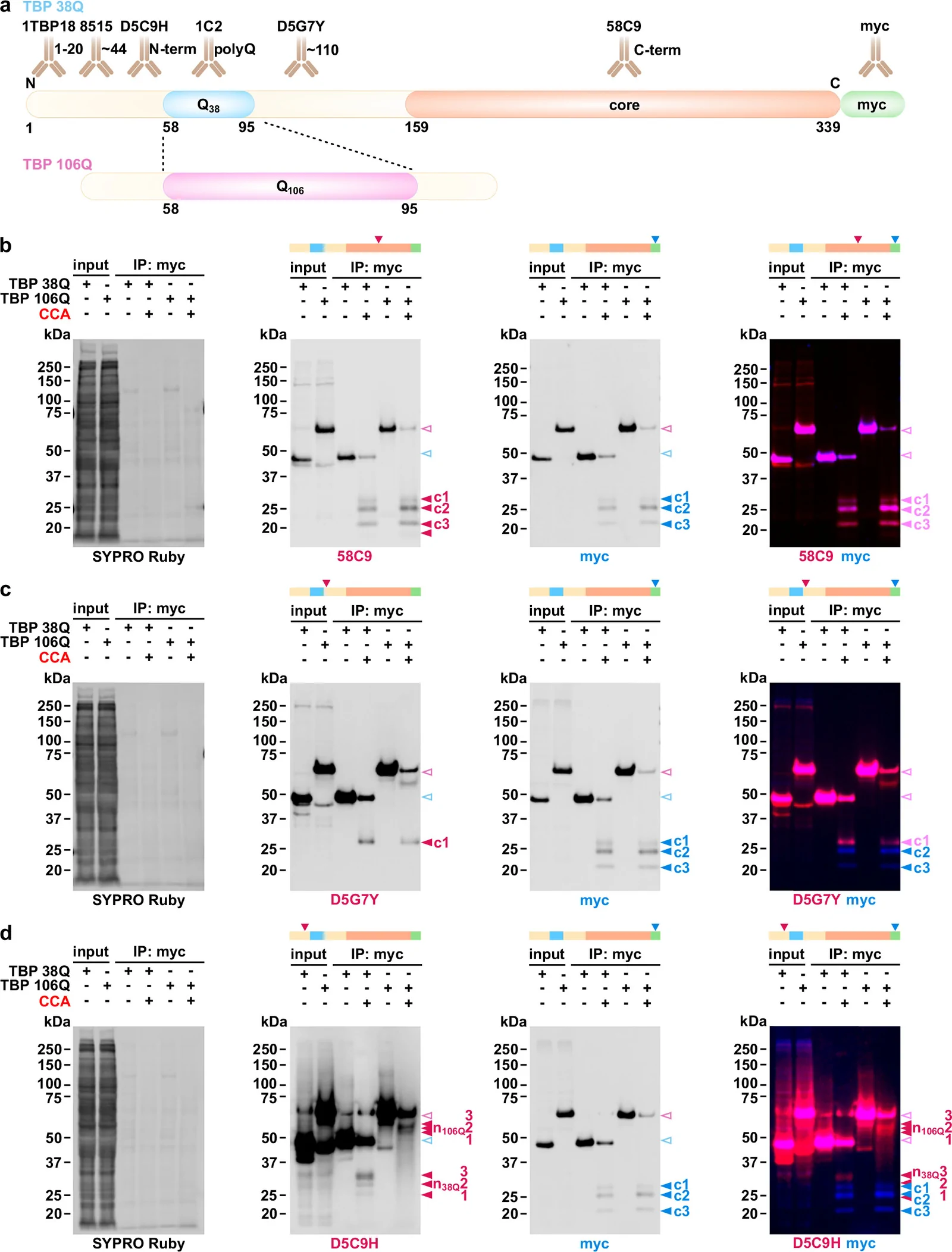
Antibody-based mapping reveals calpain-dependent N-terminal TBP fragments. **a** Schematic overview of myc-tagged non-polyQ-expanded (38Q) and polyQ-expanded (106Q) TBP overexpression constructs, highlighting the polyQ stretch and the C-terminal DNA-binding core domain (core), and binding regions of antibodies used in this study. **b**–**d** Western blot analysis of TBP fragmentation after immunoprecipitation (IP) from 293T cell extracts, followed by a calpain-cleavage assay (CCA) with calpain-1. Membranes were probed with a myc-tag-specific antibody combined with TBP-specific antibodies targeting the C-terminus (58C9, in **b**), an epitope around residue A110 (D5G7Y, in **c**), or the N-terminus (D5C9H, in **d**). SYPRO Ruby staining served as a loading control and for validation of IP purity. Full protein extracts from TBP-transfected 293T cells (input) were loaded along with the corresponding IP samples. Light blue- and pink-rimmed arrowheads mark full-length TBP 38Q and 106Q, respectively. Red and blue arrowheads indicate N-terminal and C-terminal (n1–3; c1–3) TBP fragments, respectively. Light pink arrowheads mark signal overlaps of TBP protein species detected with myc-tag-specific and respective TBP-specific antibodies. The approximate binding sites of the antibodies used are illustrated above the corresponding blots. Source data are provided as a Source Data file.

Previously, we demonstrated that calpains are involved in the proteolytic processing of TBP, resulting in the generation of C-terminal breakdown products^28^. To identify calpain-dependent cleavage sites within TBP, we first extended our in vitro characterisation of the TBP fragmentation. For this, C-terminally myc-tagged non-polyQ-expanded (38Q) or polyQ-expanded (106Q) full-length TBP overexpressed in HEK 293T (293T) cells was immunopurified and cleaved by exogenous calpain-1. To identify N- and C-terminal fragments, samples were analysed by western blotting using a selection of antibodies binding along the myc-TBP protein sequence (Fig. 1a). Immunodetections confirmed successful overexpression and purification of full-length TBP 38Q or 106Q (Fig. 1b–d). Expectedly, the addition of exogenous calpain-1 caused fragmentation of both TBP variants. Immunodetection with antibodies specific for both the myc tag and the C-terminus of TBP (antibody 58C9) revealed three C-terminal fragments (c1, c2, and c3) (Fig. 1b). Detections with antibody D5G7Y, which binds around amino acid residue A110, only detected fragment c1 (Fig. 1c), suggesting that the cleavage site for fragment c1 lies N-terminally to the antibody epitope, while cleavage sites for c2 and c3 are located more C-terminally. Probing with the N-terminus-binding antibody D5C9H, as well as 1TBP18 and 8515, revealed N-terminal, polyQ-containing fragments (n1, n2, and n3) corresponding to the C-terminal TBP breakdown products (Fig. 1d and Supplementary Fig. 1a, b). Immunodetection with the polyQ-specific antibody 1C2 confirmed that these N-terminal TBP fragments indeed contain the polyQ stretch (Supplementary Fig. 1c).

### Calpain-derived C-terminal TBP fragments are mainly nuclear

**Fig. 2:**
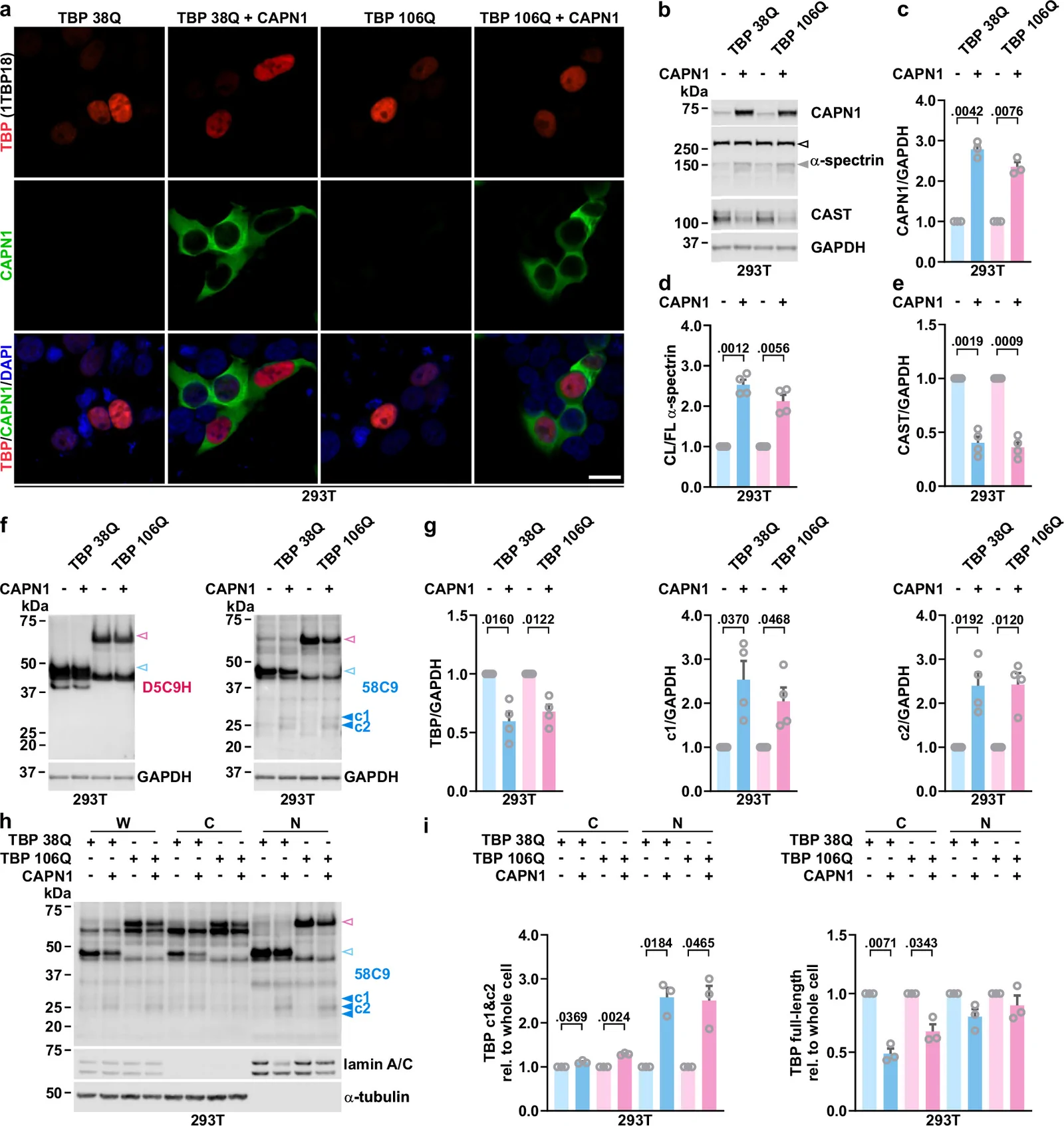
Calpain-dependent C-terminal TBP fragments are enriched in the nucleus of SCA17 cells. **a** Immunofluorescence staining and microscopy of 293T cells expressing TBP 38Q or 106Q with and without calpain-1 (CAPN1) overexpression. TBP was stained with N-terminally binding TBP antibody (1TBP18; red) and CAPN1 with a specific antibody (green). DAPI was used to counterstain cell nuclei (blue). Scale bar: 10 µm. **b** Protein extracts of 293T cells expressing TBP 38Q or 106Q with or without CAPN1 were analysed by western blot. Overexpression of CAPN1 was confirmed using antibodies against CAPN1 and the CAPN1 substrates α-spectrin and calpastatin (CAST). GAPDH served as the loading control. Black-rimmed arrowhead indicates full-length α-spectrin, while the grey arrowhead shows the respective breakdown product. **c**–**e** Densitometric analysis of western blots shows CAPN1 levels (levels normalised to GAPDH; in **c**), α-spectrin cleavage ratio (breakdown product (CL) normalised to full-length (FL) α-spectrin; in **d**) and CAST levels (levels normalised to GAPDH; in **e**). Obtained values were normalised to the respective control (293T cells expressing TBP 38Q) of each measurement. *n* = 3 (**c**) or 4 (**d**, **e**) biological replicates. One-sample *t* test. **f** TBP 38Q and 106Q were detected using N-terminally (D5C9H) and C-terminally (58C9) binding TBP antibodies. GAPDH served as the loading control. Light blue- and pink-rimmed arrowheads mark full-length TBP 38Q and 106Q, respectively. Blue arrowheads indicate C-terminal fragments (c1 and c2). **g** Densitometric analysis of western blot detections with the C-terminal TBP antibody 58C9 shows levels of full-length TBP and TBP fragments c1 and c2. Levels were normalised to GAPDH, and obtained values were normalised to the respective control (293T cells expressing TBP 38Q or 106Q) within each sample set. *n* = 4 biological replicates. One-sample *t* test. **h** 293T cells expressing TBP 38Q or 106Q with and without CAPN1 were subjected to cytoplasmic-nuclear fractionation. Whole cell (W), cytoplasmic fraction (C), and nuclear fraction (N) were analysed by western blotting. Immunodetection was conducted using the C-terminally binding TBP antibody 58C9. Nuclear proteins lamin A and C, and cytoplasmic protein α-tubulin served as fraction-purity and loading control. Light blue- and pink-rimmed arrowheads mark full-length TBP 38Q and 106Q, respectively. Blue arrowheads indicate C-terminal fragments (c1 and c2) (**f**). **i** Densitometric analysis shows levels of full-length TBP and TBP fragments c1 and c2. Levels were normalised to the subcellular fraction-specific loading control, followed by normalisation to the respective control condition within the same fraction (293T cells expressing TBP 38Q or 106Q). *n* = 3 biological replicates. One-sample *t* test). Bars represent means + s.e.m. All tests were two-tailed. *P* values are shown in the graphs. Source data are provided as a Source Data file.

TBP is predominantly localised in the nucleus^29,30^. However, the subcellular distribution of calpain-dependent TBP fragments remained elusive. To examine this further, we induced calpain-dependent TBP fragmentation in cellulo by co-transfecting 293T cells with myc-tagged full-length TBP 38Q or 106Q and FLAG-tagged calpain-1. Immunofluorescence staining and microscopy of TBP with the N-terminally binding antibody 1TBP18 confirmed the predominantly nuclear localisation of TBP, while calpain-1 was detected exclusively in the cytoplasm (Fig. 2a). Analysis of protein extracts from 293T cells co-expressing full-length TBP 38Q or 106Q with calpain-1 via western blotting confirmed the overexpression of calpain-1 and the proteolytic breakdown of its substrates α-spectrin and calpastatin (Fig. 2b–e). Immunodetection of TBP using the N-terminally binding D5C9H and C-terminally binding 58C9 antibodies revealed a reduction in full-length TBP 38Q or 106Q upon overexpression of calpain-1, along with increasing levels of C-terminal TBP fragments c1 and c2, corroborating the fragmentation of TBP by calpains. However, N-terminal TBP fragments were not detectable (Fig. 2f, g). Similar results were observed in 293T cells transfected with the same TBP 38Q or 106Q constructs and treated with ionomycin—an ionophore that depletes intracellular calcium stores and activates calpains^31,32^. Correspondingly, ionomycin treatment led to reduced levels of full-length TBP and elevated levels of fragments c1 and c2 (Supplementary Fig. 2a, b). To determine the subcellular localisation of calpain-dependent TBP fragments c1 and c2, we repeated the co-expression of TBP 38Q or 106Q along with FLAG-tagged calpain-1 in 293T cells and performed cytoplasmic-nuclear fractionation. Western blot analysis of the resulting subcellular fractions using the C-terminal antibody 58C9 showed significant TBP cleavage upon co-expression of calpain-1 and accumulation of fragments c1 and c2 primarily in the nuclear fraction (Fig. 2h, i). Interestingly, cytoplasmic levels of full-length TBP 38Q and 106Q dropped significantly, while full-length TBP in the nuclear fraction remained unchanged (Fig. 2i). To rule out that calpain-mediated TBP cleavage might occur in the nucleus, we investigated the subcellular localisation of all calpains expressed at appreciable levels in 293T cells based on our transcriptome analysis (Supplementary Fig. 2c). Immunofluorescence staining and microscopy showed that all endogenously expressed calpains—including calpain-1, -2, -5, -7, -10 and -15—as well as the calpain small subunit CAPNS1 and calpastatin were localised to the cytoplasm (Supplementary Fig. 2d). Moreover, knockdown of *CAPN1*, but not *CAPN2*, was sufficient to reduce TBP fragment levels, particularly of fragment c1, at baseline (Supplementary Fig. 2e, f), while calpastatin knockdown increased TBP cleavage (Supplementary Fig. 3). These results indicate that TBP fragmentation by calpain occurs in the cytoplasm, followed by the nuclear translocation of TBP fragments c1 and c2.

### Mass spectrometry analysis identifies T106 | S107 in TBP as a main calpain-1 cleavage site

**Fig. 3:**
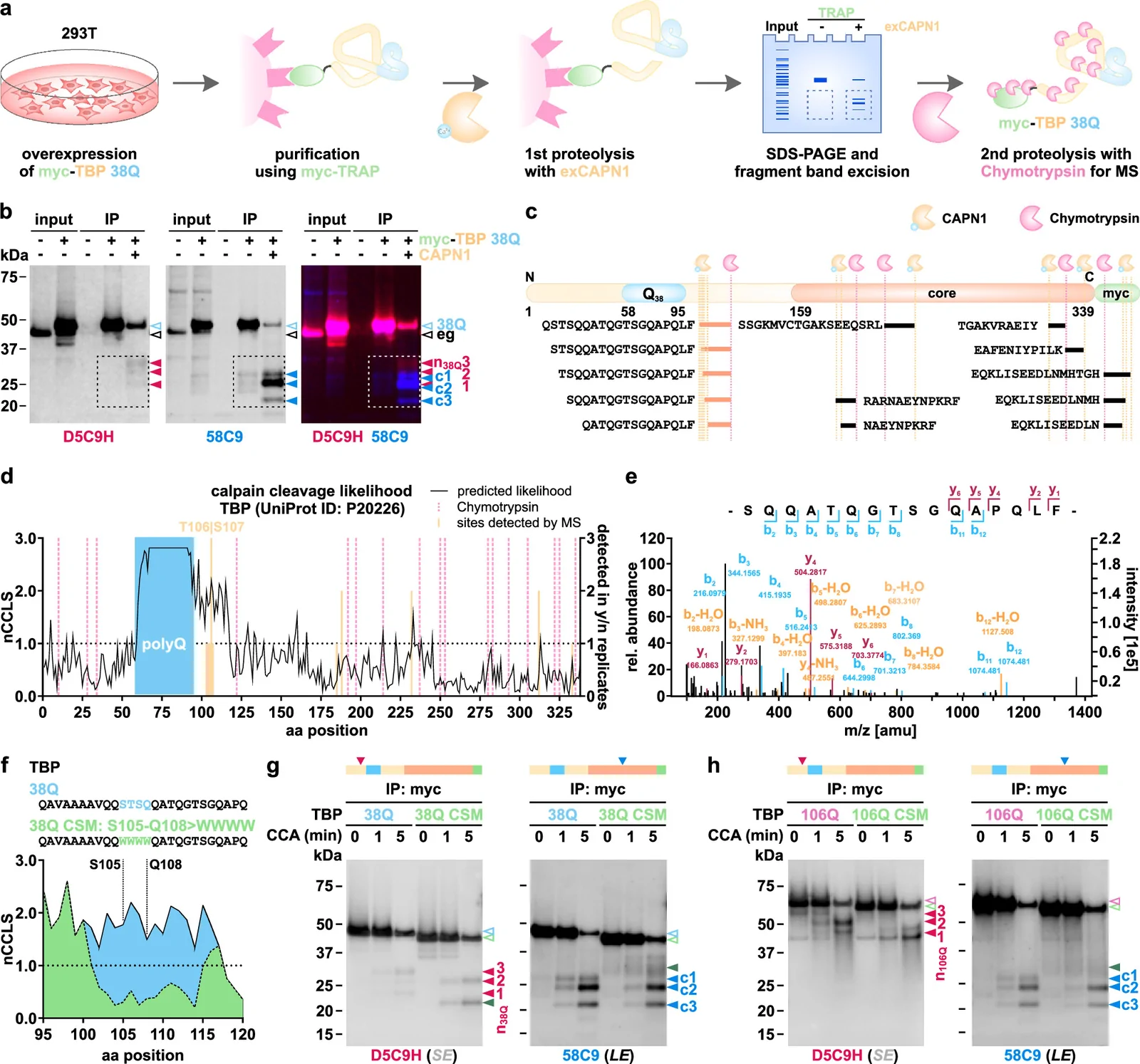
Mass spectrometry analysis identifies T106 | S107 as a calpain-1 cleavage site in TBP. **a** Schematic illustration of the experimental workflow for identifying calpain-1 (CAPN1) cleavage sites in TBP using immunocapture and calpain-cleavage assay (CCA)-based mass spectrometry (MS). exCAPN1, exogenous CAPN1. **b** Western blot detection of immunopurified (IP) and CAPN1-cleaved myc-tagged TBP 38Q using N-terminally binding (D5C9H) and C-terminally binding (58C9) TBP antibodies. The light-blue-rimmed arrowhead indicates full-length TBP 38Q (38Q), while the black-rimmed arrowhead shows endogenous TBP (eg). Red and blue arrowheads indicate N-terminal (n_38Q_1-3) and C-terminal (c1–3) TBP fragments, respectively. The dashed rectangle highlights the gel section excised for MS analysis of TBP fragments. **c** Overview of semi-specific peptides detected by MS. Yellow dashed lines show 13 CAPN1 cleavage sites, while pink dashed lines indicate five chymotrypsin cleavage sites along the myc-TBP sequence. **d** In silico prediction of calpain cleavage sites in TBP (UniProt ID: P20226) using the GPS-CCD tool, overlaid with expected chymotrypsin cleavage sites. Black lines show normalised calpain cleavage likelihood score (nCCLS) along the protein sequence (left *y* axis), with the black-dotted line indicating the likelihood threshold (nCCLS = 1). Yellow lines represent the proportion of experimental runs (*n* = 3 experiments, right *y* axis) in which CAPN1 cleavage-specific peptides were detected by MS. Pink dashed lines mark chymotrypsin cleavage sites. The polyQ region in TBP (highlighted in light blue) produces a prediction artefact with high CCL scores. **e** MS spectrum of a calpain-specific peptide identified in all three experimental runs, representing CAPN1 cleavage at T106 | S107 in TBP. **f** In silico prediction of calpain cleavage likelihood between aa 95 and 115 in TBP without (light blue) and with cleavage site mutation (CSM) by quadruple tryptophan (WWWW; green) substitution around the detected cleavage T106 | S107 (S105-Q108). Black-dotted line marks the likelihood threshold (nCCLS = 1). **g**, **h** Western blot-based fragmentation analysis of myc-tagged TBP 38Q (**g**) and 106Q (**h**) without and with CSM, after myc tag-specific immunoprecipitation (IP) from 293T cell extracts, followed by a time-dependent CCA with CAPN1. Membranes were detected using N-terminally binding (D5C9H) and C-terminally binding (58C9) TBP antibodies. Light blue- and pink-rimmed arrowheads mark full-length TBP 38Q and 106Q, respectively, and green-rimmed their respective CSM forms. Red and blue arrowheads indicate N-terminal and C-terminal (n1–3; c1–3) TBP fragments, respectively. Green arrowheads indicate alternative TBP fragments caused by the presence of the CSM. The approximate binding sites of the antibodies used are illustrated above the corresponding blots. SE short exposure, LE long exposure. Source data are provided as a Source Data file.

To identify calpain-1 cleavage sites within TBP, we performed in vitro cleavage assays of TBP 38Q purified from 293T cells using calpain-1, followed by mass spectrometry (MS) analysis of excised TBP fragment bands after a chymotrypsin digest (Fig. 3a). Calpain-1 cleavage of TBP 38Q was confirmed via western blotting using the N-terminally binding D5C9H antibody and the C-terminally binding 58C9 antibody (Fig. 3b). Subsequent MS analysis identified 13 distinct peptides derived from myc-tagged TBP, each containing one terminus not attributable to chymotryptic cleavage (Supplementary Table 1). Comparison of these termini with in silico calpain cleavage site predictions for TBP (UniProt ID: P20226) using the GPS-CCD tool revealed that 10 peptides corresponded to predicted calpain cleavage sites within the TBP sequence, whereas three mapped to the C-terminal myc-tag and were excluded (Fig. 3c, d and Supplementary Table 1). Five of the identified peptides mapped to a region C-terminal of the polyQ tract, with their N-terminal sequences starting between residues Q104 and Q109 and extending to residue F122. All N-terminal sequences aligned with a motif of increased calpain cleavage likelihood located between residues A96 and Q117. Importantly, the peptide spanning S107 to F122 was the only one detected in all three experimental runs (Fig. 3d, e), indicating that a primary calpain-1 cleavage event takes place between T106 and S107. This cleavage is likely responsible for generating the observed TBP fragments n1 and c1. To validate this finding, we generated calpain cleavage site-mutant forms of myc-tagged TBP 38Q and 106Q, based on our previously established strategy^33,34^ and subjected them to in vitro cleavage using calpain-1. Western blotting confirmed that mutating amino acids surrounding the T106 | S107 site to tryptophan residues prevented the formation of the n1 and c1 fragments, while, expectedly, shifting cleavage towards adjacent sites (Fig. 3f, g and Supplementary Fig. 4).

### The C-terminal TBP fragment exhibits high turnover, while polyQ-containing N-terminal fragments show increased aggregation propensity

**Fig. 4:**
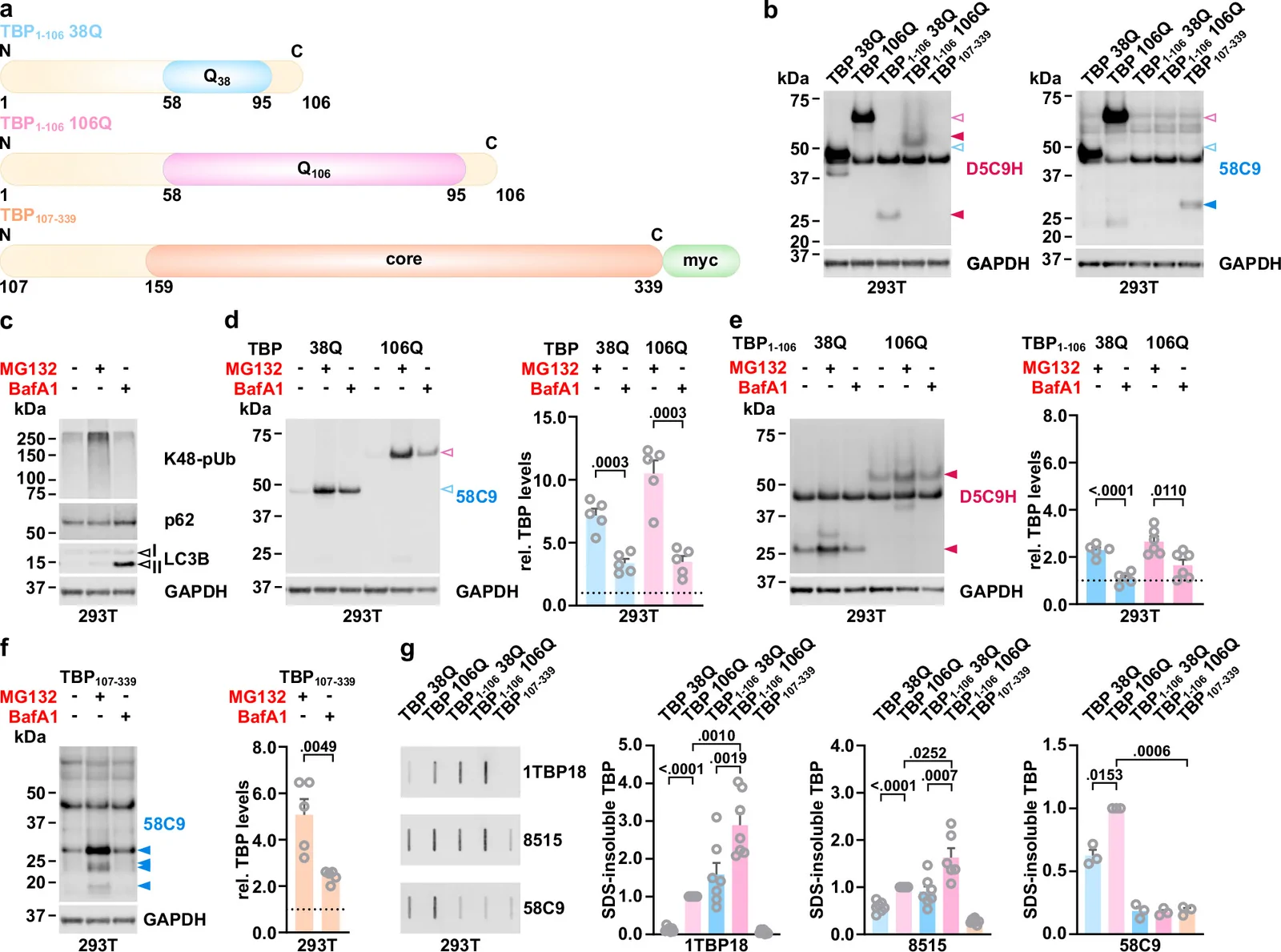
The C-terminal TBP fragment undergoes increased proteolytic turnover, while N-terminal polyQ-containing fragments are highly aggregation-prone. **a** Schematic representation of TBP fragment constructs derived from the calpain cleavage site T106 | S107. Two N-terminal fragments, non-polyQ-expanded (TBP_1-106_ 38Q) and polyQ-expanded (TBP_1-106_ 106Q), along with the corresponding myc-tagged C-terminal counterpart (TBP_107-339_), are depicted. **b** Protein extracts from 293T cells expressing TBP 38Q, TBP 106Q, TBP_1-106_ 38Q, TBP_1-106_ 106Q, or TBP_107-339_ were analysed by western blotting. Full-length TBP, as well as the N- and C-terminal fragment constructs, were detected using N-terminally binding (D5C9H) or C-terminally binding (58C9) TBP antibodies. GAPDH served as a loading control. Light blue- and pink-rimmed arrowheads mark full-length TBP 38Q and 106Q, respectively. Red arrowheads show N-terminal fragments (TBP_1-106_ 38Q or TBP_1-106_ 106Q), while blue arrowheads indicate C-terminal fragment TBP_107-339_. **c**–**f** 293T cells expressing full-length TBP or TBP fragment constructs were treated with the proteasome inhibitor MG132 or the autophagy inhibitor bafilomycin A1 (BafA1). Protein extracts were analysed by western blotting. Proteasome and autophagy inhibition were validated using antibodies against K48-linked polyubiquitin chains (K48-pUb), SQSTM/p62 (p62), and MAP1LC3B (LC3B) (in **c**). Black-rimmed arrowheads mark LC3B-I and -II. TBP 38Q, TBP 106Q, TBP_1-106_ 38Q, TBP_1-106_ 106Q, or TBP_107-339_ were detected using N-terminally binding (D5C9H) or C-terminally binding (58C9) antibodies (**d**–**f**). GAPDH served as a loading control. Densitometric analysis of the blots shows levels of full-length TBP (**d**) or TBP fragments (**e**, **f**). TBP levels were normalised first to GAPDH and then to the vehicle control. The vehicle control is indicated by a dotted line. *n* = 5 (**d**, **f**; **e** for TBP_1-106_ 38Q), or 6 (**e** for TBP_1-106_ 106) biological replicates. Unpaired Student’s *t* test. **g** Filter retardation assays of 293T cells expressing TBP 38Q, TBP 106Q, TBP_1-106_ 38Q, TBP_1-106_ 106Q or TBP_107-339_. SDS-insoluble TBP species were detected using N-terminally binding (1TBP18 or 8515) TBP antibodies or the C-terminally binding (58C9) TBP antibody. Densitometric analysis of SDS-insoluble full-length TBP and TBP fragments, normalised to full-length TBP 106Q signals within each sample set. *n* = 7 (1TBP18; 8515, except for TBP_1-106_ 106Q with *n* = 6 biological replicates), or 3 (58C9) biological replicates. One-sample *t* test and one-way ANOVA with Tukey’s post hoc test. Bars represent means + s.e.m. All tests were two-tailed. *P* values are shown in the graphs. Source data are provided as a Source Data file.

To explore the physiological and pathological roles of calpain-1-dependent TBP cleavage, we generated constructs of N- and C-terminal TBP fragments corresponding to the identified cleavage site T106 | S107 (Fig. 4a). First, we overexpressed full-length TBP 38Q or 106Q, non-polyQ-expanded (TBP_1-106_ 38Q) or polyQ-expanded (TBP_1-106_ 106Q) N-terminal TBP fragments, and their myc-tagged C-terminal (TBP_107-339_) counterpart in 293T cells. Protein extracts were analysed via western blotting. Using an N-terminally binding TBP antibody (D5C9H), we detected overexpressed full-length TBP as well as TBP_1-106_ 38Q and TBP_1-106_ 106Q. In addition, a C-terminally binding TBP antibody (58C9) confirmed the overexpression of TBP_107-339_. Notably, all fragment constructs exhibited markedly lower levels compared to full-length TBP (Fig. 4b). To determine whether high proteolytic turnover via cellular clearance mechanisms contributed to the low fragment levels, we treated transfected cells with proteasome inhibitor MG132 or autophagy inhibitor bafilomycin A1 (BafA1). Western blot analysis of K48-linked polyubiquitin chains (K48-pUb), as well as SQSTM1/p62 and MAP1LC3B, confirmed successful inhibition of proteasomal and autophagic pathways (Fig. 4c and Supplementary Fig. 5a). TBP-specific detection revealed a strong increase in levels of full-length TBP 38Q and 106Q upon proteasomal inhibition, whereas autophagy inhibition had only moderate effects (Fig. 4d). N-terminal fragments TBP_1-106_ 38Q and TBP_1-106_ 106Q showed minimal response to either proteasomal or autophagy inhibition (Fig. 4e). In contrast, levels of the C-terminal TBP_107-339_ fragment markedly increased with proteasomal inhibition, while autophagy inhibition had lesser impact (Fig. 4f). Given the possibility that increased aggregation propensity might explain the low levels on N-terminal TBP fragments, we performed filter retardation assays of protein extracts from transfected 293T cells to analyse SDS-insoluble species. Immunodetection with N-terminal antibodies 1TBP18 and 8515 revealed the presence of SDS-insoluble species of full-length TBP 106Q, as well as of TBP_1-106_ 38Q and TBP_1-106_ 106Q, with TBP_1-106_ 106Q exhibiting the strongest signal, while the C-terminal fragment as detected with antibody 58C9 did not aggregate (Fig. 4g). In summary, TBP fragments generated by calpain cleavage at T106 | S107 display reduced protein levels, driven by rapid proteasomal turnover of the C-terminal fragment and pronounced aggregation of N-terminal polyQ-containing fragments, particularly TBP_1-106_ 106Q.

### N-terminal TBP fragments are cytoplasmic and form perinuclear inclusion bodies

**Fig. 5:**
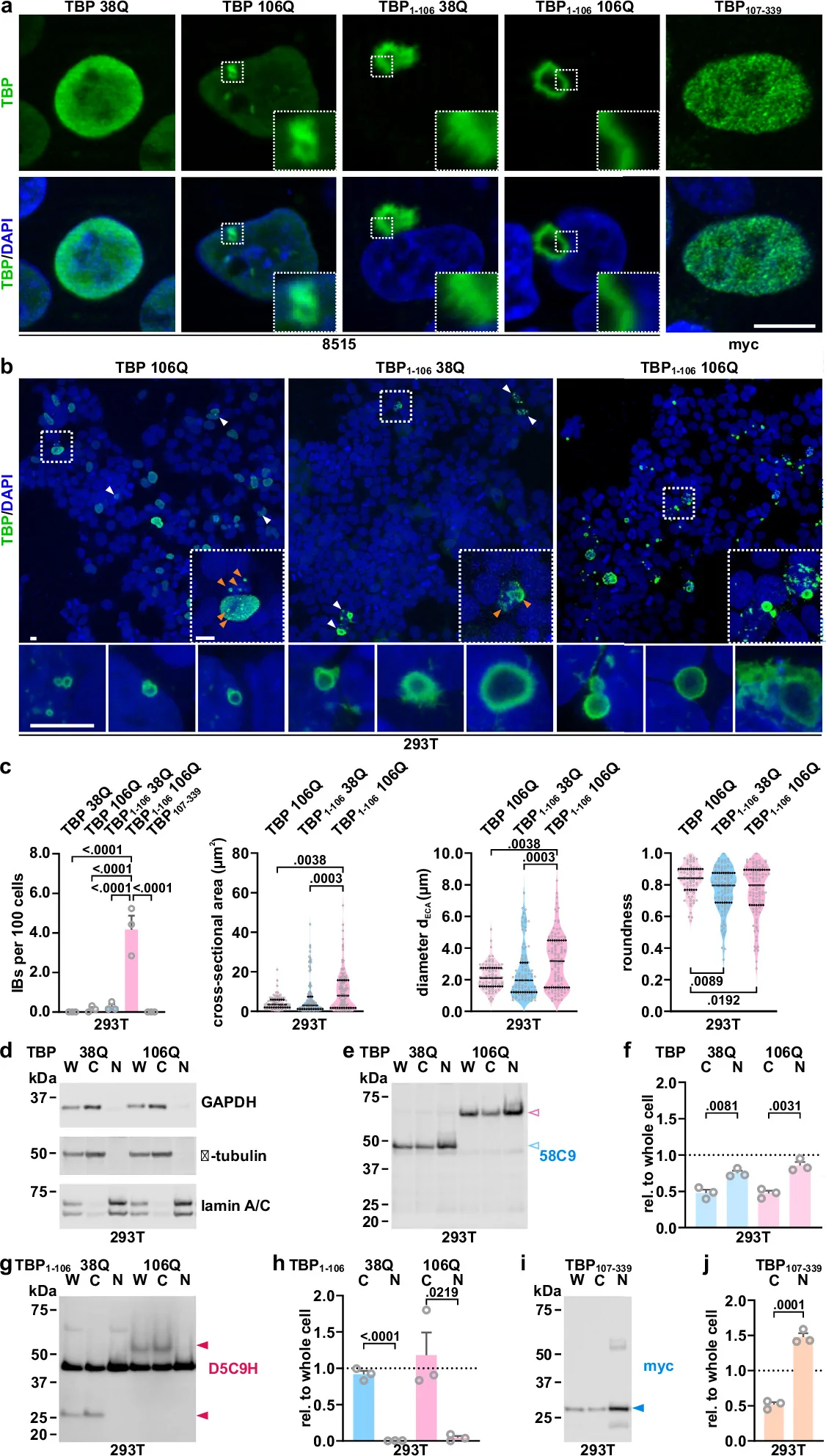
N-terminal polyQ-expanded TBP fragments show cytoplasmic localisation and form perinuclear inclusion bodies at a higher frequency. **a** Immunofluorescence staining and microscopy of 293T cells expressing TBP 38Q, TBP 106Q, TBP_1-106_ 38Q, TBP_1-106_ 106Q, or TBP_107-339_ (green). To analyse subcellular localisation and aggregation, an N-terminally binding TBP antibody (8515) or a myc tag-specific antibody (both in green) were used. DAPI was used as a nuclear counterstain (blue). Insets show 3.3× magnified views of selected regions (white dashed boxes). Scale bar: 10 µm. **b** Immunofluorescence staining and confocal microscopy of 293T cells expressing TBP 106Q, TBP_1-106_ 38Q, or TBP_1-106_ 106Q. The N-terminally binding TBP antibody 1TBP18 was used to analyse subcellular localisation of TBP inclusion bodies (IB; green). DAPI was used as a nuclear counterstain (blue). Maximum intensity projections (MIP) and insets of 3× magnified selected areas (white dashed boxes) are shown alongside three additional MIPs of representative TBP IBs. For TBP 106Q and TBP_1−106_ 38Q, IB-positive cells are highlighted with white arrowheads and individual IBs accented with orange arrowheads. Scale bars: 10 µm. **c** Fiji-based quantitative analysis of TBP IB frequency, cross-sectional area, equivalent circular area diameter (d_ECA_), and roundness. IB frequency was assessed as per 100 cells. *n* = 3 experiments. Bars represent means + s.e.m. Violin plots show the distribution of every single IB analysed. Dashed lines indicate median. Dotted lines indicate the 25^th^ and 75^th^ percentiles. One-way ANOVA with Tukey’s post hoc test or Kruskal–Wallis test with Dunn’s post hoc test. **d**–**j** 293T cells expressing TBP 38Q, TBP 106Q, TBP_1-106_ 38Q, TBP_1-106_ 106Q, or TBP_107-339_ were subjected to cellular-nuclear fractionation. Obtained fractions (whole cell (W); cytoplasmic fraction (C); nuclear fraction (N)) were analysed by western blot. Cytoplasmic GAPDH and α-tubulin, as well as nuclear lamin A/C, served as fraction-purity and loading controls (**d**). Western blots were probed with TBP binding antibodies 58C9 or D5C9H, or myc tag-specific antibody to detect TBP 38Q and TBP 106Q (**e**, **f**; blue-rimmed and pink-rimmed arrowheads), TBP_1-106_ 38Q and TBP_1-106_ 106Q (**g**, **h**; red arrowheads) or TBP_107-339_ (**i**, **j**; blue arrowheads). Densitometric analysis shows relative levels of full-length TBP or TBP fragments in the cytoplasmic and nuclear fractions. Levels were normalised first to fraction-specific loading controls and then to whole-cell (indicated by a dotted line) within each sample set. *n* = 3 biological replicates. Bars represent means + s.e.m. Unpaired Student’s *t* test. All tests were two-tailed. *P* values are shown in the graphs. Source data are provided as a Source Data file.

PolyQ-expanded TBP is known to form intranuclear inclusion bodies (IBs) in the brains of SCA17 patients^13^. As our findings demonstrate that N-terminal TBP fragments form SDS-insoluble species and exhibit enhanced aggregation propensities compared to the full-length protein, we investigated their aggregation behaviour in 293T cells expressing full-length TBP and TBP fragments using fluorescence microscopy. Consistent with our filter retardation analyses, TBP 106Q, TBP_1-106_ 38Q, and TBP_1-106_ 106Q formed nucleus-associated IBs, whereas TBP 38Q and TBP_107-339_ displayed a diffuse nuclear staining (Fig. 5a). Quantitative analysis supported by confocal microscopy revealed a significantly higher frequency of IB formation by TBP_1-106_ 106Q compared to TBP 106Q and TBP_1-106_ 38Q (Fig. 5b, c). IBs formed by TBP_1-106_ 106Q also exhibited a larger cross-sectional area and diameter. Additionally, structural differences were observed, with IBs formed by TBP_1-106_ 38Q and TBP_1-106_ 106Q showing reduced roundness (Fig. 5b, c). Strikingly, while IBs formed by TBP 106Q were mainly intranuclear, those formed by the N-terminal TBP fragments TBP_1-106_ 38Q and TBP_1-106_ 106Q exhibited a perinuclear localisation (Fig. 5b and Supplementary Fig. 5c, d). To explore whether the intracellular distribution of soluble TBP fragments underlies the observed spatial patterns of IBs, we conducted cytoplasmic-nuclear fractionation of 293T cells expressing full-length TBP and TBP fragments. As expected, full-length TBP was predominantly, while not solely, found in the nuclear fraction (Fig. 5d–f). In contrast, N-terminal TBP fragments were exclusively detected in the cytoplasmic fraction, which may account for the perinuclear localisation of the IBs they form (Fig. 5g, h). Conversely, the C-terminal fragment TBP_107-339_, which contains the DNA-binding core domain, is enriched in the nuclear fraction, consistent with microscopy data, while lower levels remain detectable in the cytoplasmic fraction (Fig. 5i, j and Supplementary Fig. 5b). In summary, whereas C-terminal TBP predominantly localises to the nucleus, N-terminal polyQ-expanded TBP fragments mislocalise to the cytoplasm, where their aggregation-prone nature promotes the formation of perinuclear IBs.

### TBP fragments trigger calpain overactivation and dysregulation of the cellular proteome

**Fig. 6:**
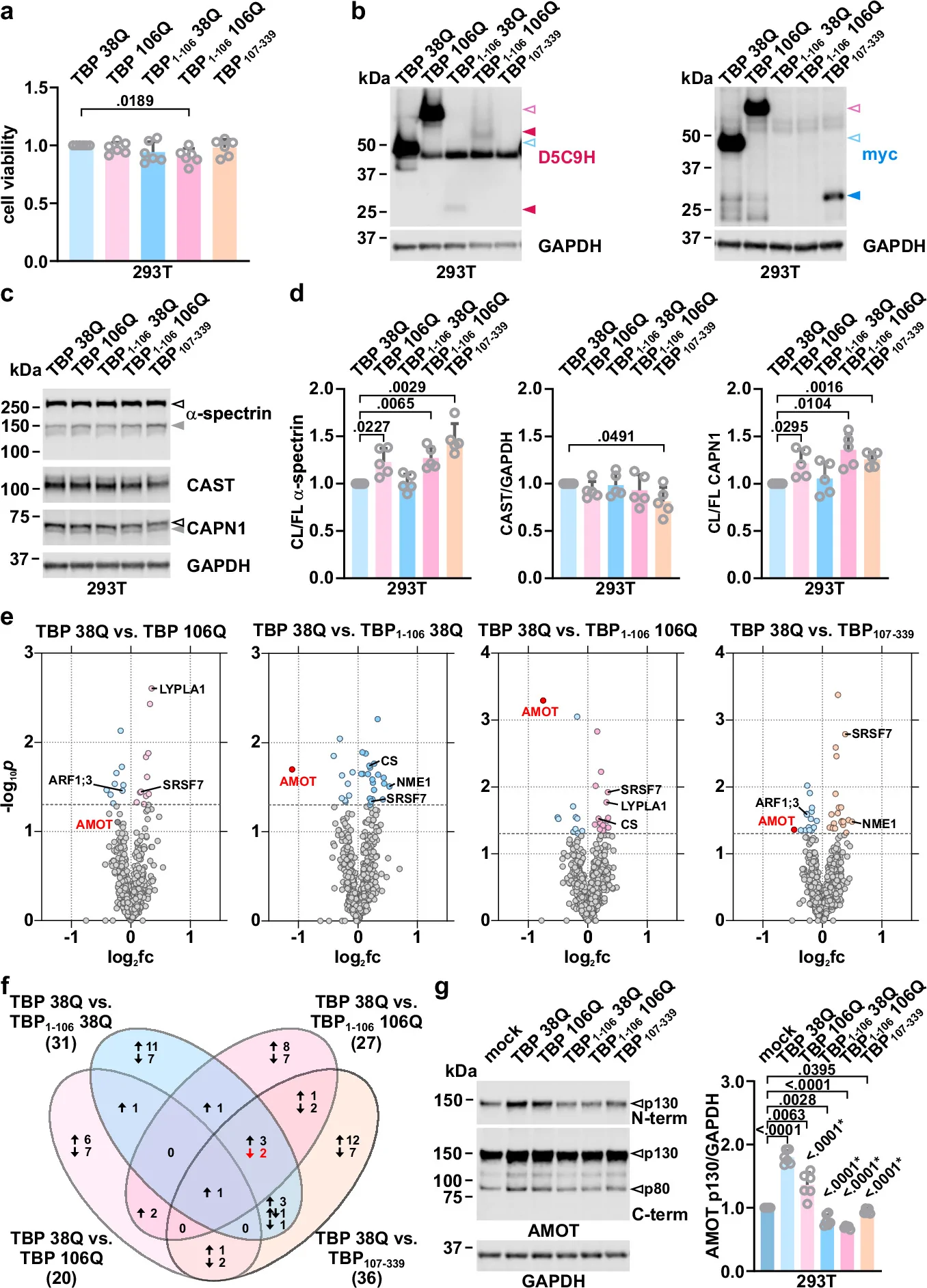
TBP fragments exhibit increased toxicity, induce calpain overactivation, and trigger proteomic alterations. **a** Resazurin-based cell viability assay of 293T cells expressing TBP 38Q, TBP 106Q, TBP_1-106_ 38Q, TBP_1-106_ 106Q, or TBP_107-339_. Signals were normalised to 293T cells expressing TBP 38Q. *n* = 6 biological replicates. One-sample *t* test. **b** Western blot analysis of 293T cells expressing TBP 38Q, TBP 106Q, TBP_1-106_ 38Q, TBP_1-106_ 106Q or TBP_107-339_. TBP was detected with D5C9H and myc-specific antibodies. GAPDH served as a loading control. Light blue- and pink-rimmed arrowheads mark full-length TBP 38Q and 106Q, respectively. Red arrowheads indicate N-terminal fragments (TBP_1-106_ 38Q or TBP_1-106_ 106Q), while blue arrowheads indicate C-terminal fragment TBP_107-339_. **c** Protein extracts of 293T cells expressing full-length TBP or TBP fragments were analysed for calpain activation by western blot. Immunodetection was conducted using antibodies against α-spectrin, calpastatin (CAST), and calpain-1 (CAPN1). GAPDH served as the loading control. Black-rimmed arrowheads indicate full-length proteins, while grey arrowheads show respective breakdown products. **d** Densitometric analysis of α-spectrin cleavage ratio (breakdown product (CL) normalised to full-length (FL) α-spectrin), CAST levels (CAST levels normalised to GAPDH) and CAPN1 cleavage ratio (breakdown product (CL) normalised to full-length (FL) CAPN1). Values were normalised to 293T cells expressing TBP 38Q in each measurement. *n* = 5 biological replicates. One-sample *t* test. **e** Label-free quantitative mass spectrometric analysis of protein extracts from 293T cells expressing full-length TBP and TBP fragments. Volcano plots show significance values (−log_10_*p*) plotted against the fold change (log_2_fc) of detected proteins. Proteins significantly altered (*P* ≤ 0.05; dotted horizontal line) in their levels are indicated in colour. Prominent hits are labelled. **f** Overlap of significantly dysregulated proteins in different conditions (293T cells expressing TBP 38Q, TBP 106Q, TBP_1-106_ 38Q, TBP_1-106_ 106Q or TBP_107-339_) shown by a Venn diagram. Black arrows indicate the direction of dysregulation. Red arrow includes AMOT. The Venn diagram was created using InteractiVenn. **g** Western blot analysis of AMOT levels in 293T cells expressing full-length TBP or TBP fragments, and in mock-transfected controls. An N-terminally binding AMOT antibody (N-term) and a C-terminally binding AMOT antibody (C-term) were used for immunodetection. GAPDH served as the loading control. Black-rimmed arrowheads indicate the p130 and p80 isoforms of AMOT. Densitometric analysis shows AMOT p130 levels normalised to the loading control. *n* = 6 biological replicates. One-sample *t* test and one-way ANOVA with Dunnett’s post hoc test (*). Bars represent means + s.e.m. All tests were two-tailed. *P* values are shown in the graphs. Source data are provided as a Source Data file.

To investigate whether TBP fragments generated by cleavage at T106 | S107 exhibit increased cytotoxicity and further activate calpains, in line with the toxic fragment hypothesis^18,19^, we assessed cell viability and calpain cleavage markers in 293T cells expressing full-length TBP or TBP fragments. Notably, only TBP_1-106_ 106Q caused a slight but significant decrease in cell viability (Fig. 6a), indicative of its inherent cytotoxicity. Western blot analysis of calpain markers α-spectrin, calpastatin, and calpain-1 revealed increased calpain activation in cells expressing full-length TBP 106Q and the N-terminal fragment TBP_1-106_ 106Q (Fig. 6b–d). Moreover, the DNA-binding C-terminal fragment TBP_107-339_ led to calpain overactivation, whereas the non-polyQ-expanded N-terminal TBP_1-106_ 38Q did not induce any changes (Fig. 6c, d). To gain broader insight into the downstream effects of TBP fragment expression and, in comparison, full-length TBP, we conducted a mass spectrometry-based label-free quantitative (LFQ) proteome analysis. Compared to TBP 38Q, overexpression of TPB 106Q in 293T cells resulted in a significant alteration of 20 proteins ( ↓ 9, ↑11). Analysis of cells expressing TBP_1-106_ 38Q, TBP_1-106_ 106Q, and TBP_107-339_ revealed 31 ( ↓ 10, ↑21), 27 ( ↓ 11, ↑16) and 36 ( ↓ 15, ↑21) differentially regulated proteins, respectively (Fig. 6e). A total of 21 proteins were dysregulated in at least two conditions, including ADP-ribosylation factor 1 (ARF1), citrate synthase (CS), lysophospholipase 1 (LYPLA1), and nucleoside diphosphate kinase A (NME1)—all of which have been implicated in the polyQ disease-related molecular pathogenesis^35–38^. Serine/arginine-rich splicing factor 7 (SRSF7) was the only protein consistently increased across all conditions relative to TBP 38Q (Fig. 6e, Supplementary Fig. 6a and Supplementary Data 1). Most prominently, levels of angiomotin (AMOT), a member of the motin family linked to cell motility, angiogenesis, and regulation of YAP1-dependent Hippo signalling^39^, were significantly reduced in 293T cells expressing TBP_1-106_ 38Q, TBP_1-106_ 106Q, or TBP_107-339_ compared to cells expressing full-length TBP 38Q (Fig. 6e, f). The most pronounced reductions were observed in cells expressing the N-terminal fragments TBP_1-106_ 38Q and 106Q. To confirm this reduction, we analysed AMOT—present as a polyQ-stretch-containing p130 (UniProt ID: Q4VCS5-1) and a p80 (UniProt ID: Q4VCS5-2) isoform—by western blotting of protein extracts from 293T cells expressing full-length TBP or TBP fragments, as well as mock controls transfected with an empty vector to assess the effect of TBP 38Q on AMOT baseline expression. Compared to mock-transfected controls, both AMOT p130 and p80 levels increased with TBP 38Q overexpression. When compared to TBP 38Q, AMOT p130 and p80 levels significantly decreased in TBP 106Q-, TBP_1-106_ 38Q-, TBP_1-106_ 106Q-, and TBP_107-339_-expressing cells, with TBP_1-106_ 106Q lowering AMOT below mock-transfected control levels (Fig. 6g and Supplementary Fig. 6b, c). Notably, subsequent protein network analysis, based on the differential proteome detected for TBP_1-106_ 106Q, revealed a protein interaction cluster linked to AMOT (Supplementary Fig. 6d), with all proteins implicated in neurological contexts (Supplementary Data 2).

### TBP fragments induce a multifactorial and polyQ-dependent depletion of AMOT

**Fig. 7:**
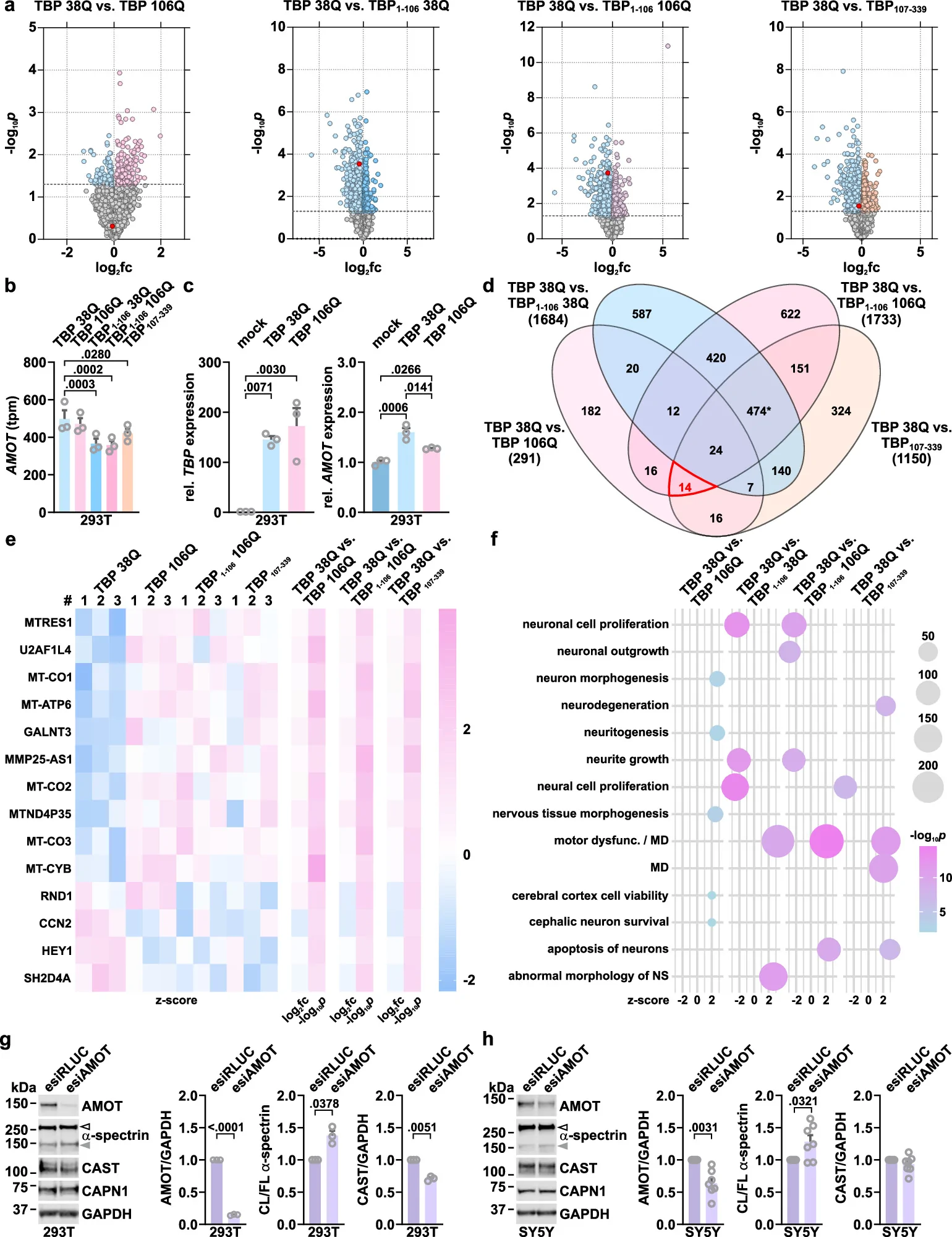
TBP fragments trigger transcriptional disturbances with repercussions on AMOT-YAP1 signalling, neuronal pathways, and calpain activation. **a** Transcriptomic analysis of 293T cells expressing TBP 38Q, TBP 106Q, TBP_1-106_ 38Q, TBP_1-106_ 106Q, or TBP_107-339_ by RNA sequencing. Volcano plots show −log_10_*p* values plotted against expressional fold changes (log_2_fc) of identified genes for comparisons against the TBP 38Q control condition. Significantly differentially expressed genes (DEGs) (*P* ≤ 0.05; dotted horizontal line) are visualised in colour. Red data points mark *AMOT*. *n* = 3 biological replicates. **b** Transcript levels of *AMOT* in 293T cells expressing TBP 38Q, TBP 106Q, TBP_1-106_ 38Q, TBP_1-106_ 106Q, or TBP_107-339_ detected by RNA sequencing and shown as transcripts per million (tpm). *n* = 3 biological replicates. For statistics, see Methods: RNA sequencing. **c** Quantitative PCR of *TBP* and *AMOT* expression in 293T cells transfected with TBP 38Q, TBP 106Q, or an empty vector. *n* = 3 biological replicates. One-way ANOVA with Tukey’s post hoc test. **d** Overlap of significant DEGs in TBP 106Q, TBP_1-106_ 38Q, TBP_1-106_ 106Q, or TBP_107-339_ compared to TBP 38Q visualised by Venn diagram. An asterisk marks the intersection, which includes *AMOT*. Red-rimmed intersection marks 14 DEGs shared between TBP 106Q, TBP_1-106_ 106Q, and TBP_107-339_ compared to TBP 38Q. The Venn diagram was created via InteractiVenn. **e** Heatmap of shared 14 DEGs (highlighted in red in **c**) showing row-wise calculated z-scores, log_2_fc, and −log_10_*p* of TBP 106Q, TBP_1-106_ 106Q, and TBP_107-339_ compared to TBP 38Q. *n* = 3 biological replicates. **f** Bubble plot of top 5 significantly up- and downregulated pathways in 293T cells expressing TBP 106Q, TBP_1-106_ 38Q, TBP_1-106_ 106Q, or TBP_107-339_. Data includes z-scores and presents −log_10_*p* indicated by bubble colour and number of pathway-related DEGs by bubble size. MD movement disorder, NS nervous system, dysfunct. dysfunction. **g**, **h** Western blot analysis of protein extracts from 293T or SH-SY5Y (SY5Y) cells transfected with esiRNA directed against *AMOT*. *Renilla luciferase* targeting esiRNA was used as a mock control. AMOT as well as calpain markers α-spectrin, calpastatin (CAST), and calpain-1 (CAPN1) were detected. GAPDH served as the loading control. Densitometric analysis shows AMOT and calpastatin levels normalised to GAPDH, as well as α-spectrin cleavage ratio (α-spectrin breakdown product (CL) indicated by grey arrowhead normalised to full-length (FL) α-spectrin indicated by black-rimmed arrowhead). *n* = 3 (293T) and *n* = 7 (SY5Y) biological replicates. One-sample *t* test. Bars represent means + s.e.m. All tests were two-tailed. *P* values are shown in the graphs. Source data are provided as a Source Data file.

Previous studies have demonstrated that polyQ-expanded TBP exhibits impaired DNA-binding abilities and altered self-dimerisation, leading to disruptions in its transcriptional activity^14,15,17,28^. To determine whether the observed reduction in AMOT protein levels results from TBP-induced transcriptional changes and to uncover affected pathways, we performed RNA sequencing of 293T cells expressing full-length TBP or TBP fragments. We identified 291 differentially expressed genes (DEGs) in TBP 106Q, 1684 DEGs in TBP_1-106_ 38Q, 1733 DEGs in TBP_1-106_ 106Q, and 1150 DEGs in TBP_107-339_, compared to TBP 38Q (Fig. 7a). Interestingly, *AMOT* expression was significantly downregulated in TBP_1-106_ 38Q, TBP_1-106_ 106Q, and TBP_107-339_ in our transcriptomics data, while TBP 106Q showed only a trend towards *AMOT* reduction (Fig. 7b). In contrast, the other two motin family members, angiomotin-like 1 (*AMOTL1*) and 2 (*AMOTL2*)^39^, were either significantly downregulated only in TBP 106Q-expressing cells or remained unaltered, respectively (Supplementary Fig. 7). To further evaluate the TBP-dependent modulation of *AMOT* expression, we performed qPCR analysis in 293T cells transfected with TBP 38Q, TBP 106Q, or an empty vector. Importantly, and corresponding to our western blot data, TBP 38Q increased expression of *AMOT*, while TBP 106Q showed a deficiency in *AMOT* upregulation (Fig. 7c).

Given that AMOT levels decreased upon TBP 106Q overexpression, we investigated additional cellular mechanisms responsible for this reduction. Since previous experiments demonstrated calpain overactivation in the presence of polyQ-expanded TBP and TBP fragments, we examined whether AMOT reduction could result from calpain-dependent proteolysis. In silico calpain cleavage site prediction for AMOT p130 (UniProt ID: Q4VCS5-1) using the GPS-CCD tool demonstrated several potential sites along the protein sequence (Supplementary Fig. 8a). To validate the prediction results, we activated calpains in 293T cells using ionomycin. Calpain activation was confirmed by increased cleavage of the calpain substrates α-spectrin and calpastatin. In ionomycin-treated cells, AMOT levels were strongly reduced, and fragment bands increased, while pretreatment with the calpain inhibitors ALLN, calpeptin, or CI-III rescued AMOT levels and reduced cleavage, demonstrating that this reduction was calpain-specific (Supplementary Fig. 8b). Next, we co-expressed AMOT with calpain-1 in 293T cells to confirm its calpain-dependent proteolysis. As expected, calpain-1 overexpression led to increased α-spectrin fragmentation, reduced calpastatin levels, and a significant reduction in AMOT, identifying AMOT as a direct calpain-1 substrate (Supplementary Fig. 8c). Conversely, calpain-1 knockdown reduced substrate cleavage and significantly increased AMOT levels (Supplementary Fig. 8d).

In addition, we explored whether AMOT levels declined due to recruitment into IBs formed by polyQ-expanded TBP or N-terminal TBP fragments, potentially driven by the polyQ stretch at the N-terminus of the p130 isoform. Fluorescence microscopy revealed that AMOT colocalised with IBs formed by N-terminal TBP fragments (Supplementary Fig. 9a), with ~25% of TBP IBs formed by TBP_1-106_ 38Q and TBP_1-106_ 106Q being AMOT-positive. In contrast, fewer AMOT-positive IBs were observed in cells expressing full-length TBP 106Q (Supplementary Fig. 9b). As we observed intracellular AMOT accumulations independent of TBP, we next assessed whether endogenous or overexpressed V5-tagged AMOT is intrinsically aggregation-prone. Using in situ and membrane-based IB fluorescence microscopy, we confirmed that AMOT co-aggregates with polyQ-expanded TBP_1-106_ 106Q, whereas AMOT alone does not form SDS-insoluble or ubiquitin-positive aggregates (Supplementary Fig. 9c–g). Taken together, our findings suggest that AMOT depletion arises from a combination of transcriptional dysregulation, calpain overactivation-dependent proteolysis, and recruitment into TBP IBs.

As both calpain overactivation and IB formation are hallmark features of several other polyQ disorders^18,40^, we examined whether overexpression of polyQ-expanded ataxin-3, the pathogenic driver of SCA3, would produce similar effects. While non-polyQ-expanded ataxin-3 15Q did not alter AMOT levels at baseline (Supplementary Fig. 10a)—contrasting our observation for TBP 38Q—overexpression of polyQ-expanded ataxin-3 148Q led to reduced levels of both AMOT p130 and p80, alongside increased calpain-dependent α-spectrin cleavage (Supplementary Fig. 10b). Further, we observed AMOT sequestration into ataxin-3-positive aggregates (Supplementary Fig. 10c), confirming a dual mechanism of AMOT depletion in the SCA3 context.

### PolyQ-expanded TBP and its fragments cause AMOT-YAP1 signalling disturbances, impacting neuronal pathways and calpain activity

To identify specific pathways disrupted by polyQ-expanded TBP and its fragments, we compared DEGs across different groups (Fig. 7d). A comparative analysis of TBP 106Q, TBP_1-106_ 106Q, and TBP_107-339_ revealed 14 commonly dysregulated genes, including *MT-CYB, MT-CO1, MT-CO2, MT-CO3, MT-ATP6*, which encode subunits of the mitochondrial respiratory chain complexes III to V (Supplementary Fig. 11a). In addition, *CCN2* was consistently downregulated across all three groups (Fig. 7e). Importantly, CCN2 is a downstream target of the transcriptional co-regulator Yes-associated protein 1 (YAP1), which interacts with AMOT^39^. Ingenuity Pathway Analysis (IPA) predicted YAP1 inhibition upon TBP_1-106_ 106Q overexpression, with a total of 89 YAP1 target genes showing dysregulation (Supplementary Fig. 11b and Supplementary Data 3). Based on these results, we hypothesised that polyQ-expansion and fragmentation of TBP dysregulated the AMOT-YAP1 pathway. To further evaluate this, we examined the presence of differentially expressed YAP1 target genes amongst DEGs shared by TBP 106Q and TBP_1-106_ 106Q, TBP 106Q and TBP_107-339_, as well as TBP_1-106_ 106Q and TBP_107-339_, and identified eight candidates: *RPL26*, *TNIP2*, *SGK1*, *IFI6*, *CCN2*, *ACTA2*, *PLK2*, and *RPL3L*. Notably, except for *PLK2* and *TNIP2*, six of these genes mapped to an extended protein network, revealing previously reported or predicted interactions (Supplementary Fig. 11c). Collectively, our findings suggest that polyQ-expanded and fragmented TBP induce transcriptional perturbations that disrupt the AMOT-YAP1 pathway and lead to differential expression of downstream target genes.

To assess the relevance of the identified DEGs to neurological function, we conducted an additional IPA analysis, which linked DEGs to several neurological processes, including neuronal cell proliferation, nervous tissue morphogenesis, and neurological disorders such as motor dysfunction, movement disorders, and abnormal nervous system morphology (Fig. 7f and Supplementary Data 4).

Finally, to further explore AMOT’s role in calpain overactivation, we performed AMOT knockdown experiments in 293T and SH-SY5Y cells—as a neuronal model—and analysed calpain system markers. In both cell lines, AMOT knockdown resulted in increased α-spectrin cleavage (Fig. 7g, h). Moreover, in 293T cells, calpastatin levels significantly decreased, suggesting that AMOT is functionally linked to calpain activation.

### SCA17 patient-derived cell models reproduce calpain overactivation, TBP fragmentation, and AMOT reduction

To validate our findings from overexpression cell models, we obtained fibroblasts from an SCA17 patient and an age- and sex-matched unaffected control subject and performed an initial analysis of TBP expression and AMOT levels. The SCA17 patient carries 37 CAG/CAA repeats on the wild-type allele and 52 repeats on the pathogenic expanded allele of *TBP*, whereas the control shows homozygous repeat lengths of 37 CAG/CAA repeats on both alleles (Supplementary Fig. 12a). Fluorescence microscopy demonstrated nuclear localisation of TBP and cytoplasmic localisation of AMOT (Supplementary Fig. 12b, c). Subsequent western blot analysis confirmed the presence of polyQ-expanded TBP in SCA17 fibroblasts and, importantly, revealed reduced AMOT p130 levels (Supplementary Fig. 12d, e), consistent with observations in 293T cells.

**Fig. 8:**
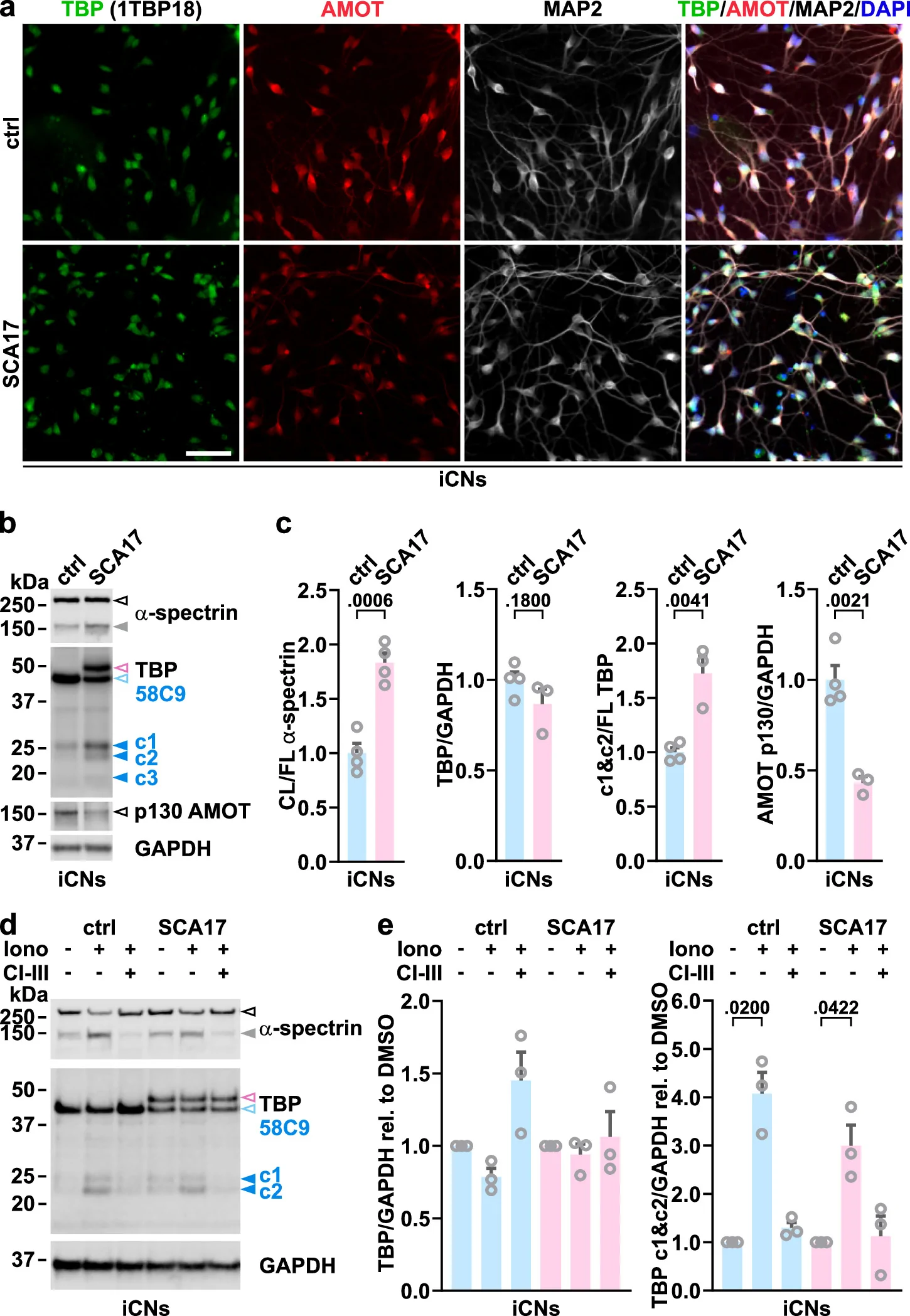
SCA17 patient-derived induced neurons recapitulate calpain overactivation, TBP cleavage, and AMOT reduction. **a** Fluorescence microscopy of induced cortical neurons (iCNs) of an unaffected individual and an SCA17 patient, immunostained for TBP (1TBP18, green), AMOT (red), and the pan-neuronal marker MAP2 (white). DAPI was used as a nuclear counterstain (blue). Scale bar: 50 µm. **b** Western blot analysis of α-spectrin cleavage as a calpain activation marker, TBP levels and fragmentation, and AMOT p130 levels in ctrl and SCA17 iCNs. GAPDH served as the loading control. Black-rimmed arrowheads indicate full-length (FL) proteins, while the grey arrowhead shows respective cleavage (CL) products. Light blue-rimmed arrowhead marks full-length non-polyQ-expanded TBP, pink-rimmed arrowhead polyQ-expanded TBP, and light blue arrowheads C-terminal TBP fragments c1–3. **c** Densitometric analysis of α-spectrin cleavage ratio (CL to FL α-spectrin), FL-TBP (normalised to GAPDH), cleaved TBP (c1&c2 normalised to FL-TBP), and AMOT p130 (normalised to GAPDH). *n* = 4 (CL to FL α-spectrin; all ctrl conditions) or 3 (SCA17 conditions of TBP normalised to GAPDH, c1&c2 normalised to FL-TBP, and AMOT p130 normalised to GAPDH) biological replicates. Student’s *t* test. **d** Ctrl and SCA17 iCNs were treated with ionomycin (Iono) for calpain activation and analysed by western blotting. Calpain inhibitor III (CI-III) treatment served as a specificity control. DMSO was added as a treatment control. Calpain activation was confirmed by detecting α-spectrin cleavage. Calpain-induced TBP fragmentation was detected using the C-terminally binding TBP antibody 58C9. GAPDH served as a loading control. Light blue-rimmed arrowhead marks full-length non-polyQ-expanded TBP, pink-rimmed arrowhead polyQ-expanded TBP, and light blue arrowheads c1 and c2. **e** Densitometric analysis of FL-TBP (normalised to GAPDH and DMSO control) and TBP fragmentation (c1&c2 normalised to FL-TBP and DMSO control). *n* = 3 biological replicates. Bars represent means + s.e.m. Two-tailed one-sample *t* test. *P* values are shown in the graphs. Source data are provided as a Source Data file.

To further validate these effects in a neuronal context, we generated iPSCs from SCA17 patient and control fibroblasts and differentiated them into induced cortical neurons (iCNs). Fluorescence microscopy confirmed the cortical identity of the differentiated neuronal cells via TBR1, CTIP2, βIII-tubulin, and MAP2 staining, as well as the expression of nuclear TBP and cytoplasmic AMOT, the latter also present in the neuronal projections (Fig. 8a and Supplementary Fig. 12f).

Baseline analysis of iCNs by western blotting revealed increased calpain activity in SCA17 cells, as indicated by enhanced α-spectrin cleavage and trends toward increased processing of calpain-1 and CAPNS1 (Fig. 8b, c and Supplementary Fig. 12g, i). Consistent with this, we observed increased TBP fragmentation in SCA17 iCNs, including the well-characterised C-terminal fragments c1, c2, and c3, as well as corresponding N-terminal cleavage products (Fig. 8b, c and Supplementary Fig. 12h, i). Importantly, SCA17 neurons showed reduced levels of AMOT p130 (Fig. 8b, c).

The calpain-dependent origin of the observed C-terminal TBP fragments was confirmed by performing ionomycin-induced calpain activation in control and SCA17 iCNs, followed by western blot analysis (Fig. 8d, e).

Together, the calpain overaction, TBP fragmentation, and AMOT depletion observed in overexpression-based models were recapitulated in patient-derived neurons, reinforcing their pathophysiological relevance in SCA17.

### SCA17 rat cerebellum shows AMOT reduction, YAP1 signalling disturbances, and proteomic perturbations with links to oligodendrocytes and neurofunction

**Fig. 9:**
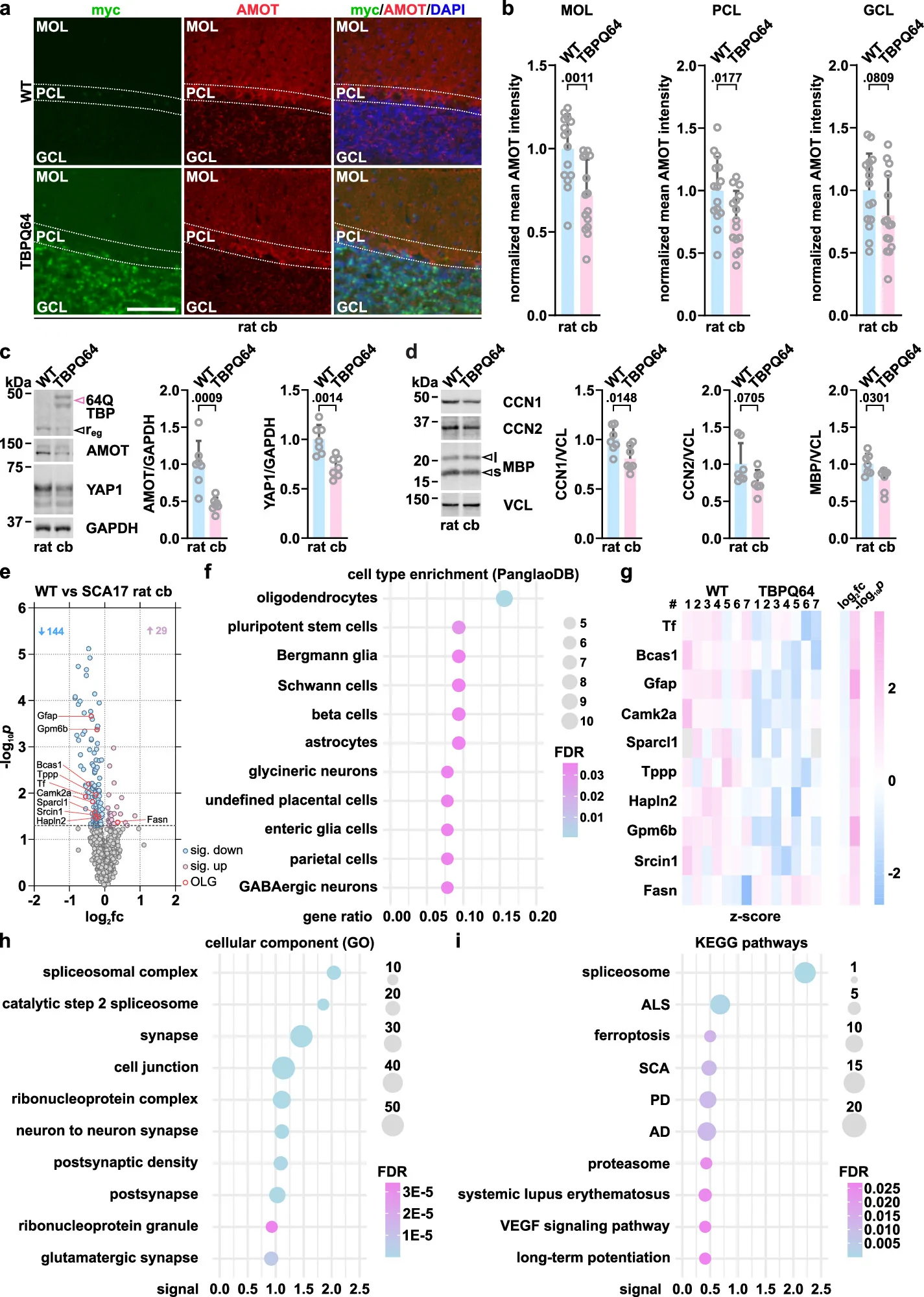
AMOT, YAP1, and its downstream targets are depleted in SCA17 rat cerebellum, causing oligodendrocyte-enriched proteome disturbances with neurofunctional impact. **a** Immunofluorescence staining and microscopy of cerebellum (cb) sections from 10-month-old wild-type (WT) and transgenic SCA17 (TBPQ64) rats expressing N-terminally myc-tagged, polyQ-expanded human TBP with 64 glutamines. Myc-TBP signals are shown in green, and AMOT signals in red. DAPI was used as a nuclear counterstain (blue). MOL molecular layer, PCL Purkinje cell layer, GCL granular cell layer. Scale bar: 100 µm. **b** Quantitative analysis of AMOT staining in the MOL, PCL, and GCL of WT and TBP64Q rat cerebellum. Signals were normalised to WT. *n* = 3 animals, 5 images per animal. Unpaired Student’s *t* test. **c** Western blot analysis of cerebellar protein extracts from WT and TBPQ64 rats. PolyQ-expanded (64Q, pink-rimmed arrowhead) and endogenous rat TBP (r_eg_, black-rimmed arrowhead), as well as AMOT and YAP1, were detected. GAPDH served as the loading control. Densitometric analysis shows AMOT and YAP1 levels in TBPQ64 rats relative to WT rats. Levels were normalised to GAPDH. *n* = 7 animals. Unpaired Student’s *t* test. **d** Western blot analysis of CCN1, CCN2, and MBP in WT and TBPQ64 rat cerebellum. Black-rimmed arrowheads mark long (l) and short (s) MBP isoforms. Vinculin (VCL) served as a loading control. Densitometric analysis shows protein levels normalised to VCL. *n* = 7 animals. Unpaired Student’s *t* test. **e** Label-free quantitative mass spectrometric analysis of cerebellar protein extracts from WT and SCA17 rats. *n* = 7 animals. Volcano plots show significance values (−log_10_*p*) plotted against the fold change (log_2_fc) of detected proteins. Proteins significantly altered (*P* ≤ 0.05; dotted horizontal line) in their levels are indicated in colour. PanglaoDB-curated oligodendrocyte (OLG) marker proteins are labelled. **f** PanglaoDB-based cell type enrichment analysis for the significantly differentially regulated proteome in TBPQ64 rat cerebellum. Bubble plots show ranking by gene ratio, with bubble colour indicating FDR and bubble size gene count. **g** Heatmap of differentially regulated OLG marker proteins in TBPQ64 rat cerebellum, showing row-wise calculated z-scores, log_2_fc, and −log_10_*p*. *n* = 7 animals. **h**, **i** Bubble plot of top ten enrichments in the TBPQ64 rat cerebellum proteome for Cellular Component (Gene Ontology; in **h**) and KEGG pathways (**i**) obtained from STRING (string-db.org), ranked by signal. ALS amyotrophic lateral sclerosis, SCA spinocerebellar ataxia, PD Parkinson’s disease, AD Alzheimer’s disease. Bubble colour indicates FDR and bubble size protein counts. Bars represent means + s.e.m. All tests were two-tailed. *P* values are shown in the graphs. Source data are provided as a Source Data file.

To validate the observed AMOT reduction in the context of polyQ-expanded TBP in vivo, we analysed cerebellar tissue from our SCA17 TBPQ64 rats^28,41^ by fluorescence microscopy and western blot. In this model, we confirmed the previously reported calpain overactivation (Supplementary Fig. 13a) and demonstrated the presence of N-terminal fragments of transgenic myc-tagged TBP in the cerebellum, using a myc-IP-based enrichment and a selection of TBP-specific antibodies (Supplementary Fig. 13b–e). Importantly, immunofluorescence staining and microscopy of rat brain sections showed not only reduced AMOT signals, particularly in the molecular and Purkinje cell layers of the TBPQ64 cerebellum (Fig. 9a, b and Supplementary Fig. 14a–c), but also a decreased YAP1 intensity (Supplementary Fig. 14d). These effects were further validated by western blot analysis of cerebellar protein extracts (Fig. 9c and Supplementary Fig. 15a). Moreover, the YAP1-associated Hippo signalling target genes CCN1/CYR61^42^ and CCN2/CTGF^43^ were likewise reduced (Fig. 9d).

Given that loss of CCN1 has been associated with impaired myelination^44^, we assessed myelin basic protein (MBP) levels in cerebellar extracts and found a significant reduction in total MBP (Fig. 9d and Supplementary Fig. 15b). To further investigate SCA17-associated alterations on a broader scale, we performed LFQ proteomics, identifying 144 downregulated and 29 upregulated proteins compared to wild-type animals (Fig. 9e). While insufficiently covering YAP1 signalling-related targets in the significantly dysregulated proteome (Supplementary Fig. 15c), the majority of dysregulated proteins mapped to a large interaction cluster (Supplementary Fig. 16).

Notably, cell-type enrichment analysis revealed a significant overrepresentation of dysregulated proteins associated with oligodendrocytes, indicating a pronounced impact of SCA17 pathology on this cell type and, consequently, on myelination-related processes (Fig. 9e–g and Supplementary Fig. 15d). Moreover, pathway and gene enrichment analyses highlighted strong associations with neuron function and movement disorder-related pathways, while additionally uncovering dysregulations in mRNA processing-related mechanisms (Fig. 9h, i and Supplementary Fig. 15e, f), further supporting the validity of the TBPQ64 rat as a model for SCA17 and the impact of polyQ-expanded TBP on these processes.

### Amot is reduced in zebrafish expressing TBP 106Q, while its genetic depletion disrupts YAP1 signalling and induces a motor neuron phenotype

**Fig. 10:**
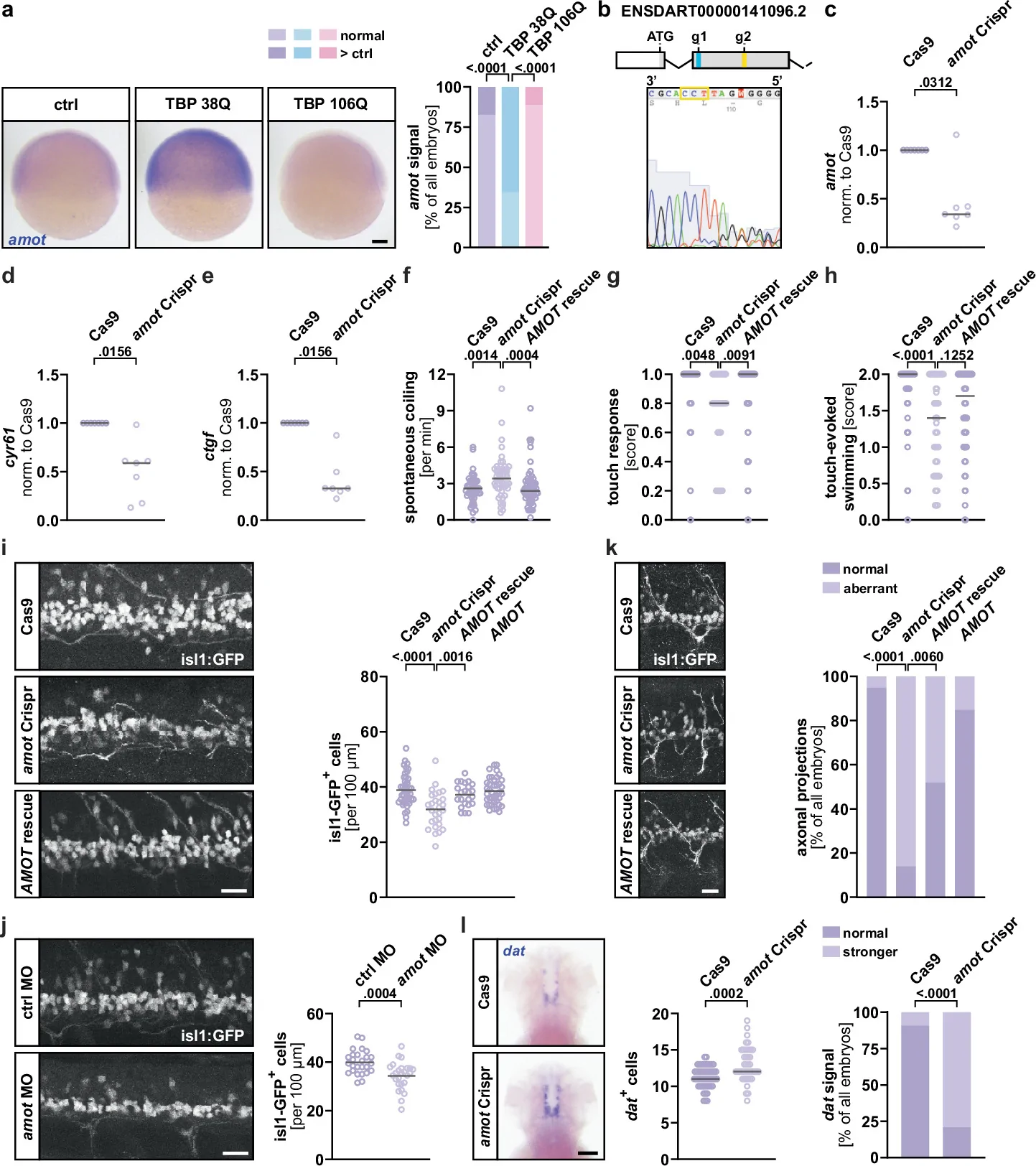
*Amot* loss-of-function in zebrafish affects Yap1-Hippo signalling, interfering with normal motor function and motor neuron development. **a** In situ hybridisation for *amot* in 60% epiboly embryos, injected with a control RNA (ctrl) or RNA encoding human TBP 38Q or TBP 106Q. *n* = 2 experiments with 69 (ctrl), 61 (TBP 38Q), and 53 (TBP 106Q) embryos in total. Fisher’s exact test. **b** Two guideRNAs (gR1 and gR2) were designed against exon 2 of the *amot* gene. PCR and subsequent Sanger sequencing revealed that editing occurred 3 bases upstream of the PAM sequence (yellow box) of gR2. **c** qPCR analysis of *amot* expression in 24 hours post fertilisation (hpf) embryos, injected either with Cas9 alone as control (Cas9) or a ribonucleoprotein consisting of both *amot*-specific guideRNAs and Cas9 (*amot* Crispr). *n* = 7 experiments. Wilcoxon test. **d**, **e** qPCR analysis of Hippo-Yap1 signalling target genes *cyr61* (*ccn1*) and *ctgf* (*ccn2*) in 24 hpf Cas9-injected control and *amot* crispant embryos. *n* = 7 experiments. Wilcoxon test. **f** *Amot* crispants (*amot* Crispr) display more spontaneous movements than control-injected (Cas9) embryos or embryos co-injected with RNA encoding human HA-tagged *AMOT* (*AMOT* rescue). *n* = 3 experiments with 54 (Cas9), 53 (*amot* Crispr), and 61 (*AMOT* rescue) embryos per condition. Kruskal–Wallis test with Dunn’s post hoc test. **g** *Amot* crispants (*amot* Crispr) show reduced touch response compared to Cas9 controls and *AMOT* rescue embryos. *n* = 3 experiments with 38 (Cas9), 40 (*amot* Crispr), and 58 (*AMOT* rescue) embryos per condition. Kruskal–Wallis test with Dunn’s post hoc test. **h** Swimming ability is reduced in *amot* Crispr embryos compared to Cas9 controls and *AMOT* rescue embryos. *n* = 4 experiments with 56 (Cas9), 58 (*amot* Crispr), and 78 (*AMOT* rescue) embryos per condition. Kruskal–Wallis test with Dunn’s post hoc test. **i** *Amot* crispants of tg(isl1:gfp) embryos have fewer motor neurons at 72 hpf than Cas9 controls, and *AMOT* rescue. *AMOT* overexpression (*AMOT*) does not alter motor neuron numbers. Images show maximal projections of confocal z-stacks. Scale bar: 20 µm. Isl1-GFP-positive (Isl1-GFP^+^) cells were quantified for all conditions. *n* = 3 with 43 (Cas9), 28 (amot Crispr), 23 (AMOT rescue), and 39 (*AMOT*) embryos in total. One-way ANOVA with Šídák’s post hoc test. **j** Antisense morpholino oligonucleotide (MO)-mediated knockdown of *amot* (*amot* MO) decreases motor neuron number in tg(isl1:gfp) embryos at 72 hpf compared to embryos injected with a control MO (ctrl MO). Isl1-GFP-positive (Isl1-GFP^+^) cells were quantified for all conditions. *n* = 3 experiments with 26 embryos in total. Welch’s test. Grey horizontal lines represent the mean. **k** *Amot* tg(isl1:gfp) crispants display axon guidance defects as shown for axons extending from motor neurons in comparison to Cas9 controls, *AMOT* rescue, and *AMOT* overexpression (*AMOT*) embryos. Scale bar: 20 µm. *n* = 3 experiments with 43 (Cas9), 28 (amot Crispr), 23 (AMOT rescue), and 39 (*AMOT*) embryos in total. Fisher’s exact test. **l** In situ hybridisation of 72 hpf embryos for the dopamine transporter (*dat*) demonstrates an increase in the number of dopaminergic neurons and higher levels of *dat* in *amot* crispants compared to Cas9-injected controls. Scale bar: 100 µm. *n* = 3 experiments with 54 (Cas9) and 43 (*amot* Crispr) embryos in total. Mann–Whitney test (*dat*^+^ cells) and Fisher’s exact test (*dat* signal). Grey horizontal lines in graphs represent the median, unless stated otherwise. All tests were two-tailed. *P* values are shown in the graphs. Source data are provided as a Source Data file.

We turned to zebrafish to explore a potential role of Amot in the nervous system. While Amot has been characterised for its function during angiogenesis^45^, it has not been investigated outside the vascular system in zebrafish. Consistent with our cell model findings, overexpression of human TBP 38Q in zebrafish embryos (Supplementary Fig. 17a) induced strong *amot* expression. This was not achieved by TBP 106Q, which resulted in lower *amot* signals compared to TBP 38Q (Fig. 10a and Supplementary Fig. 17b, c), mirroring the reduced AMOT protein levels observed in SCA17 rat cerebellum compared to wild-type. In situ hybridisation revealed that *amot* is ubiquitously expressed during early developmental stages, later becoming restricted to early neuronal and mesodermal structures during organogenesis. When the body plan has been established, *amot* transcripts can be detected in the developing vascular system and in the neural tube (Supplementary Fig. 17d), suggesting its involvement not only in angiogenesis, but also in neurogenesis. To analyse this, we designed a loss-of-function approach using transient Crispr/Cas9-mediated gene editing (Fig. 10b, c). As this may result in mosaic gene editing and can hence increase the variability of the phenotype, we genotyped each embryo. Importantly, Crispr/Cas9 against *amot* resulted in downregulation of typical Yap1-associated Hippo signalling target genes *ctgf* and *cyr61* (the zebrafish homologs of CCN1 and CCN2, respectively)^46^ consistent with a previous report^47^ (Fig. 10d, e), while not causing malformations in the embryos (Supplementary Fig. 16e–g). As a measure of ataxia, we performed simple behavioural tests routinely used to assess mechanosensory and motoric functions in zebrafish embryos^48,49^. Depletion of *amot* by Crispr/Cas9 manifested in more spontaneous movements in 24 hours post fertilisation (hpf) zebrafish embryos as well as loss of touch response and touch-evoked swimming at 48 hpf, suggesting general motor impairments, which were rescued by overexpression of human AMOT (Fig. 10f–h and Supplementary Fig. 17h). Moreover, these disturbances were accompanied by a small, but robust reduction in the number of motor neurons (Fig. 10i), which was reversed by AMOT overexpression in *amot* crispants, while overexpression of AMOT in controls had no effect. We confirmed the reduced number of motor neurons with a previously published^45^, established antisense morpholino oligonucleotide (MO)-mediated knockdown approach (Fig. 10j). In addition, we observed that axons outgrowing from the motor neuron’s cell bodies of *amot* crispants followed erratic paths, suggesting axon guidance defects (Fig. 10k). As in the context of HD, a related polyQ-dependent condition, changes in the dopaminergic system have been reported^50–52^, we additionally examined the zebrafish brain. Here, loss of Amot impacted the dopaminergic system by increasing the number of dopaminergic neurons, which also displayed a higher expression of the dopamine transporter (*dat*), a protein responsible for dopamine reuptake from the synaptic cleft into the presynapse (Fig. 10l). Taken together, these data link Amot to Yap1-associated Hippo signalling and support a role for Amot in neural development.

## Discussion

In this study, we confirmed calpain-dependent TBP fragmentation, identifying T106 as a major cleavage site located C-terminal to the polyQ tract. C-terminal TBP fragments were predominantly nuclear and exhibited high proteostatic turnover, whereas N-terminal fragments containing the polyQ tract mislocalised to the cytoplasm, where they formed perinuclear IBs. PolyQ-expanded N-terminal fragments were cytotoxic and further overactivated calpains. Multi-omics analysis revealed TBP fragment-driven dysregulation of neuronal pathways, highlighting AMOT and its downstream YAP1 signalling as key players in SCA17-linked neurodegeneration, which was validated in vivo.

Earlier studies reported TBP fragmentation in SCA17 and AD without linking it to proteolysis^8,15,53^. We previously demonstrated calpain-mediated TBP cleavage in SCA17 models and showed that calpain inhibition alleviates molecular pathology. However, the precise cleavage sites and the resulting N-terminal fragments remained unidentified^28^. In the present study, we confirmed our initial findings and demonstrated that calpain-dependent TBP cleavage—given the predominantly cytoplasmic localisation of these proteases—is a non-nuclear process that affects cytoplasmic TBP after translation but prior to its reported nuclear import^30^. However, further research is required to determine whether this mechanism is conserved across cell types and tissues. By refining our calpain cleavage site analysis^33^, we confirmed the generation of N-terminal, polyQ-containing fragments, as well as their presence in vivo, and identified T106 | S107 as a major cleavage site. Constructs mimicking cleavage at T106 | S107 allowed us to characterise the functional and pathological properties of TBP fragments.

C-terminal TBP fragments containing the DNA-binding core domain localised to the nucleus—contrary to our earlier findings^28^, which may be explained by a misidentification of the previously observed fragments in cytoplasmic fractions due to the lack of calpain-specific control conditions. However, the current observation is consistent with predicted C-terminal nuclear localisation signals^15^ and previously reported karyopherin-mediated nuclear shuttling in yeast, which depends on a recognition motif located near the protein’s C-terminal core domain^54,55^. In contrast, N-terminal polyQ-containing fragments mislocalised to the cytoplasm, aligning with findings from internally truncated TBP variants^15^.

We observed rapid proteasomal turnover of full-length and C-terminal TBP, consistent with prior studies of TBP regulation^56^. However, inhibition of protein clearance pathways did not increase the low levels of N-terminal fragments, likely due to their lack of lysine residues required for canonical ubiquitination. Instead, these fragments—particularly the polyQ-expanded ones—were highly prone to aggregation, forming perinuclear IBs. This differs from the smaller intranuclear IBs seen with full-length TBP in SCA17 patients and models^13,14,17,57^. Notably, and consistent with our data, a non-polyQ-expanded N-terminal splice variant of TBP has been shown to aggregate into cytoplasmic and perinuclear inclusions in AD brains^8,53^, suggesting that both TBP fragmentation and aggregation may play a broader role in neurodegenerative diseases.

Transcriptional dysregulation in SCA17 has previously been linked to polyQ expansion-induced disruptions in TBP dimerisation and interactions with transcriptional regulators such as TFIIB, XBP1, and SP1, identifying targets like HSPB1, MANF, and INPP5A^14,16,17^. Our multi-omics approach revealed that TBP fragments induced widespread proteomic and transcriptomic changes affecting pathways related to neuronal function and neurodegeneration. These included mitochondrial gene dysregulation, consistent with polyQ- or calpain-associated mitochondrial dysfunction observed in SCA3, HD, and AD^58–60^.

Among the affected factors, we identified AMOT as significantly downregulated in cells expressing polyQ-expanded TBP or its fragments, whereas non-polyQ-expanded TBP appeared to promote AMOT expression, as demonstrated in TBP-overexpressing 293T cells compared to mock-transfected controls at both the mRNA and protein level. AMOT reduction appears to be multifactorial, resulting from transcriptional dysregulation, co-aggregation, and calpain-dependent fragmentation. We reproduced these molecular findings in SCA17 patient-derived neurons, which, in addition to calpain overactivation and increased TBP fragmentation, exhibited a significant reduction in AMOT. In SCA17 rats, the presence of polyQ-expanded TBP similarly led to reduced AMOT levels, particularly in the molecular layer of the cerebellum. A comparable regulatory pattern was observed in zebrafish: overexpression of non-polyQ-expanded TBP increased *amot* mRNA levels, whereas polyQ-expanded TBP failed to promote *amot* expression, suggesting an evolutionarily conserved mechanism.

AMOT is known to be essential for dendritic tree complexity and locomotor coordination^61^, and we found its reduction to be additionally associated with both calpain-mediated cleavage and sequestration into IBs. While we have observed calpain-mediated cleavage of AMOT in our experimental models, N-terminal processing by the protease DDI2 has been shown to alter its role in collective cell migration^62^. Our findings raise the possibility that calpain-mediated cleavage may also contribute to AMOT regulation in neuronal cells. We speculate that the co-aggregation of AMOT into TBP-positive IBs observed in this study may be promoted by the presence of an N-terminal polyQ stretch in AMOT. Consistent with this, we also observed reduced AMOT levels upon overexpression of polyQ-expanded ataxin-3, likely driven by co-aggregation and calpain overactivation, whereas non-polyQ-expanded ataxin-3 did not affect AMOT expression. Together, these findings suggest that AMOT dysregulation may not be specific to SCA17 but could represent a common feature of polyQ disorders involving calpain overactivity and aggregation-prone proteins^18^. Moreover, AMOT knockdown in 293T and neuronal SH-SY5Y cells led to increased calpain activity, indicating a regulatory interplay between AMOT and the calpain system.

AMOT depletion resulted in dysregulation of YAP1 signalling, affecting key neuronal targets such as CCN1 and CCN2, both of which are essential for neuronal development and function^63^. Notably, CCN1 has recently been linked to neural circuit stabilisation and myelination in the adult brain^44^.

Importantly, we observed reduced levels of YAP1 and CCN1 in the SCA17 rat cerebellum, accompanied by decreased MBP. Proteomic analysis further revealed an overrepresentation of downregulated proteins associated with oligodendrocytes, the myelinating cells of the central nervous system, whose dysfunction has been implicated in spinocerebellar ataxias and HD^64,65^.

Subsequent pathway and gene enrichment analysis highlighted widespread proteomic alterations in the SCA17 rat cerebellum, with strong associations to neuronal dysfunction and movement disorder-related neurodegeneration but also mRNA metabolism.

To investigate the neuronal role of AMOT in vivo, we genetically depleted *amot* in zebrafish embryos. This resulted in downregulation of YAP1 target genes, impaired motor neuron development, and altered motor function. These findings align with previously observed motor neuron and locomotor phenotypes in an SCA3 zebrafish model^66,67^, and with studies implicating motor neuron involvement in certain spinocerebellar ataxias^68–70^. Interestingly, beyond its known function in angiogenesis^45^, *amot* transcripts were detected in developing neuronal structures, indicating a potential role in neurogenesis.

Very little is known about early cellular or molecular changes in the development of SCA17. When the disease has progressed, however, a reduction in dopamine transporter expression or activity has been reported^71^. Our study shows that *amot* depletion increases dopamine transporter levels and produces a higher number of dopaminergic neurons—features also described in the context of HD^50–52^. Importantly, overexpression of the dopamine transporter has been shown to eventually cause dopaminergic neuron loss^72^. Thus, we speculate that early upregulation of the dopaminergic system may precede the loss of dopaminergic activity observed at later stages. As proposed for HD^52^, these neurodevelopmental abnormalities may contribute to early disease stages in SCA17 or other neurodegenerative disorders characterised by dysregulation of the AMOT-YAP1 signalling axis.

In summary, our study shows that calpain-mediated TBP fragmentation in SCA17 leads to a multifactorial depletion of AMOT, linking this process to both disrupted neurodevelopment and neurodegeneration. These findings suggest that AMOT deficiency, resulting from calpain activity and TBP dysfunction, may represent a shared mechanism contributing to neuronal pathology.

## Methods

### Ethics declarations

For experiments with SCA17 patient- and unaffected subject-derived fibroblast lines, informed written consent was required and obtained from all individuals or their legal guardians. All procedures involving human participants were conducted in accordance with the principles of the Declaration of Helsinki, and sampling was performed in accordance with the relevant local guidelines and regulations. The control line originated from a study approved by the Ethics Committee of the University of Tübingen (vote 598/2011BO1). All experiments involving wild-type and SCA17 rats were conducted after approval by the local ethics committee at the Regierungspräsidium Tübingen, in accordance with the German Animal Welfare Act and the guidelines of the Federation of European Laboratory Animal Science Associations, based on European Union legislation (Directive 2010/63/EU). Zebrafish husbandry and experiments in this study were approved by local authorities (Veterinary Care Unit at the University of Tübingen and the animal welfare commissioner of the regional board for scientific animal experiments in Tübingen, Germany). Experiments were conducted in agreement with the European Union Directive 86/609/EEC for the protection of animals used for experimental and other scientific purposes.

### Sex as a biological variable

Sex was not considered a biological variable in this study. The SCA17 patient-derived fibroblast and iPSC line, as well as derived neurons, were analysed alongside age- and sex-matched control lines from an unaffected individual. All SCA17 rat tissues were derived from male individuals. Previous characterisation showed comparable phenotypes in both sexes^41^; therefore, sex was not included in the study design and is not expected to influence the measured parameters. Zebrafish experiments were conducted at embryonic stages prior to gonad formation, at which point biological sex is not yet established.

### Plasmids and siRNAs

To overexpress human TBP in cells (UniProt identifier: P20226-1), pcDNA3.1 vectors carrying cDNA for C-terminally myc-tagged full-length TBP with 38 or 106 glutamines (myc-TBP 38Q or 106Q), their calpain cleavage site-mutated (S105-S108 > WWWW) forms, or respective N-terminal or C-terminal fragment constructs of TBP were synthesised (BioCat, Heidelberg, Germany). For the generation of capped RNA for zebrafish injection, untagged human TBP 38Q and 106Q, respectively, were subcloned into pCS2+ plasmids. Additionally, pcDNA3.1 vectors coding for C-terminally myc-tagged ataxin-3 isoform 1c (ATXN3; UniProt identifier: P54252-2) with 15Q or 148Q glutamines (myc-ATXN3 15Q or 148Q) were synthesised (BioCat) to overexpress human ataxin-3. The p3XFlag-CAPN1 construct was a gift from Yi Zhang (Addgene plasmid #60941; http://n2t.net/addgene:60941; RRID: Addgene_60941)^73^. The pcDNA3.1_nV5-AMOT (p130) was a gift from Michael Wehr (Addgene plasmid #172997; http://n2t.net/addgene:172997; RRID: Addgene_172997)^74^. Vectors were amplified in transformed *E. coli* DH5a cells and isolated using the QIAprep Spin Miniprep Kit (QIAGEN, Hilden, Germany).

For gene knockdown in cell models, MISSION® endoribonuclease-prepared small interfering RNAs (esiRNA) were used targeting human *AMOT* (esiAMOT; EHU129771, Merck, Darmstadt, Germany), human CAPN1 (esiCAPN1; EHU032581; Merck), human CAPN2 (esiCAPN2; EHU025391, Merck), as well as human CAST (esiCAST; EHU096291, Merck). As a control, an esiRNA targeting the *Renilla* luciferase gene *RLUC* (esiRLUC; EHURLUC, Merck) was employed. Information on esiRNA cDNA target sequences can be obtained from the corresponding product website.

### Cell culture

HEK 293T (293T) (ATCC: CRL-3216) and SH-SY5Y (SY5Y) (ATCC: CRL-2266) cells were maintained in Dulbecco’s modified Eagle’s medium (DMEM) supplemented with 10% (v/v) foetal bovine serum (FBS), 1% (v/v) non-essential amino acids (MEM NEAA) and 1% (v/v) Antibiotic-Antimycotic (A/A) (all Gibco®, Thermo Fisher Scientific, Waltham, MA, USA) in 5% CO_2_ at 37 °C. 293T and SY5Y cells were transfected for 72 h using TurboFectin 8.0 (OriGene Technologies, Inc., Rockville, MD, USA) or Lipofectamine 3000 Reagent (Thermo Fisher Scientific), according to the manufacturer’s instructions.

Human fibroblast cell lines were established according to routine procedures after taking skin punch biopsies from an SCA17 patient as well as a sex- and age-matched unaffected control subject. Fibroblasts were grown in AmnioFroxx (Type A) culture medium (neoFroxx GmbH, Einhausen, Germany) in 5% CO_2_ at 37 °C.

### Fragment analysis by capillary electrophoresis

Genomic DNA was extracted from fibroblasts derived from an SCA17 patient and a sex- and age-matched unaffected control subject using the High Pure PCR Template Preparation Kit (REF 11796828001; Roche Diagnostics, Mannheim, Germany) according to the manufacturer’s protocol.

SCA17 alleles were PCR-amplified using the HotStarTaq PCR Master Mix (QIAGEN, Hilden, Germany) and fluorescently labelled primers (forward: JOE-5’-TGG AAG AGC AAC AAA GGC AG-3’; reverse: 5’-GGC CTG AGG TTC CCT GTG-3’; Metabion International AG, Planegg, Germany). Amplicon sizes were determined by capillary electrophoresis using a 3500xL Genetic Analyzer with the GeneScan™ 500 ROX™ dye Size Standard and the Pop-7™ polymer (all Applied Biosystems, Thermo Fisher Scientific). Data were analysed using GeneMapper™ Software version 6.1 (Applied Biosystems, Thermo Fisher Scientific). CAG repeat lengths were calculated by subtracting the number of bases outside the repeat region (87 bp) from the product size of the most intense peak and dividing the result by three. Repeat lengths are reported with an accuracy of ± 1 repeat.

### iPSC generation and differentiation to cortical neurons

Fibroblasts from an SCA17 patient were cultured in DMEM high glucose medium supplemented with 10% (v/v) FBS (all Gibco®, Thermo Fisher Scientific, Waltham, MA, USA) for up to 10 days in 5% CO_2_ at 37 °C. Fibroblasts were reprogrammed into iPSCs by nucleofection with the episomal plasmids pCXLE-hUL, pCXLE-hSK, and pCXLE-hOCT4. In brief, 1 × 10^5^ cells were nucleofected with 1 µg of each plasmid. After 2 days, FGF2 (PeproTech) was added to the culture medium (DMEM, high glucose with 10% (v/v) FBS) in a concentration of 2 ng/ml. The following day, the culture medium was changed to Essential 8 (E8) medium (Gibco®, Thermo Fisher Scientific, Waltham, MA, USA) supplemented with 100 µM NaB (Sigma-Aldrich). Medium was changed every other day for 3–4 weeks, and afterwards colonies were picked, and iPSCs were differentiated into cortical neurons following an established protocol^75,76^. Briefly, iPSCs were seeded at 3 × 10⁵ cells/cm² on Matrigel (Corning)-coated 6-well plates in E8 medium supplemented with 10 μM Y-27632 (Selleckchem). Neural differentiation was carried out in 3 N medium throughout the differentiation. Until day 10, the medium was supplemented with 10 μM SB431542 (Sigma-Aldrich) and 500 nM LDN-193189 (Sigma-Aldrich), with the addition of 20 ng/ml FGF2 (PeproTech) on day 9. Cells were passaged at day 10 at a 1:3 ratio and expanded in 3 N medium supplemented with 10 μM Y-27632 on the day of passaging and 20 ng/ml FGF-2 until day 11. Heparin (100 μg/ml; Sigma-Aldrich) was added on days 14 and 16. A second passage at a 1:3 ratio was performed on day 20, with optional cryopreservation of neural precursor cells. At day 27, cells were dissociated using Accutase (Sigma-Aldrich) and replated onto poly-L-ornithine (Sigma-Aldrich) and Matrigel-coated plates at a density of 1.5 × 10⁵ cells/cm² (six-well format, for protein isolation) or 3× 10⁴ cells/cm² (24-well format, for immunocytochemistry). The following day, 10 μM PD0325901 (Tocris) and 10 μM DAPT (Sigma-Aldrich) were added, with a medium change after 2 days. From day 32 onwards, cells were maintained in 3 N medium with medium changes every other day until day 37.

### Cell-based calpain assays

Cell-based calpain activation and inhibition assays were performed as previously described^28^. For activation of endogenous calpains, transiently transfected 293T cells and induced cortical neurons (iCNs) were incubated in Opti-MEM® I Reduced Serum Media (Gibco®, Thermo Fisher Scientific) containing 3 µM (for 293T cells) or 1 µM (for iCNs) of ionomycin (in DMSO) (407950, Calbiochem, Merck) and 5 mM CaCl_2_ for 3 h (for 293T cells) or 1 h (for iCNs) at standard culture conditions. For calpain-specificity control, 293T cells were pre-incubated with 25 µM N-Acetyl-L-leucyl-L-leucyl-L-norleucinal (ALLN; in DMSO) (A6185; Sigma-Aldrich, St. Louis, MO, USA), 25 µM calpeptin (n-benzyloxycarbonyl-L-leucylnorieucinal; in DMSO) (C8999, Sigma-Aldrich) or 25 µM calpain inhibitor III (CI-III; carbobenzoxy-valinyl-phenylalaninal; in DMSO) (208722, Calbiochem, Merck), pre-diluted in Opti-MEM® I Reduced Serum Media, 1 h prior to ionomycin/CaCl_2_ administration. For iCNs, 1-h pre-treatment with 20 µM CI-III diluted in Opti-MEM® I Reduced Serum Media served as the calpain-specificity control. For vehicle controls, DMSO was used.

### Cell viability analysis

For the analysis of cell viability in cells expressing different TBP variants, 293T cells were seeded in poly-L-lysine (P4707, Sigma-Aldrich) pre-coated 96-well culture plates (ViewPlate-96 Black, Perkin Elmer, Massachusetts, USA) and transfected 24 h later. After an additional 72 h of incubation, cell viability was assessed using the PrestoBlue assay (Thermo Fisher Scientific, Karlsruhe, Germany), according to the manufacturer’s instructions, with the exception of reading the fluorescence signals after 30 min of incubation with PrestoBlue reagent.

### Immunocytochemistry (ICC) staining and fluorescence microscopy

For immunocytochemistry (ICC) staining of transfected cells and fibroblasts, cells were seeded on poly-L-lysine (P4707, Sigma-Aldrich) pre-coated eight-well chamber slides (80841, Ibidi, Gräfelfing, Germany), transfected after 24 h with respective constructs and returned to standard culture conditions. After 72 h, cells were pre-fixed via supplementation of 0.4% (w/v) paraformaldehyde in 1× DPBS (Gibco®, Thermo Fisher Scientific) (PFA) to the medium and incubation at 37 °C for 10 min. Pre-fixative was discarded, cells were washed briefly with 1× DPBS and fixed with 4% (w/v) PFA at room temperature (RT) for 15 min. For ICC staining of iCNs, cells were grown on coverslips and fixed in 4% (w/v) PFA at RT for 15 min without prior pre-fixation. After washing cells with 1× DPBS, cells were incubated in blocking/permeabilisation buffer (10% (w/v) BSA, 0.5% (v/v) Triton X-100, 0.02% (w/v) NaN_3_ in 1× DPBS) for 1 h at RT. Subsequently, cells were probed with primary antibodies diluted in ICC antibody diluent (1% (w/v) BSA, 0.5% (v/v) Triton X-100, 0.02% (w/v) NaN_3_ in 1× DPBS) at 4 °C overnight. A detailed list of primary antibodies used for ICC stainings can be found in Supplementary Table 2. Afterwards, cells were washed with 1× DPBS and incubated with respective secondary antibodies: goat anti-mouse Alexa Fluor 488, goat anti-rabbit Alexa Fluor 488, goat anti-mouse Alexa Fluor 555, goat anti-mouse Alexa Fluor 594, goat anti-rabbit Alexa Fluor 594, goat anti-rat Alexa Fluor 594, or goat anti-chicken Alexa Fluor 647 (A32723, A27034, A-21424, A32742, A-11037, A-11007, A32933; Thermo Fisher Scientific) diluted 1:1000 for iCN staining, or 1:500 for all other cells, in antibody diluent at RT for 1 h. After washing with 1× DPBS and an additional rinse with ddH_2_O, stained cells were mounted and counterstained with VECTASHIELD® Antifade Mounting Medium with DAPI (H-1200, Vector Laboratories, Newark, CA, USA).

Cells were analysed with an Axioplan 2 fluorescence microscope, equipped with an ApoTome, Plan-Neofluar 40×/0.75 and Plan-Apochromat 63×/1.4 oil objectives, and an AxioCam MRm camera, using the AxioVision 4.8 imaging software (all Zeiss, Oberkochen, Germany).

To acquire confocal images, two confocal microscopes were used. Confocal images for orthogonal cross sections were acquired at a 1000× magnification on an ECLIPSE Ti2 microscope equipped with a CSU-W1 SoRa confocal system and a CFI SR HP Plan Apochromat Lambda S 100×C Sil objective, using NIS-Elements AR 5.42 (all Nikon, Tokyo, Japan). All other confocal images were acquired at a 200× or 630× magnification on a Stellaris 5 microscope, HC PL APO 20x/0.75 IMM CORR CS2 and HC PL APO 63x/1,30 GLYC CORR CS2 objective, with the LAS X 4.8.2.29567 imaging software (all Leica, Wetzlar, Germany).

### Immunohistochemistry staining and fluorescence microscopy of rat tissue

For immunohistochemistry (IHC) staining, paraffin-embedded brains of 10-month-old male wild-type and SCA17 (TBPQ64) rats of the Sprague Dawley strain, obtained from a previous study^41^, were cut into 7 µm sagittal sections and mounted on slides. Deparaffinization and rehydration were performed in a xylene and ethanol series using the Leica autostainer XL (Leica). Antigens were retrieved with a 15 min microwave treatment in 80 mM sodium citrate and 20 mM citric acid pH 6.0, and then washed with 1× PBS. Sections were blocked for 1 h at RT with blocking solution (5% normal goat serum (S-1000, Vector Laboratories), 3% BSA, 0.1% Triton X-100, 0.02% NaN_3_ in 1× DPBS). Slides were incubated with primary antibodies diluted in IHC antibody diluent (2% normal goat serum, 3% BSA, 0.1% Triton X-100, 0.02% NaN_3_) overnight at 4 °C in a humid chamber. A detailed list of used primary antibodies for IHC staining can be found in Supplementary Table 2. Afterwards, sections were washed with 1× PBS and incubated with fluorescence-tagged secondary antibodies goat anti-rabbit Alexa Fluor 555 and goat anti-mouse 647 (A32732, A-21235; Thermo Fisher Scientific) diluted 1:300 in antibody diluent at RT for 1 h. Sections were washed in 1× PBS, rinsed with ddH_2_O, mounted and counterstained in VECTASHIELD® Antifade Mounting Medium with DAPI (H-1200, Vector Laboratories).

To image sections at ×40 and ×100 magnification, an Axioplan 2 fluorescence microscope, equipped with Achroplan 4×/0.10 and Plan-Neofluar 10×/0.30 objectives, and an AxioCam MRm camera, using the AxioVision 4.8 imaging software (all Zeiss) was used.

Fluorescence intensities of stained animal brain sections were assessed using the Fiji software^77^ (version 20240605-1417), and an in-house established Fiji script for semi-automated intensity analysis. Briefly, the mean signal intensities of ×40 magnified images of cerebellar layers (molecular layer, Purkinje cell layer, and granular cell layer) were determined and corrected for local mean background intensities. Details on the Fiji script will be shared upon request.

### Protein extraction

For cell homogenates and lysates, 293T cells were detached by gentle pipetting and collected by centrifugation at 500×*g* for 5 min and 4 °C. Cell pellets were washed with cold 1× DPBS followed by brief centrifugation at 500×*g* for 2.5 min. For cell homogenisation, pellets were resuspended in an appropriate amount of RIPA buffer (50 mM Tris pH 7.5, 150 mM NaCl, 10% glycerol, 0.1% (w/v) SDS, 0.5% sodium deoxycholate and 1% (v/v) Triton X-100) supplemented with cOmplete™ protease inhibitor cocktail (Roche Diagnostics) and PhosSTOP™ phosphatase inhibitor (Roche Diagnostics) and incubated on ice for 30 min. Homogenates were ultrasonicated using a Sonopuls ultrasonic homogeniser (Bandelin electronic, Berlin, Germany) for 3 s and 10% pulse duration at 10% power, whereas cell lysates were obtained by centrifugation of protein extracts for 30 min at 4 °C and 20,000×*g*.

Protein extracts from the cerebella of 10-month-old male wild-type and SCA17 (TBPQ64^41^) rats of the Sprague Dawley strain were generated in a previous study, which provides detailed information on animal maintenance and tissue sampling^28^.

Protein concentrations of homogenates and lysates were determined by the Bradford assay (Bio-Rad Protein Assay; Bio-Rad Laboratories, Basel, Switzerland) and measured spectrophotometrically in a BioTek Synergy HT microtiter plate reader using the BioTek Gen5 software (both Agilent Technologies, Inc., Santa Clara, CA, USA). Samples were stored at −80 °C until further analysis.

### Cytoplasmic-nuclear fractionation

To separate cytoplasmic and nuclear proteins, the *Rapid, Efficient And Practical* (REAP) fractionation method, based on the original protocol by Suzuki et al^78^., was applied, with following adaptations: Briefly, cell pellets were resuspended in TBS-I lysis buffer (50 mM Tris pH 7.5, 150 mM NaCl, 0.1% (v/v) IGEPAL CA-630) supplemented with cOmplete™ protease inhibitor cocktail EDTA-free (Roche Diagnostics), PhosSTOP™ phosphatase inhibitor (Roche Diagnostics), and 10 µM calpeptin (C8999, Sigma-Aldrich). To receive a total protein (whole cell) homogenate, an aliquot of one-fourth of the lysis volume was immediately transferred to a new tube and diluted with an equal volume of TBS-I lysis buffer. The remaining material was centrifuged at 20,800×*g* for 10 s. The supernatant, containing the cytoplasmic fraction, was carefully transferred to a separate tube, while avoiding the disruption of the nuclear pellet. The nuclear fraction was washed by resuspending the pellet with double the initial lysis volume in TBS-I lysis buffer, centrifuged at 20,800×*g* for 10 s, and discarding the supernatant. This step was repeated once. The washed nuclear fraction was resuspended in half of the initial lysis volume in TBS-I lysis buffer. Total protein homogenate and nuclear fraction were ultrasonicated at a 10% pulse duration and 10% power for 3 s using a Sonopuls ultrasonic homogeniser (Bandelin electronic). The nuclear fraction underwent up to two additional ultrasonication steps to effectively disrupt nuclear membranes. Protein concentrations were determined by Bradford assay and measured spectrophotometrically in a microtiter plate.

### Immunoprecipitation and on-bead calpain cleavage assay

Immunoprecipitation (IP) of target proteins was performed using ChromoTek Myc-Trap® Agarose (ChromoTek GmbH, Planegg, Germany) according to the manufacturer’s protocol with the following adaptations. Shortly, cell lysates were prepared by resuspending cell pellets in TRAP lysis buffer (10 mM Tris pH 7.5, 150 mM NaCl, 0.5 mM EDTA, 0.5% IGEPAL CA-630) containing cOmplete™ protease inhibitor cocktail EDTA-free and PhosSTOP™ phosphatase inhibitor (Roche Diagnostics), ultrasonication for 3 s and 10% pulse duration at 10% power using a Sonopuls ultrasonic homogeniser (Bandelin electronic) and centrifugation for 30 min at 4 °C and 20,000×*g*. Cell and tissue lysates were diluted to 5 µg/µl protein with TRAP lysis buffer after protein concentrations were determined by Bradford assay, and TRAP dilution buffer (10 mM Tris/Cl pH 7.5, 150 mM NaCl, 0.5 mM EDTA, containing cOmplete™ protease inhibitor cocktail EDTA-free and PhosSTOP™ phosphatase inhibitor (Roche Diagnostics)) was added in a ratio of 2:3 to the samples. For IP, 500 µg or 1000 µg protein in a maximum volume of 500 µl was added to the equilibrated beads and incubated overnight at 4 °C using an end-over-end rotator. Beads were washed thrice using TRAP dilution buffer, and proteins were eluted with 2× LDS buffer (2× LDS, 100 mM dithiotreitol (DTT)) under heat-denaturation for 10 min at 70 °C.

In case of an on-bead calpain cleavage assay, beads were resuspended in 100 µl calpain reaction buffer (CRB; 20 mM HEPES/KOH pH 7.6, 10 mM KCl, 1.5 mM MgCl_2_, 1 mM DTT) after the last washing step. 200 mM CaCl_2_ and 200 ng of recombinant calpain-1 (208712, Merck) were added and incubated for 10 min on an end-over-end rotator at RT. The time series of calpain-1 cleavage was carried out for 0, 1, and 5 min. The reaction was stopped by adding 4× LDS sample buffer (1 M Tris pH 8.5, 50% (v/v) glycerol, 8% (w/v) LDS, 2 mM EDTA, 0.1% (w/v) Orange G) in a ratio 3:1 and 100 mM DTT, followed by heat-denaturation for 10 min at 70 °C. Beads were sedimented by centrifugation for 5 min at maximum speed and 4 °C. Supernatant was transferred into new tubes and stored at -80 °C until further analysis.

### Western blotting

Western blotting was performed according to standard procedures. Briefly, 25 µg of protein from cell homogenates or lysates were supplemented with 4× LDS sample buffer in a ratio of 3:1 and 100 mM DTT, followed by heat-denaturation for 10 min at 70 °C. Electrophoretic separation of protein samples was achieved via SDS-polyacrylamide gel electrophoresis (SDS-PAGE) using self-made 9% Bis-Tris gels and MOPS electrophoresis buffer (50 mM MOPS, 50 mM Tris pH 7.7, 0.1% (w/v) SDS, 1 mM EDTA). Proteins were blotted on Amersham™ Protran™ Premium 0.2 µm nitrocellulose membrane (Cytiva, Freiburg, Germany) using Bicine/Bis-Tris transfer buffer (25 mM Bicine, 25 mM Bis-Tris pH 7.2, 1 mM EDTA, 15% (v/v) methanol) in a TE22 Transfer Tank (Hoefer, Inc., Holliston, MA, US) at 80 V and a maximum of 250 mA for 2 h followed by standard immunodetection protocol.

### Filter retardation assay

Detection of SDS-insoluble TBP was performed as previously described^28^. Briefly, 3 µg total protein of cell homogenates was diluted in 1× DPBS containing 2% (w/v) SDS and 50 mM DTT, and heat-denatured for 5 min at 95 °C. After equilibrating Amersham™ Protran™ 0.45 µm nitrocellulose membrane (GE Healthcare) with 1× DPBS containing 0.1% (w/v) SDS, samples were filtered through the membrane using a Minifold® II Slot Blot System (Schleicher & Schuell). Slots were washed with 1× DPBS containing 0.1% SDS (w/v) and 1× DPBS before the standard immunodetection protocol was applied.

### Membrane-based immunodetection

Membranes were blocked with 5% (w/v) skim milk powder (Sigma-Aldrich) in TBS (10 mM Tris pH 7.5, 150 mM NaCl) for 1 h at RT, and incubated with primary antibodies diluted in 1× TBS-T (1× TBS with 0.1% (v/v) Tween-20) overnight at 4 °C. A detailed list of primary antibodies used for membrane-based immunodetection can be found in Supplementary Table 3. Afterwards, membranes were washed with 1× TBS-T and probed with respective secondary IRDye® antibodies goat anti-mouse 680LT (P/N 926-68020), goat anti-mouse 800CW (P/N 926-32210), and goat anti-rabbit 800CW (P/N 926-32211) (all 1:5,000; LI-COR Biosciences) or horseradish peroxidase (HRP)-linked antibodies goat anti-mouse HRP (#A28177; Thermo Fisher Scientific), donkey anti-mouse HRP (715-035-150; Jackson ImmunoResearch Europe Ltd, Cambridgeshire, UK), and goat anti-rabbit HRP (#G-21234; Thermo Fisher Scientific) at RT for 1 h. After secondary antibody incubation, membranes were washed with 1× TBS-T. For chemiluminescence detections, membranes were shortly incubated with 1× TBS followed by incubation with WesternBright^TM^ Sirius HRP substrates (K-12043; Advansta Inc., San Jose, CA, USA) for 2 min at RT. Fluorescence and chemiluminescence signals were detected using the LI-COR ODYSSEY® FC and quantified with Image Studio 6.1.0.79 software (both LI-COR Biosciences).

### Membrane-based inclusion body microscopy

For membrane-based imaging of TBP inclusion bodies (IBs), a filter retardation assay was performed as described above. After primary antibody incubation, secondary antibodies goat anti-mouse Alexa Fluor 488 and rabbit Alexa Fluor 594 (all 1:500; A32723, A-11037, both Thermo Fisher Scientific) were added and incubated at RT for 1 h. Membranes were mounted on glass slides and fluorescent images were taken at a ×40 magnification on an Axioplan 2 imaging microscope equipped with an Achroplan 4×/0.10 objective and an AxioCam MRm camera, using the AxioVision 4.8 imaging software (all Zeiss).

### Microscopy-based assessment of inclusion bodies

To quantify the frequency of staining-positive particles imaged by membrane-based inclusion body microscopy, an in-house-developed Fiji macro was used, as previously described^79^. For each slot, three images were acquired and analysed. To reduce false positive events, particles with intensity densities below the 75% percentile of respective mock conditions were excluded from further analyses.

Using the same Fiji macro, inclusion body (IB) frequency, cross-sectional area, equivalent circular area diameter (d_ECA_), and roundness were determined in cells stained by ICC.

Orthogonal slices and maximum intensity projections of confocal z-stacks were generated using Fiji^77^, and three-dimensional reconstructions were created using the built-in tools of Leica LAS X software.

Microscopic evaluation of TBP and AMOT co-aggregation was performed manually by assessing the fluorescence signal intensity of both immunostainings.

### Mass spectrometry and data processing

Protein extracts from 293T cell or rat cerebellum samples for label-free quantitative (LFQ) proteomics or immunoprecipitated calpain-dependent protein fragments for calpain cleavage site analysis were purified using Bis-Tris-based SDS-PAGE. Coomassie-stained gel pieces were excised, and in-gel digested using trypsin (for protein extracts) or chymotrypsin (for purified protein fragments), as described previously^80^. After desalting using C18 Stage tips^81^ extracted peptides were separated on an Easy-nLC 1200 system coupled to a Q Exactive HF mass spectrometer (both Thermo Fisher Scientific) as described elsewhere^82^ with slight modifications: The peptide mixtures were separated using a 1-h gradient, and the 7 most intense precursor ions were sequentially fragmented in each scan cycle using higher energy collisional dissociation (HCD) fragmentation.

Acquired MS spectra were processed with the MaxQuant software package version 2.2.0.0^83^ with integrated Andromeda search engine^84^. For 293T cell analysis, a database search was performed against a target-decoy *Homo sapiens* database obtained from UniProt, containing 96,817 protein entries and 286 commonly observed contaminants. For analysis of the proteome from rat cerebella, a database search was performed against a *Rattus norvegicus* database obtained from UniProt containing 49,592 protein entries and 286 commonly observed contaminants. In addition, one small database was created containing sequences used for pulldown. For calpain cleavage site identification, endoprotease chymotrypsin was defined as a protease with a maximum of two missed cleavages; the enzyme mode was semi-specific. Oxidation of methionine and N-terminal acetylation were specified as variable modifications, whereas carbamidomethylation on cysteine was set as a fixed modification. Initial maximum allowed mass tolerance was set to 4.5 parts per million (ppm) for precursor ions and 20 ppm for fragment ions. Peptide, protein and modification site identifications were reported at a false discovery rate (FDR) of 0.01, estimated by the target/decoy approach^85^. The LFQ (Label-Free Quantification) algorithm was enabled, and a match between runs^86,87^ and LFQ protein intensities was used for relative protein quantification of three replicates. Downstream bioinformatic analysis (two-sample *t* tests and Volcano plots) was performed using the Perseus software package, version 1.6.2.3. Data were filtered for contaminants, reversed and only identified by site entries.

### RNA extraction

RNA from 293T cells was isolated using the RNeasy Mini Kit (QIAGEN) with minor modifications. After disrupting cells in an appropriate volume of RLT buffer, cells were homogenised using QIAshredder spin columns. DNA digestion was conducted using QIAGEN’s RNase-free DNase Set according to the specifications of the manufacturer’s protocol. The RNeasy spin column was dried by centrifugation at full speed for 3 min. RNA was eluted after an incubation of 5 min at RT in 30 µl RNase-free water.

Total RNA of zebrafish embryos was extracted using Zymo’s RNA Microprep kit (Zymo Research Europe GmbH, Freiburg, Germany), which includes a DNaseI step to remove genomic DNA.

### RNA sequencing and data processing

The concentration of RNA was measured using the Qubit Fluorometric Quantitation and RNA Broad-Range Assay (Thermo Fisher Scientific). RNA Integrity Number RIN was determined using the Fragment Analyzer 5300 and the Fragment Analyzer RNA kit (Agilent Technologies) and presented a good integrity (RIN > 9.5). For library preparation, the mRNA fraction was enriched using polyA capture from 100 ng of total RNA using the NEBNext Poly(A) mRNA Magnetic Isolation Module (New England Biolabs GmbH, Frankfurt am Main, Germany). Subsequently, libraries were prepared using the NEBNext Ultra II Directional RNA Library Prep Kit for Illumina and NEBNext UDI UMI (New England Biolabs GmbH) following the manufacturer’s instructions. To minimise technical batch effects, library preparations were performed using the liquid handler Biomek i7 (Beckman Coulter GmbH, Krefeld, Germany). The library molarity was determined by measuring the library size (approximately 400 bp) using the Fragment Analyzer 5300 and the Fragment Analyzer DNA HS NGS fragment kit (Agilent Technologies), and the library concentration ( > 2 ng/µl) using Qubit Fluorometric Quantitation and dsDNA High sensitivity assay (Thermo Fisher Scientific). The libraries were denatured according to the manufacturer’s instructions, diluted to 150 pM and sequenced as paired-end 100 bp reads on an Illumina NovaSeq 6000 (Illumina, Inc., San Diego, CA, USA). The sequencing aimed to achieve a depth of approximately >25 million clusters per sample.

Read quality of RNA-seq data in fastq files was assessed using ngs-bits (2023_11-197-g1bcb6874) to identify sequencing cycles with low average quality, adaptor contamination, or repetitive sequences from PCR amplification. Reads were aligned using STAR v2.7.10b^88^ to the GRCh38, and alignment quality was analysed using ngs-bits and visually inspected in the Integrative Genome Viewer (v2.15.4). Normalised read counts for all genes were obtained using Subread (v2.0.4) and edgeR (v3.42.4).

Raw expression values are available for 61,850 genes in 15 samples. Raw gene expression was filtered by demanding a minimum expression value of 1 cpm (counts per million) in at least 2 samples. Filtered data contains expression values for 16,340 genes. The distribution of logarithmised cpm-normalised expression values shows similar characteristics over all samples. Based on the filtered dataset, samples were investigated with respect to their pairwise similarity. Spearman’s rank correlation coefficient was calculated for each pair of samples. A hierarchical clustering was performed on the resulting similarity values. Differential gene expression analysis was conducted based on the filtered gene expression dataset. A statistical model incorporating the group property of samples was tested by fitting a negative binomial distribution using a generalised linear model (GLM) approach. For each gene, gene expression fold changes (log_2_ fold change) were computed, and a statistical test was performed to assess the significance, which is given as raw *P* value and adjusted *P* value (FDR).

### Zebrafish maintenance and manipulation

Zebrafish were maintained in a circulating water system (Tecniplast Germany GmbH, Hohenpeißenberg, Germany) with automated monitoring and adjustment of water parameters (conductivity, pH, and temperature). Zebrafish were kept under a dark–light cycle (10 h dark/14 h light) and fed four times a day. In the morning, fish received freshly-hatched artemia and afterwards dry food (Zebrafeed by Sparos, Olhão, Portugal) dispensed by the TRITONE automatic feeding system (Tecniplast Germany GmbH). Lines used included AB and EK as wild-type zebrafish and the reporter line tg(isl1:gfp)^89^, expressing GFP in motor neurons. To generate *amot* loss-of-function embryos using Crispr/Cas9, a ribonucleoprotein complex consisting of two guideRNAs and Cas9 HiFi (both Integrated DNA Technologies, Inc., Coralville, IA, USA) was assembled and injected at the 1-cell stage. The following sequences in exon 2 of *amot* were targeted: *Amot* gRNA1: 5’-AAGCTCCTCCTTCCATGAGG, *Amot* gRNA2: 5’-GACTGGAGTCACCAACTCTA. As control, embryos were injected with Cas9 alone or with a complex consisting of Cas9 protein and a negative control gRNA (IDT, Leuven, Belgium). Rescue experiments were achieved by subsequent injections of ribonucleoprotein complex and capped RNA. Gene editing in every individual embryo was verified by PCR amplification of exon 2 before further analysis. In addition, the editing capacity of the gRNAs was assessed by Sanger sequencing (Microsynth AG, Göttingen, Germany) of exon 2 PCRs in edited and control-injected embryos. To knock down *amot*, a previously published MO was used^45^. MO injections were controlled against embryos injected with a standard control morpholino oligonucleotide (MO) (Gene Tools, LLC, Philomath, OR, USA). In addition, in all experiments, embryos were compared to non-injected controls to assess clutch quality. Rescue experiments with human AMOT as well as overexpression of TBP variants were achieved by injection of capped RNA at the 1-cell stage. Capped RNA was generated from linearised plasmids, which were then transcribed using the mMESSAGE mMACHINE SP6 transcription kit (Ambion, Thermo Fisher Scientific). As a template for human *AMOT* a pcDNA3-HA AMOT overexpression plasmid was used, which was a gift from Kunliang Guan (Addgene plasmid # 32821; http://n2t.net/addgene:32821; RRID:Addgene 32821)^90^. After manipulation, all embryos were allowed to develop at 28.5 °C until the desired stage.

### In situ hybridisation

A 1095 bp fragment of zebrafish *amot* (Ensemble accession number ENSDART00000141096.2) was amplified from cDNA generated from 24 hpf zebrafish embryos and cloned via TOPO TA cloning (Invitrogen, Thermo Fisher Scientific) into pCRII. A DIG-labelled in situ probe was generated after linearization of the plasmid with XhoI (New England Biolabs GmbH) and in vitro transcription using SP6 RNA polymerase and the DIG labelling mix (Roche Diagnostics). To generate a probe against the *dopamine transporter* (*dat*, *slc6a3*, Genbank accession no. NM_131755.1), a 1256 bp fragment was amplified from 24 hpf cDNA. A T3 polymerase binding element was fused to the reverse primer and a probe transcribed using T3 polymerase (Promega GmbH, Walldorf, Germany). In situ hybridisation was done according to standard protocols^91^ using embryos at the indicated stages.

### Movement analyses of zebrafish embryos

To investigate spontaneous coiling and tailflick movements, 5-min movies were recorded of 27 hpf embryos still in their chorion using a Leica S9i stereomicroscope (Leica Microsystems GmbH, Wetzlar, Germany) with an integrated camera. Spontaneous bursts of activity were counted manually.

The swimming ability of zebrafish embryos was assessed at 48 hpf after manual dechorionisation using forceps. Embryos were gently tapped on the back of their head, and their reaction was recorded and scored based on the following: no reaction (0), twitch upon touch (1), swimming behaviour upon touch (2). All embryos were tapped at least twice and up to five times.

### Immunofluorescence in zebrafish embryos

To allow for the detection of not only cell bodies, but also the axons extended from motor neurons, the expressed GFP of the transgenic fish line was enhanced by an additional anti-GFP staining. Similarly, human TBP overexpression was detected by immunofluorescence as follows: Embryos at the desired stage were fixed in 4% buffered paraformaldehyde for 1 h. After several washes in PBS-T (1× PBS with 0.1% Tween-20), the embryos were incubated in distilled water for 5 min, permeabilised in ice-cold acetone for 7 min and again incubated for 5 min in water. Following another 5-min incubation step in PBS-T with 1% DMSO (PBD-T), the embryos were blocked for at least 2 h in 10% normal goat serum (Biozol Diagnostica Vertrieb GmbH, Hamburg, Germany) diluted in PBD-T. Primary and secondary antibodies were diluted in blocking buffer. Embryos were first incubated overnight at 4 °C in the primary antibody solution. After several washing steps in PBD-T containing 1% normal goat serum and 0.1 M NaCl, the embryos were incubated for several hours at RT in secondary antibody solution, followed by several final washes. For confocal imaging, the embryo’s tail was cut off and mounted between two coverslips in VECTASHIELD® Antifade Mounting Medium with DAPI (Vector Laboratories). Antibodies used were: mouse anti-GFP (1:250; clone 3E6, A-11120, Thermo Fisher Scientific), mouse anti-TBP (clone 1TBP18, sc-56794, Santa Cruz Biotechnology), goat anti-mouse Alexa Fluor 488 (1:1,000, #4408, Cell Signaling Technology), donkey anti-mouse Alexa Fluor 568 (1:1,000; A10037; Thermo Fisher Scientific).

### Imaging of zebrafish embryos

Zebrafish embryos processed by in situ hybridisation were imaged on a Leica M125 stereomicroscope and a Flexacam C1 colour camera (both Leica Microsystems GmbH). Fluorescence images of immunostained embryos were acquired with a M205 FCA stereomicroscope and a K8 sCMOS camera (both Leica Microsystems GmbH). Z-stacks of immunostained zebrafish embryos were acquired with a Leica Stellaris 5 confocal microscope (Leica Microsystems GmbH) equipped with a system-optimised section distance. Cell bodies of motor neurons were counted with the help of the Cell Counter plug-in in Fiji^77^.

### Quantitative real-time PCR

To analyse gene expression in 293T cells, 1 µg of RNA was reverse transcribed using the QuantiTect Reverse Transcription Kit (Qiagen) according to the manufacturer’s protocol. Relative gene expression of *TBP* and *AMOT* was analysed by quantitative real-time PCR (qPCR) using the SYBR Green PCR Master Mix (Qiagen). In short, 1× QuantiTect SYBR Green PCR Master Mix, 1 µM of forward and 1 µM of reverse primer (metabion international AG), as well as RNase-free water were added to cDNA. The qPCR was run on the LightCycler 480 II (Roche Diagnostics) using the LightCycler 480 Software 1.5.62 SP3 following the manufacturer’s protocol. For normalisation, reference genes *SDHA* and *YWHAZ* were employed. The following primers were used: *TBP*: forward: 5’-GAAGTTGGGTTTTCCAGCTAA, reverse: 5’-GGAGAACAATTCTGGGTTTGA; *AMOT*: forward: 5’-GGGCATGTCCGTTCCTTGAG, reverse: 5’-AGGTGGATGGGCCTTAACTC; *SDHA*: forward: 5’-AGAAGCCCTTTGAGGAGCA, reverse: 5’-CGATTACGGGTCTATATTCCAGA; *YWHAZ*: forward: 5’-TCCAGGGACAGAGTCTCAGC, reverse: 5’-TCCATGACTGGATGTTCTGTG.

To analyse gene expression in zebrafish embryos, equal amounts of RNA were transcribed into cDNA using the Protoscript II Kit (New England Biolabs GmbH). qPCR was done on a Quantstudio^TM^ 3 Real-Time PCR System (Thermo Fisher Scientific) with Luna® Universal qPCR Master Mix (New England Biolab GmbH) and the Universal Probe system (Roche Diagnostics). All reactions were done in triplicate and normalised to *beta 2-microglobulin* (*b2m*). The following primers and probes were used: *b2m* (UP65): forward: 5’- ACATCACTGTACAGGGGAAAGTC-3’, reverse: 5’-TCCGTTCTTCAGCAGTTCAA-3’; *amot* (UP23): forward: 5’-AAGAGCCAGGAGAACAGCAAA-3’, reverse: 5’-TACTGCTGTGAAACACTGGCA-3’; *ctgf* (UP5): forward: 5’-CCACAAGAAGACACCTTCCAG-3’, reverse: 5’-CAGGCTGGAGACCGTGAG-3’; *cyr61* (UP68): forward: 5’-TTGCTCTGCTGACGG AG-3’, reverse: 5’-GGGCAGGATGGAGGAGAC-3’.

### In silico calpain cleavage site prediction

For in silico prediction of calpain cleavage site in human TBP (UniProt identifier: P20226-1), the Group-based Prediction System–Calpain Cleavage Detector (GPS-CCD v1.0) tool was employed (available at http://ccd.biocuckoo.org)^92^. Resulting cleavage likelihood scores for every amino acid position were normalised to the maximum threshold value (0.654) and graphically visualised using the GraphPad Prism software version 10.6.1 (GraphPad Software, Dotmatics, Boston, MA, USA).

### Pathway analysis

For canonical pathway enrichment, a core analysis for differentially expressed genes (DEGs) obtained from RNA sequencing was performed using QIAGEN Ingenuity Pathway Analysis (IPA) software (version 24.0.2). Suggested default parameters and DEGs with *P* value  ≤  0.05 were used for analysis. Results were graphically visualised by GraphPad Prism software version 10.6.1 (GraphPad Software).

Additional in silico pathway and protein-protein interaction analyses were performed using the Search Tool for the Retrieval of Interacting Genes/Proteins (STRING) version 12.0 (https://string-db.org/)^93^. Networks were adjusted using CorelDRAW Graphics Suite 2024.

### Cell-type enrichment analysis using PanglaoDB

To infer the cell type from which the proteomics signatures were obtained, potential cell-type enrichment analysis was performed using the PanglaoDB Augmented 2021 database^94^. The PanglaoDB dataset was downloaded from EnrichR libraries (https://maayanlab.cloud/Enrichr/#libraries) and processed to obtain cell-type-specific marker genes. Each cell type was represented as a set of curated marker genes.

For the analysis, the decoupleR-based over-representation analysis (ORA)) was performed using the decoupleR (v2.16.0) R package^95^. The PanglaoDB data were converted to network format, with cell types as the source and marker genes as the targets. The rat gene symbols were converted to uppercase for the correct mapping with PanglaoDB.

The proteomics data were obtained from mass spectrometry-based experiments and consisted of significantly altered proteins (173) and total detected proteins (1830). The total detected proteins were used as the background universe, and the significant proteins were used as the foreground (hits).

To achieve this, a binary matrix was created, where each gene in the background was given a value of 1 if it was found in the significant gene set, and 0 if it was not. The run_ora function in the decoupleR package was used for the over-representation analysis, which performs Fisher’s exact test to determine the enrichment of cell-type marker genes in the input gene list. However, only gene sets with at least 5 genes were considered for the analysis. The adjusted *P* values were determined using the Benjamini & Hochberg procedure for correcting the false discovery rate.

An independent analysis was also performed using the enricher function in the clusterProfiler package, which uses the hypergeometric test^96^. Although the analysis was performed on the PanglaoDB gene sets, the background was not specified, making it comparable to the Enrichr platform^97^. The aforementioned approaches were used in an overlapping manner to identify cell-type signatures in the proteomics data.

### InteractiVenn

For comparative analysis, data obtained from multi-omics approaches were graphically visualised using the web-based InteractiVenn platform^98^.

### Bubble plot

Data obtained from QIAGEN IPA was graphically visualised by bubble plots that were generated using the ggplot2 package^99^ in R 4.2.2^100^. Full script can be provided upon request.

### Statistics and reproducibility

For qualitative analyses, all experiments were independently repeated at least three times and yielded comparable results, ensuring validity and reproducibility. Quantitative data were statistically analysed using GraphPad Prism software version 10.6.1 (GraphPad Software). For in cellulo- and rat tissue sample-based data sets, normal distribution was assessed using the Shapiro–Wilk test. Data Statistical outliers were identified by the ROUT test (*Q* = 1%). Normally distributed data are presented as means + standard error of the mean (s.e.m.). Non-normally distributed data are shown as median, indicating the 25th and 75th percentiles. Two-sided one-sample *t* test, Student’s *t* test, one-way ANOVA, or Kruskal–Wallis test with an adequate post hoc analysis were applied for statistical testing.

Zebrafish data were first assessed for normal distribution using a Shapiro–Wilk test, before the respective test was applied. All *t* test-based analyses as well as Fisher’s exact tests were two-sided.

Significance was assumed with a *P* value ≤ 0.05. For further details, see the respective figure and figure legends, and the Source Data file.

### Reporting summary

Further information on research design is available in the Nature Portfolio Reporting Summary linked to this article.

## Supplementary information

Supplementary Information

Description of Additional Supplementary Information

Supplementary Data 1

Supplementary Data 2

Supplementary Data 3

Supplementary Data 4

Supplementary Software

Reporting Summary

Transparent Peer Review file

## Source data

Source Data

## Data Availability

All data required to support the findings of this study are available within the article, the supplementary information, or are available in publicly available data repositories. The RNA sequencing data generated in this study have been deposited in the NCBI Gene Expression Omnibus (GEO) database under accession code GSE346419. The mass spectrometry proteomics data generated in this study have been deposited in the ProteomeXchange Consortium via the PRIDE^101^ partner repository under accession code PXD083710. Additional information can be provided upon request. Source data are provided with this paper.

## Code availability

Standard codes and software tools, as listed in the “Methods” section, were used in this study. Custom Fiji macros developed for the analysis of microscopy images are provided as Supplementary Software and have been deposited at Zenodo (https://doi.org/10.5281/zenodo.22231266).
