# Supplementary material for: Calpain-mediated TBP cleavage disturbs neuronal AMOT-YAP1 signalling
Source: Nat Commun. 2026 Nov 9;17:11531. doi: 10.1038/s41467-026-79055-x (PMC13748621; doi:10.1038/s41467-026-79055-x)
Supplement: Supplementary file 1 [file 41467_2026_79055_MOESM1_ESM.pdf]

Supplementary Information

Calpain-mediated TBP cleavage disturbs neuronal AMOT-YAP1 signalling

Rana Dilara Incebacak Eltemur[1,2,*], Florian Fath[1,2,3,*], Chrisovalantou Huridou[1,2], Max Duong Phu[4], Till Ivo Stoltenow[5,6], Jacob Helm[5,6], Priscila Pereira Sena[2], Nicolas Casadei[2,7], Ana Velic[8], Roshan Priyarangana Perera[2], Boris Macek[8], Olaf Riess[2], Stefan Hauser[5,6], Huu Phuc Nguyen[1], Melanie Philipp[4], Jonasz Jeremiasz Weber[1,2,**]

[1] Department of Human Genetics, Ruhr University Bochum, 44801 Bochum, Germany

[2] Institute of Medical Genetics and Applied Genomics, University of Tübingen, 72076 Tübingen, Germany

[3] Department of Neurology, Huntington-Center NRW, St. Josef-Hospital Bochum, Ruhr University Bochum, 44791 Bochum, Germany

[4] Department of Experimental and Clinical Pharmacology and Pharmacogenomics, Section of Pharmacogenomics, University of Tübingen, 72074 Tübingen, Germany

[5] German Center for Neurodegenerative Diseases (DZNE), 72076 Tübingen, Germany

[6] Hertie Institute for Clinical Brain Research, University of Tübingen, 72076 Tübingen, Germany

[7] NGS Competence Center Tübingen, University of Tübingen, 72076 Tübingen, Germany

[8] Proteome Center Tübingen, University of Tübingen, 72076, Tübingen, Germany.

***These authors contributed equally.**

****Correspondence should be addressed to:**

Dr. Jonasz Jeremiasz Weber

Department of Human Genetics, Ruhr University Bochum, Universitätsstraße 150, 44801 Bochum, Germany

Phone: +49 7071 29 72257

Email: jonasz.weber@med.uni-tuebingen.de

Supplementary Figures and Supplementary Figure Legends

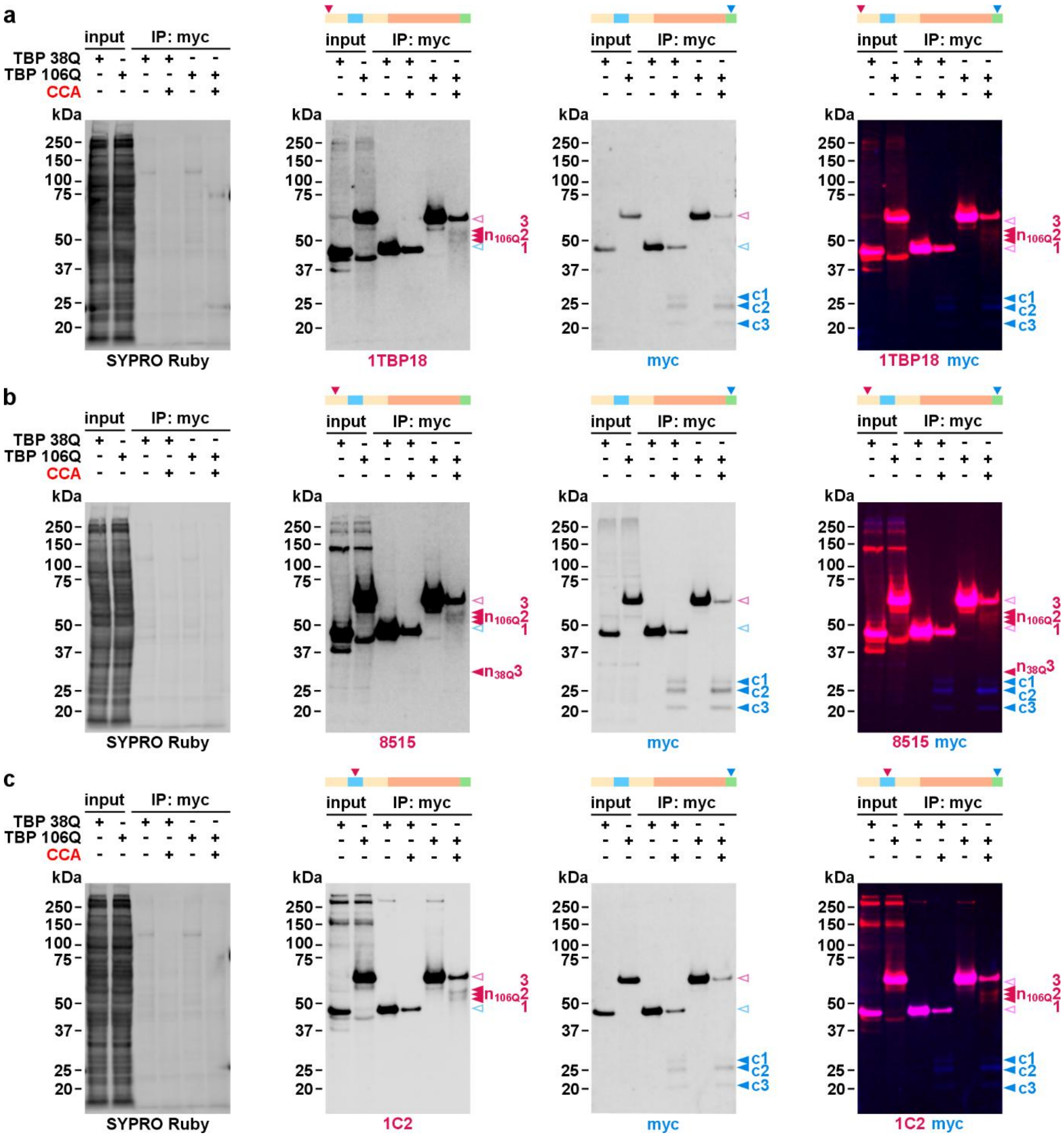

Supplementary Fig. 1: Antibody-based mapping of calpain-1 cleavage-derived TBP fragments. a-c, Western blot analysis of TBP fragmentation after immunoprecipitation (IP) and followed by a calpain-cleavage assay (CCA) with calpain-1. Membranes were probed with a myc-tag-specific antibody combined with TBP-specific antibodies targeting the N-terminus (1TBP18 in **a** or 8515 in **b**) or with a polyQ-specific antibody (1C2, in **c**). SYPRO Ruby staining served as loading control and for validation of IP purity. Full protein extracts from TBP-transfected 293T cells (input) were loaded along the IP samples. Light blue- and pink-rimmed arrowheads mark full-length TBP 38Q and 106Q, respectively. Red and blue arrowheads indicate N- and C-terminal (n1 - 3; c1 - 3) TBP fragments, respectively. The approximate

binding sites of the antibodies used are illustrated above the corresponding blots. Source data are provided as a Source Data file.

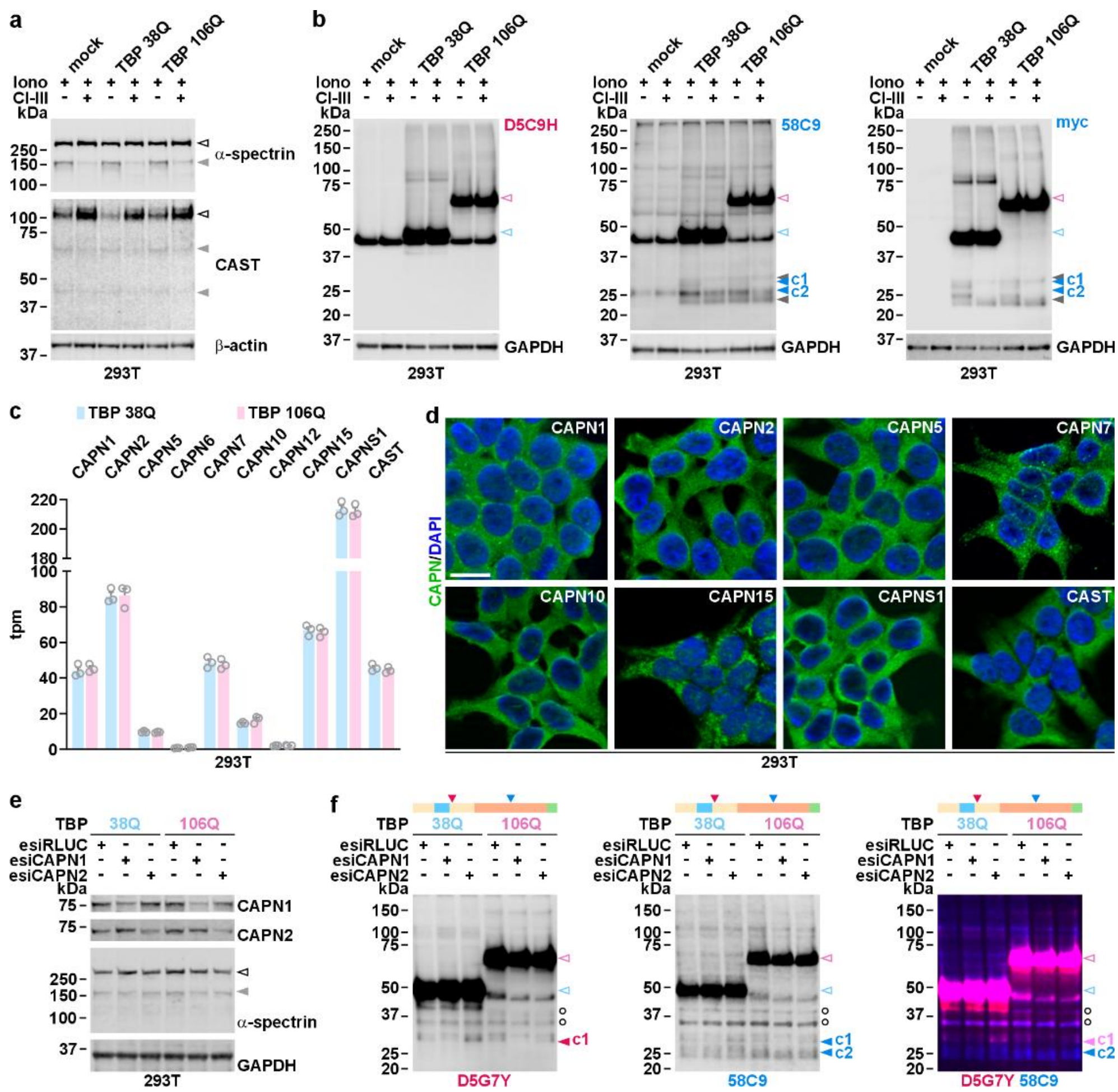

Supplementary Fig. 2: Cell-based calpain-dependent fragmentation of TBP via ionomycin induction. a, 293T cells expressing TBP 38Q or 106Q, as well as mock-transfected control cells, were treated with ionomycin (Iono) to activate calpains and their protein extracts were analysed by western blotting. Calpain activation was confirmed by detecting cleavage of substrates, α-spectrin and calpastatin (CAST). A calpain inhibitor III (CI-III) treatment condition served as a specificity control. β-actin was used as the loading control. Black-rimmed arrowheads indicate full-length α-spectrin and CAST, while grey arrowheads show their respective breakdown products. **b,** Endogenous and overexpressed TBP was detected using an N-terminally binding TBP antibody (D5C9H). An antibody specific for the myc tag was used for detecting the C-terminus of overexpressed TBP. β-actin and GAPDH were used as loading controls. Light blue- and pink-rimmed arrowheads mark full-length TBP 38Q and 106Q, respectively. Blue arrowheads indicate C-terminal fragments (c1 and c2), while grey arrowheads show calpain-independent TBP fragments. **c,** Expression levels of *CAPN1*, *CAPN2*, *CAPN5*, *CAPN6*, *CAPN7*, *CAPN10*, *CAPN12*, *CAPN15*, *CAPNS1*, and *CAST* in 293T cells expressing TBP 38Q (light blue) and TBP 106Q (light pink) in transcripts per million (tpm).

Bars represent means + s.e.m. $n = 3$ biological replicates. **d,** Immunofluorescence staining and microscopy of 293T cells to analyse subcellular localization of CAPN1, CAPN2, CAPN5, CAPN7, CAPN10, CAPN15, CAPNS1, and CAST (all in green). DAPI was used as a nuclear counterstain (blue). Scale bar: 20 µm. **e,** 293T cells expressing TBP 38Q and 106Q co-transfected with esiRNA directed against *CAPN1*, or *CAPN2*. Protein extracts were analysed by western blotting. *Renilla luciferase* targeting esiRNA (esiRLUC) was used as mock control. *CAPN1* and *CAPN2* knockdown and its effect on the CAPN system were confirmed by antibodies against CAPN1, CAPN2, and the calpain substrate α-spectrin. GAPDH served as loading control. **f,** TBP fragmentation was analysed with TBP-specific antibodies targeting an epitope around amino acid residue A110 (D5G7Y, pink) and the C-terminus (58C9, blue). Light blue- and pink-rimmed arrowheads mark full-length TBP 38Q and 106Q, respectively. Pink and blue arrowheads indicate C-terminal fragments c1 and c2. Black-rimmed circles indicate unspecific bands. Illustration of the approximate binding sites of the antibodies are seen above the corresponding blots. Source data are provided as a Source Data file.

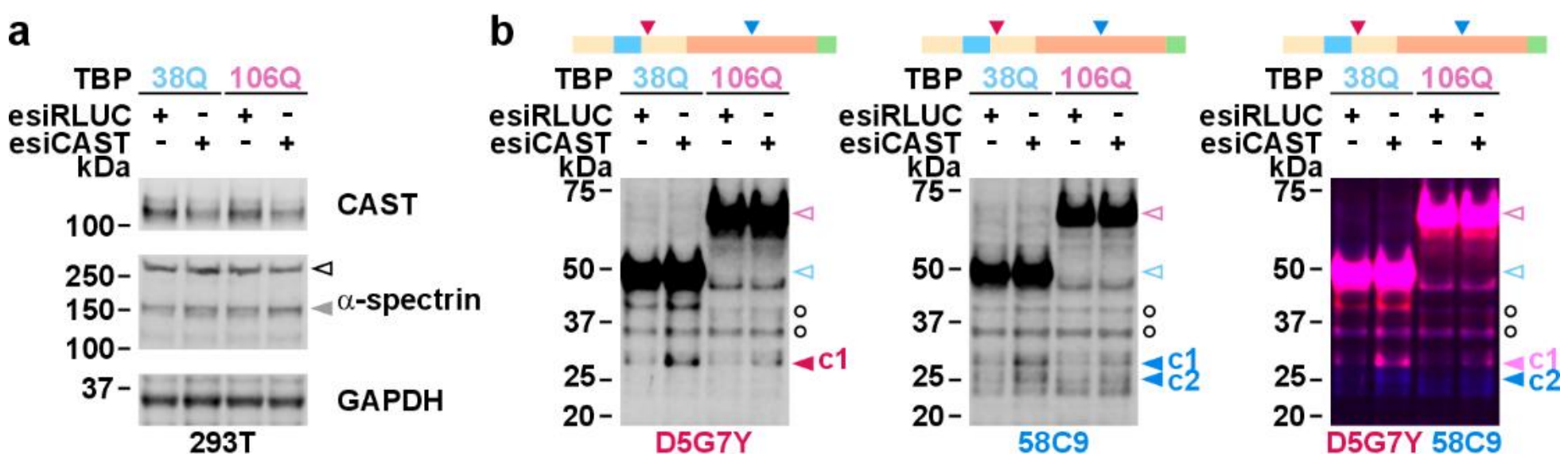

Supplementary Fig. 3: Accumulation of calpain-dependent C-terminal TBP fragments upon *CAST* knockdown. a, 293T cells expressing TBP38Q and 106Q co-transfected with esiRNA directed against the endogenous calpain inhibitor calpastatin (*CAST*). Protein extracts were analysed by western blotting. *Renilla luciferase* targeting esiRNA (esiRLUC) was used as mock control. *CAST* knockdown and its effect on the calpain system were confirmed by antibodies against CAST and the calpain substrate α-spectrin. GAPDH served as loading control. **b,** TBP fragmentation was analysed with TBP-specific antibodies targeting an epitope around amino acid residue A110 (D5G7Y, pink) and the C-terminus (58C9, blue). Light blue- and pink-rimmed arrowheads mark full-length TBP 38Q and 106Q, respectively. Pink and blue arrowheads indicate C-terminal fragments c1 and c2. Black-rimmed circles indicate unspecific bands. Illustration of the approximate binding sites of the antibodies are seen above the corresponding blots. Source data are provided as a Source Data file.

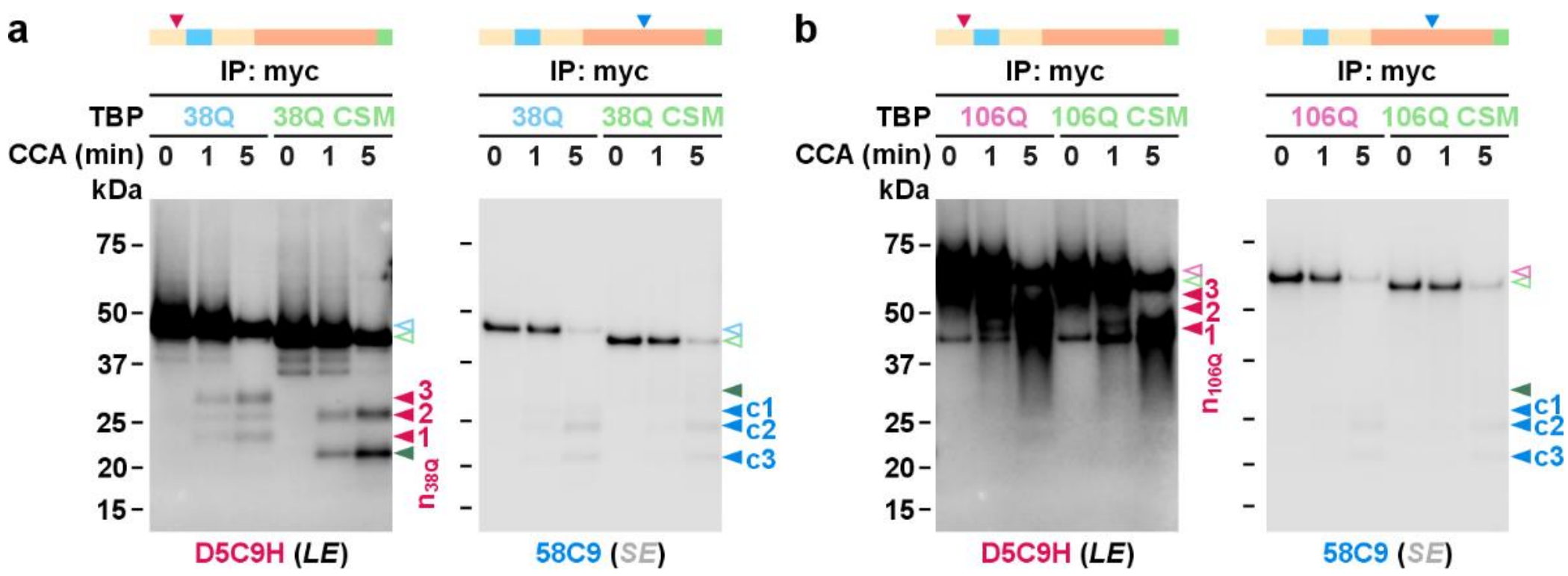

Supplementary Fig. 4: Calpain cleavage site mutation at T106|S107 prevents formation of the TBP c1 fragment. a, b, Western blot-based fragmentation analysis of myc-tagged TBP 38Q (in **a**) and 106Q (in **b**) without and with cleavage site mutation (CSM), after myc tag-specific immunoprecipitation (IP) from 293T cell extracts, followed by a time-dependent CCA with calpain-1. Membranes were detected using N-terminally binding (D5C9H) and C-terminally binding (58C9) TBP antibodies. Light blue- and pink-rimmed arrowheads mark full-length TBP 38Q and 106Q, respectively, and green-rimmed their respective CSM forms. Red and blue arrowheads indicate N-terminal and C-terminal (n1 - 3; c1 - 3) TBP fragments, respectively. Green arrowheads indicate alternative TBP fragments caused by presence of the CSM. The approximate binding sites of the antibodies used are illustrated above the corresponding blots. *SE*, short exposure; *LE*, long exposure. Source data are provided as a Source Data file.

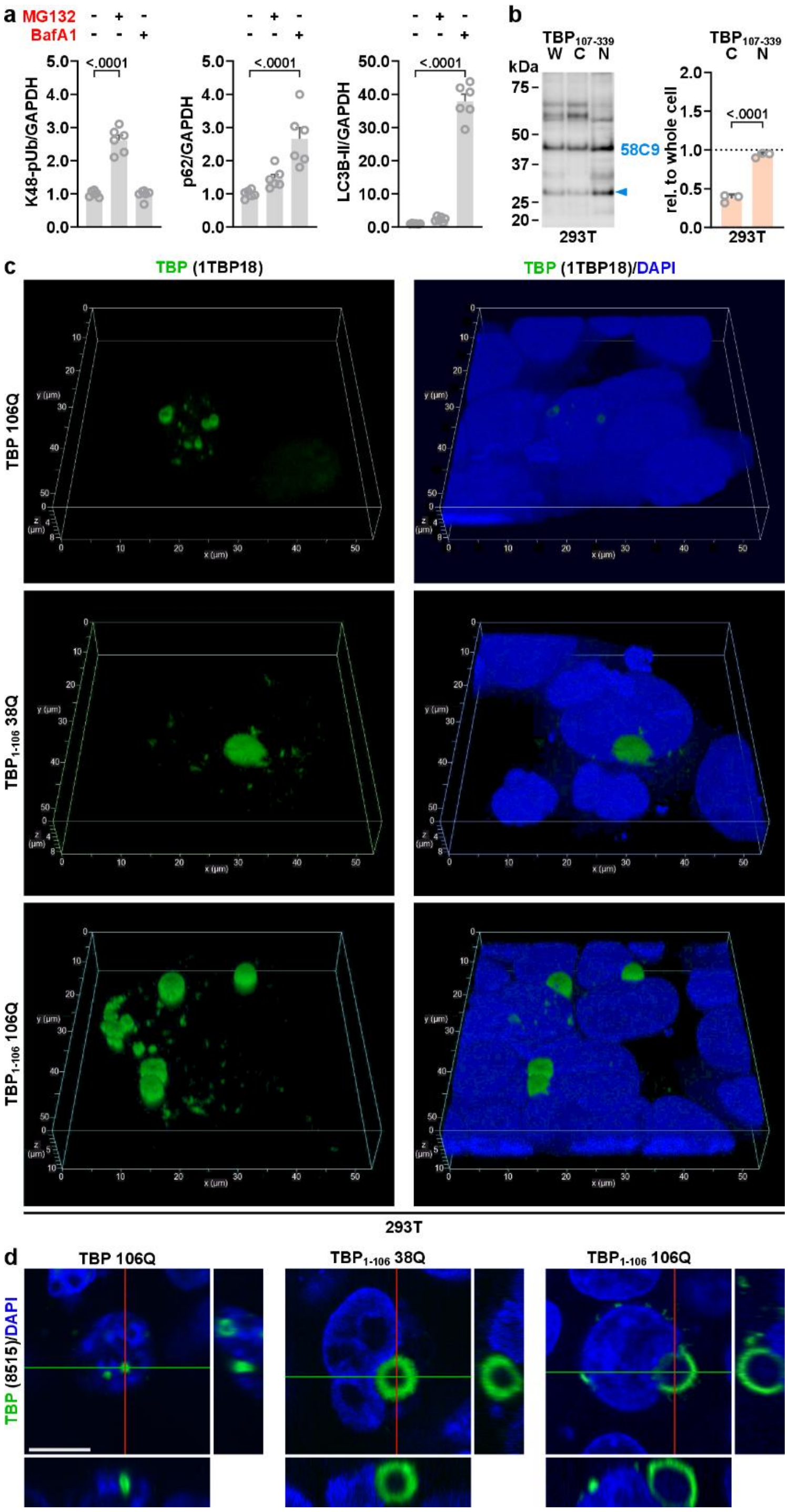

Supplementary Fig. 5: Densitometry of proteasomal and autophagosomal markers, subcellular localisation of $TBP_{107\text{-}339}$, and TBP inclusion body microscopy in 293T cells. **a,** Densitometric analysis of K48-linked polyubiquitin (K48-pUb), SQSTM1/p62 (p62), and MAP1LC3B-II (LC3B-II) (all shown in Fig. 4c) in 293T cells treated with proteasomal inhibitor MG132 or autophagy inhibitor bafilomycin A1 (BafA1). Signals were normalised to GAPDH. *n* = 6 biological replicates. One-way ANOVA with Dunnett's post hoc test. **b,** 293T cells expressing $TBP_{107\text{-}339}$ were subjected to cellular-nuclear fractionation. Obtained fractions (whole cell (W); cytoplasmic fraction (C); nuclear fraction (N)) were analysed by western blot using TBP antibody 58C9. Blue arrowhead marks $TBP_{107\text{-}339}$. Densitometric analysis shows

relative levels of $TBP_{107-339}$ in the cytoplasmic and nuclear fraction. Levels were normalised first to fraction-specific loading controls and then to whole-cell (indicated by a dotted line) within each sample set. *n* = 3 biological replicates. Unpaired *t*-test. **c,** Immunofluorescence staining and confocal microscopy of 293T cells expressing TBP 106Q, TBP_{1-106} 38Q, or TBP_{1-106} 106Q. TBP was detected using the N-terminally binding antibody 1TBP18 (green). DAPI was used as a nuclear counterstain (blue). Three-dimensional reconstructions of confocal z-stack images illustrate the subcellular localisation of TBP inclusion bodies (IBs). Axis scaling (µm) is indicated within the xyz frame. **d,** Immunofluorescence staining and confocal microscopy of 293T cells expressing TBP 106Q, TBP_{1-106} 38Q, or TBP_{1-106} 106Q. The N-terminally binding TBP antibody 8515 was used to analyse subcellular localisation of TBP IBs (green). DAPI was used as a nuclear counterstain (blue). Lines indicate the positions of orthogonal cross sections in y-z (red) and x-z (green) direction, shown in the right and bottom panels, respectively. Scale bar: 10 µm. Bars represent mean ± s.e.m. All tests were two-tailed. *p* values are shown in the graphs. Source data are provided as a Source Data file.

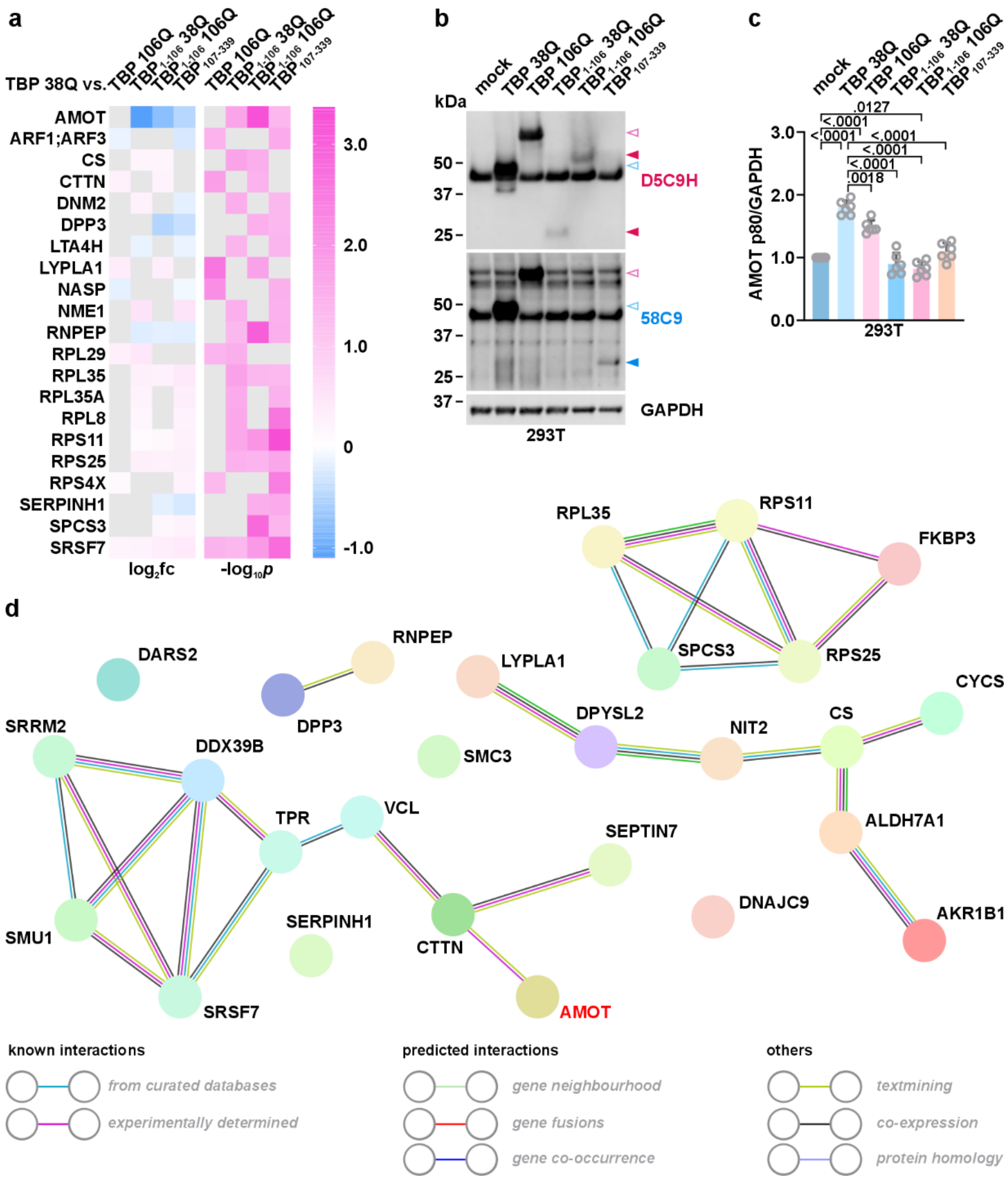

Supplementary Fig. 6: Proteome analysis of 293T cells expressing full-length TBP and TBP fragments. a, Heatmap of proteins identified by quantitative label-free mass spectrometry and significantly dysregulated in at least two conditions (TBP 106Q, $TBP_{1\text{-}106}$ 38Q, $TBP_{1\text{-}106}$ 106Q, or $TBP_{107\text{-}339}$ compared to TBP 38Q. Row-wise calculated log_2fc and $-log_{10}p$ are displayed. n = 3 biological replicates. **b,** Protein extracts of mock-transfected 293T cells or cells expressing full-length TBP and TBP fragments were analysed by western blot. TBP was detected with the N-terminal antibody D5C9H or C-terminal antibody 58C9. GAPDH served as loading control. Light blue- and pink-rimmed arrowheads mark full-length TBP 38Q and 106Q, respectively. Red arrowheads indicate N-terminal fragments ($TBP_{1\text{-}106}$ 38Q or $TBP_{1\text{-}106}$ 106Q), while blue arrowheads indicate C-terminal fragment $TBP_{107\text{-}339}$. **c,** Densitometric analysis of AMOT p80 isoform (shown in Fig. 6g) levels normalised to the loading control. n = 6 biological replicates. One sample t-test and ordinary one-way ANOVA. **d,** STRING network of

differentially regulated proteins detected in 293T cells expressing $TBP_{1\text{-}106}$ 106Q compared to TBP 38Q. Graphical representations of networks were obtained and adapted from STRING (string-db.org). AMOT is highlighted in red. Bars represent mean ± s.e.m. All *t*-tests were two-tailed. *p* values are shown in the graphs. Source data are provided as a Source Data file.

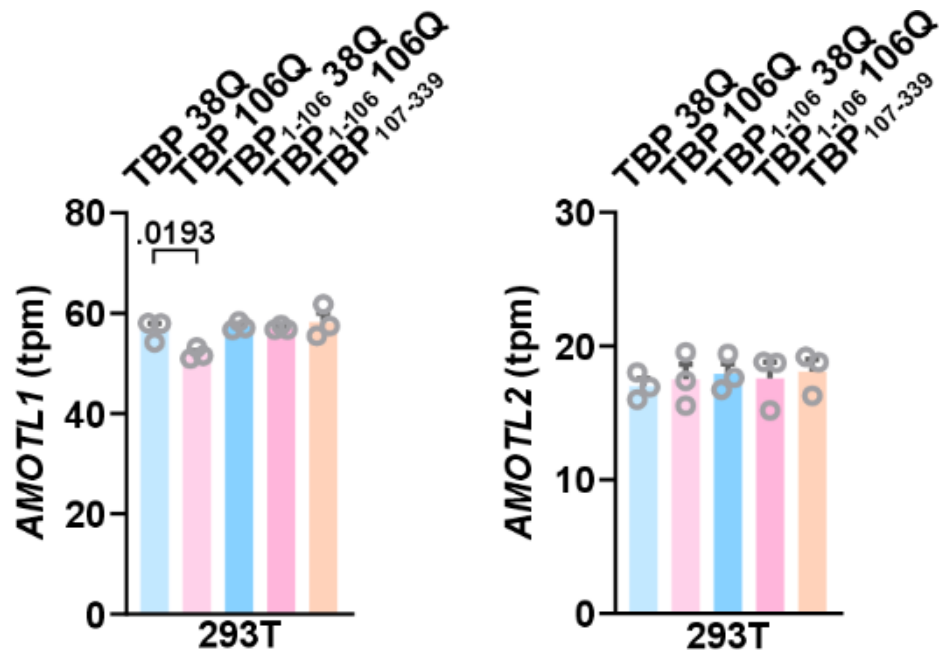

Supplementary Fig. 7: *AMOTL1* and *AMOTL2* transcript levels in 293T cells expressing full-length TBP and TBP fragments. Transcript levels of *AMOTL1* and *AMOTL2* in 293T cells expressing TBP 38Q, TBP 106Q, $TBP_{1\text{-}106}$ 38Q, $TBP_{1\text{-}106}$ 106Q, or $TBP_{107\text{-}339}$ detected by RNA sequencing and shown as transcripts per million (tpm). Bars represent means + s.e.m. *n* = 3 biological replicates. For statistics see Methods: RNA sequencing. Source data are provided as a Source Data file.

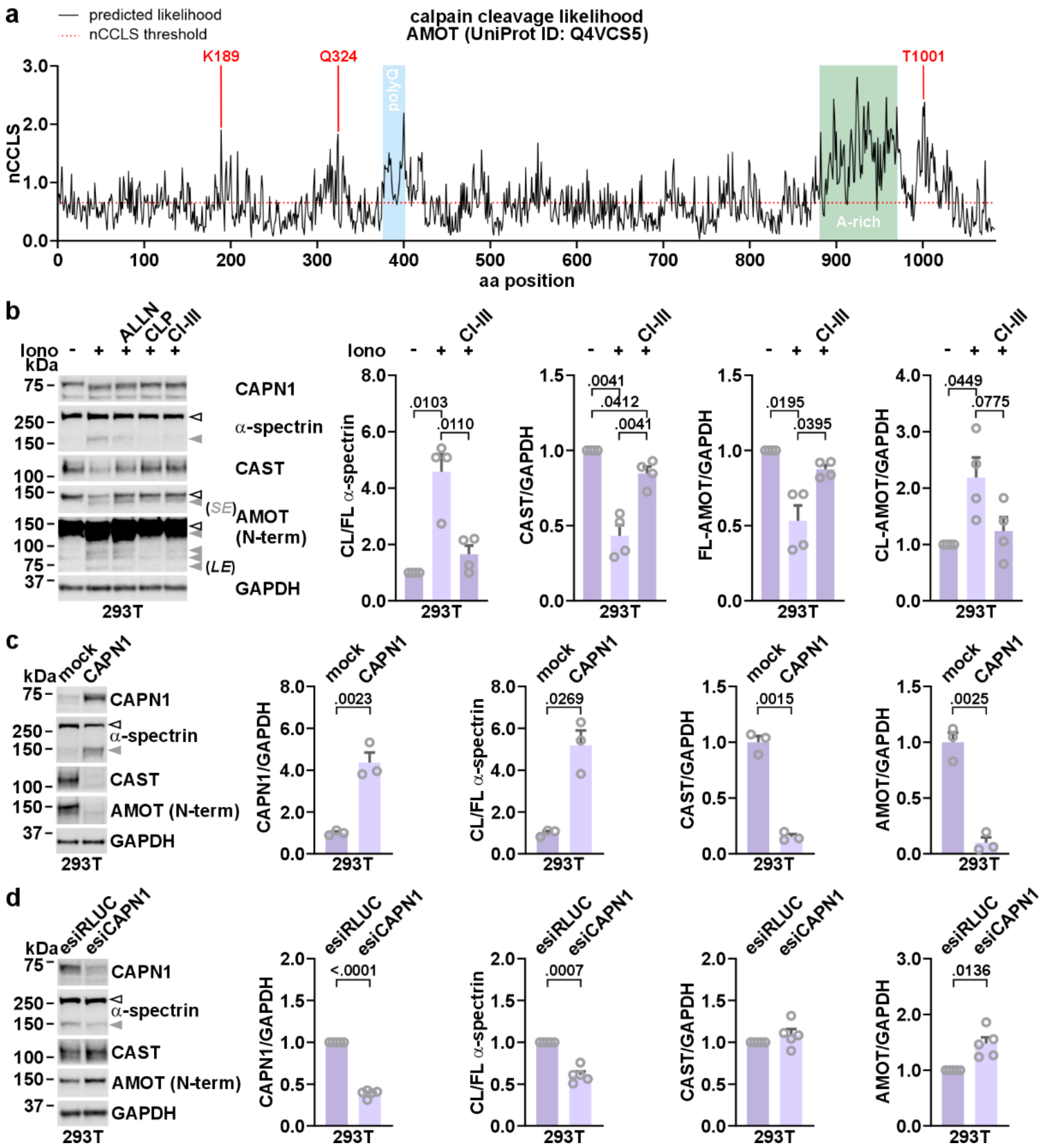

Supplementary Fig. 8: AMOT is a calpain substrate. a, *In silico* prediction of calpain cleavage sites in AMOT (UniProt ID: Q4VCS5) using the GPS-CCD tool. Black lines show normalised calpain cleavage likelihood score (nCCLS) along the protein sequence (left y-axis), with the red-dotted line indicating the likelihood threshold (nCCLS = 1). Prominent sites with high nCCLS values are marked with red vertical lines. The polyQ and alanine (A)-rich regions in AMOT, which may produce prediction artifacts, are highlighted in light blue and green. **b**, Protein extracts of ionomycin (Iono)-treated 293T cells were analysed by western blot to detect calpain-dependent cleavage of AMOT. Calpain activation was confirmed by detecting autoproteolysis of calpain-1 (CAPN1) as well as breakdown of substrates α-spectrin and calpastatin (CAST). Pre-treatments with calpain inhibitor ALLN, calpeptin (CALP), and calpain inhibitor III (CI-III) served as specificity controls. AMOT was detected with an antibody specific for its p130 isoform (N-term). GAPDH was used as the loading control. Black-rimmed arrowhead indicates full-length (FL) proteins, while grey arrowhead shows respective cleavage (CL) products. *SE*, short exposure; *LE*, long exposure. Densitometric analysis shows α-

spectrin cleavage ratio (CL- normalised to FL-α-spectrin), as well as CAST, FL-AMOT, and CL-AMOT levels normalised to GAPDH. Values were normalised to respective mock control of each measurement. *n* = 4 biological replicates. One sample or Student's *t*-test. **c,** Protein extracts of 293T cells transfected with CAPN1 or mock-transfected control cells were analysed by western blot. CAPN1 overexpression and increased calpain activity was confirmed by antibodies against CAPN1 and its substrates α-spectrin and CAST. AMOT was detected with an antibody specific for its p130 isoform (N-term). GAPDH served as the loading control. Black-rimmed arrowhead indicates FL-α-spectrin, while the grey arrowhead shows respective CL product. Densitometric analysis of western blots shows CAPN1 levels normalised to GAPDH, α-spectrin cleavage ratio (CL- normalised to FL-α-spectrin), as well as CAST and AMOT levels, both normalised to GAPDH. Values were normalised to respective mock control of each measurement. *n* = 3 biological replicates. Student's *t*-test. **d,** 293T cells were transfected with esiRNA directed against *CAPN1* and protein extracts were analysed by western blotting. *Renilla luciferase* targeting esiRNA was used as mock control. *CAPN1* knockdown and lowered calpain activity were confirmed by antibodies against CAPN1 and its substrates α-spectrin and CAST. AMOT was detected with an antibody specific for its p130 isoform (N-term). GAPDH served as loading control. Densitometric analysis shows CAPN1 levels normalised to GAPDH, α-spectrin cleavage ratio (CL- normalised to FL-α-spectrin), as well as CAST and AMOT levels, both normalised to GAPDH. Values were normalised to respective mock control of each measurement. *n* = 5 biological replicates. One sample *t*-test. Bars represent mean ± s.e.m. All *t*-tests were two-tailed. *p* values are shown in the graphs. Source data are provided as a Source Data file.

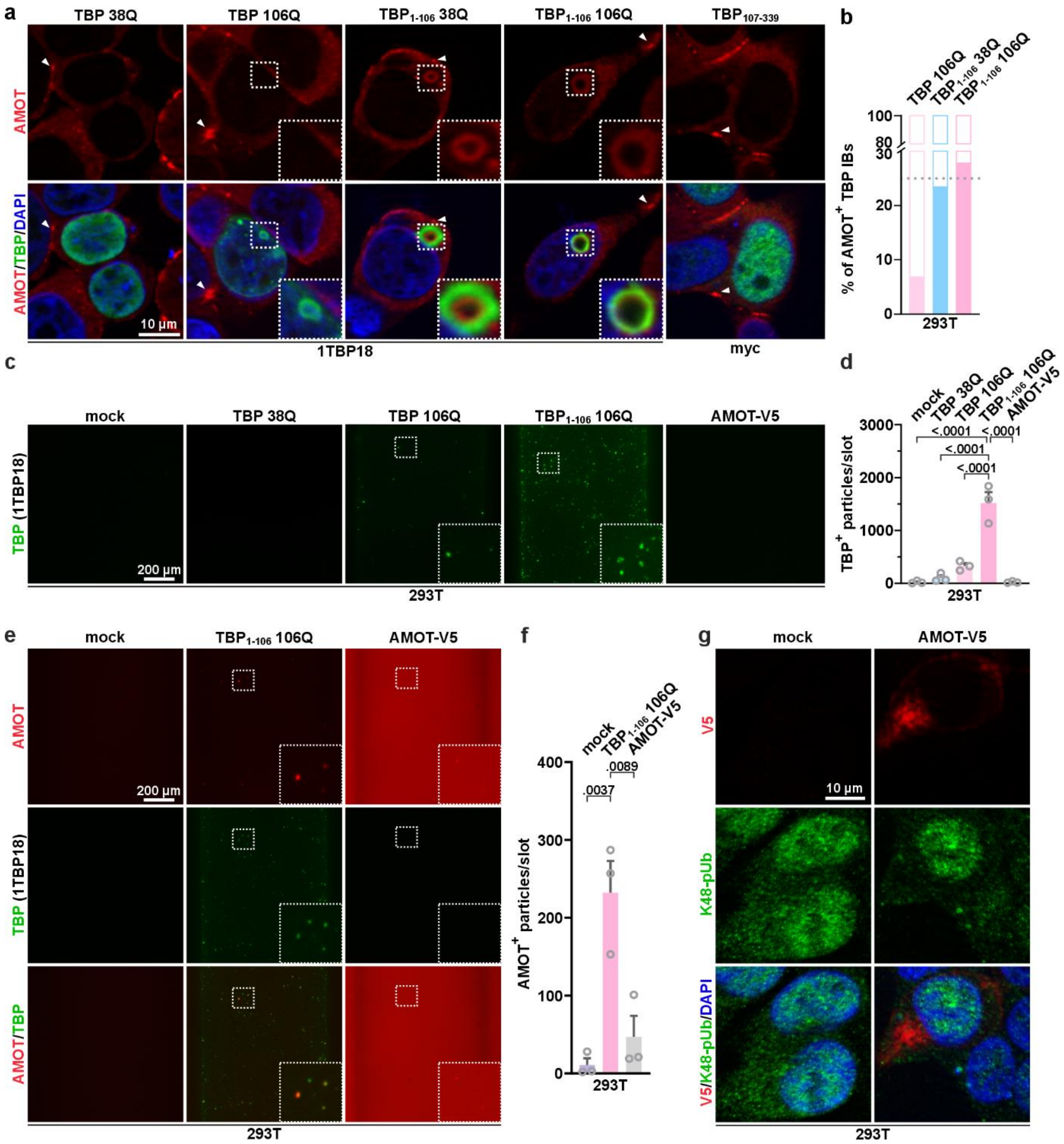

Supplementary Fig. 9: AMOT co-aggregates with TBP. a, Fluorescence microscopy of 293T cells expressing TBP 38Q, TBP 106Q, TBP$_{1\text{-}106}$ 38Q, TBP$_{1\text{-}106}$ 106Q, or TBP$_{107\text{-}339}$. TBP (green) and AMOT (red) were stained with TBP- (1TBP18), myc tag-, and AMOT-specific antibodies to assess co-aggregation. DAPI was used as a nuclear counterstain (blue). Insets show 2.4× magnified views of selected TBP inclusion body (IB)-containing regions (white dashed boxes). Arrowheads indicate local TBP-independent accumulations of AMOT. **b,** Quantification of the proportion of AMOT-positive (AMOT^{+}) TBP IBs in cells expressing TBP 106Q, TBP$_{1\text{-}106}$ 38Q, or TBP$_{1\text{-}106}$ 106Q. Dotted line represents 25% of AMOT^{+} TBP IBs. **c,** Membrane-based inclusion body microscopy of 293T cells expressing TBP 38Q, TBP 106Q, TBP$_{1\text{-}106}$ 106Q, or V5-tagged AMOT, or transfected with an empty vector (mock). Membranes were stained with the TBP-specific antibody 1TBP18 (green) and analysed by fluorescence

microscopy. Insets show 3× magnified views of selected regions (white dashed boxes). **d,** Quantification of TBP-positive (TBP^+) particles per slot. *n* = 3 biological replicates. One-way ANOVA with Tukey's post hoc test. **e,** Membrane-based inclusion body microscopy of 293T cells expressing TBP_{1-106} 106Q or V5-tagged AMOT, or transfected with an empty vector (mock). Membranes were stained with an AMOT-specific antibody and analysed by fluorescence microscopy. Insets show 3× magnified views of selected regions (white dashed boxes). **f,** Quantification of AMOT-positive ($AMOT^+$) particles per slot. *n* = 3 biological replicates. One-way ANOVA with Tukey's post hoc test. **g,** Fluorescence microscopy of 293T cells transfected with empty vector (mock) or V5-tagged AMOT (AMOT-V5). Cells were stained with antibodies against the V5 tag (red) and K48-polyubiquitin (K48-pUb; green). DAPI was used as a nuclear counterstain (blue). Bars represent mean ± s.e.m. All tests were two-tailed. *p* values are shown in the graphs. Source data are provided as a Source Data file.

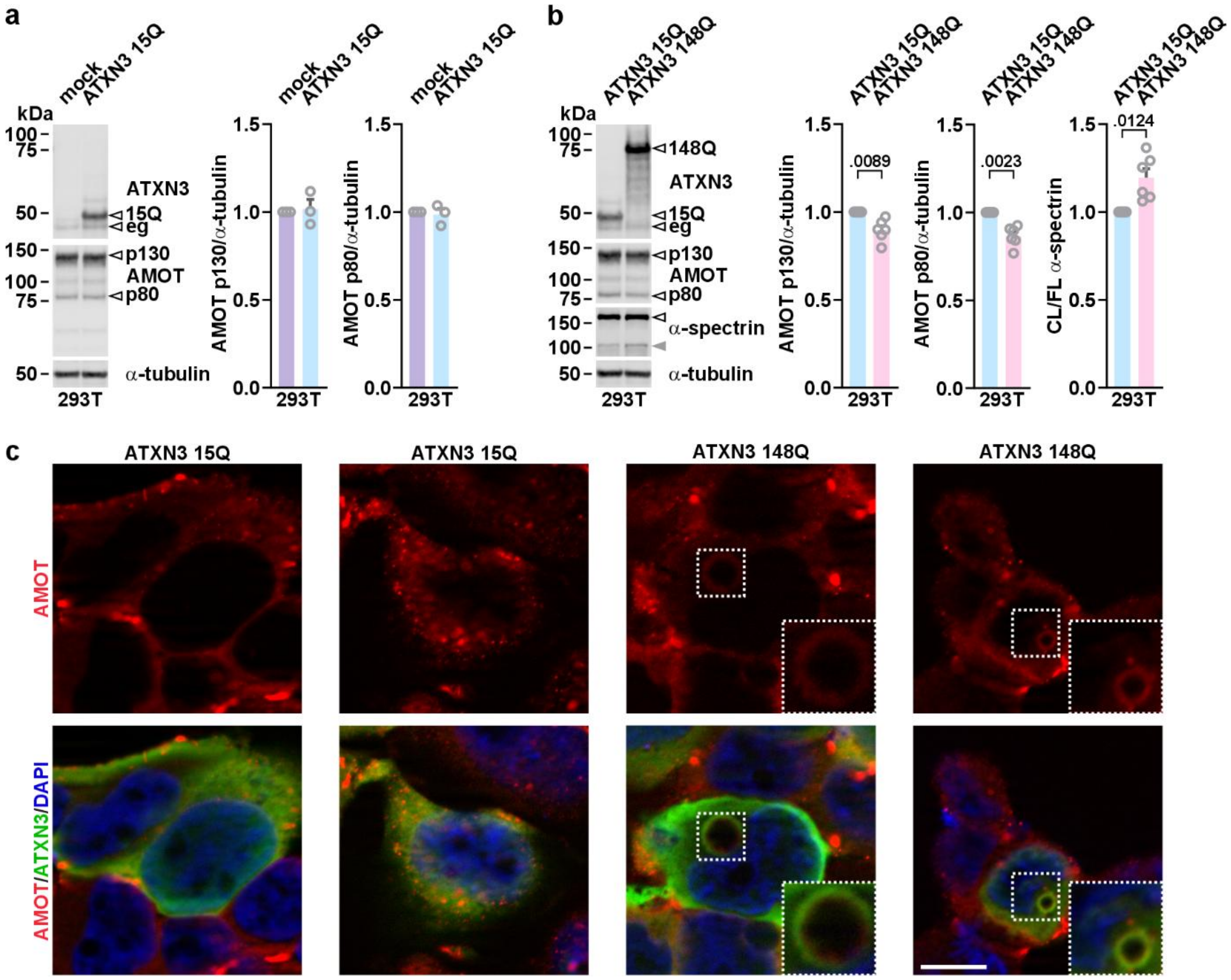

Supplementary Fig. 10: AMOT is reduced and accumulates in aggregates in cells expressing polyQ-expanded ataxin-3. a, Western blot analysis of AMOT levels in 293T cells expressing non-polyQ-expanded ataxin-3 (ATXN3 15Q) and in mock-transfected controls. α-tubulin served as a loading control. Black-rimmed arrowheads indicate endogenous (eg) and overexpressed ATXN3, as well as the p130 and p80 isoforms of AMOT. Densitometric analysis shows AMOT p130 and p80 levels normalised to α-tubulin. *n* = 3 biological replicates. **b,** Western blot analysis of AMOT levels in 293T cells expressing non-polyQ-expanded ATXN3 15Q and polyQ-expanded ATXN3 148Q. Calpain activity was assessed via cleavage of the substrate α-spectrin. α-tubulin served as a loading control. Black-rimmed arrowheads indicate endogenous (eg) and overexpressed ATXN3 (15Q and 148Q), AMOT (p130 and p80), and full-length (FL) α-spectrin. The grey arrowhead marks the calpain-dependent α-spectrin cleavage (CL) product. Densitometric analysis shows AMOT isoform levels normalised to α-tubulin and the α-spectrin cleavage ratio (CL- normalised to FL-α-spectrin). *n* = 6 biological replicates. One sample *t*-test. **c,** Fluorescence microscopy of 293T cells expressing ATXN3 15Q and 148Q. ATXN3 (green) and AMOT (red) were visualised using specific antibodies to assess co-aggregation. Nuclei were counterstained with DAPI (blue). Insets show 2.4× magnified views of selected regions (white dashed boxes). Scale bar: 10 µm. Bars represent

mean ± s.e.m. All *t*-tests were two-tailed. *p* values are shown in the graphs. Source data are provided as a Source Data file.

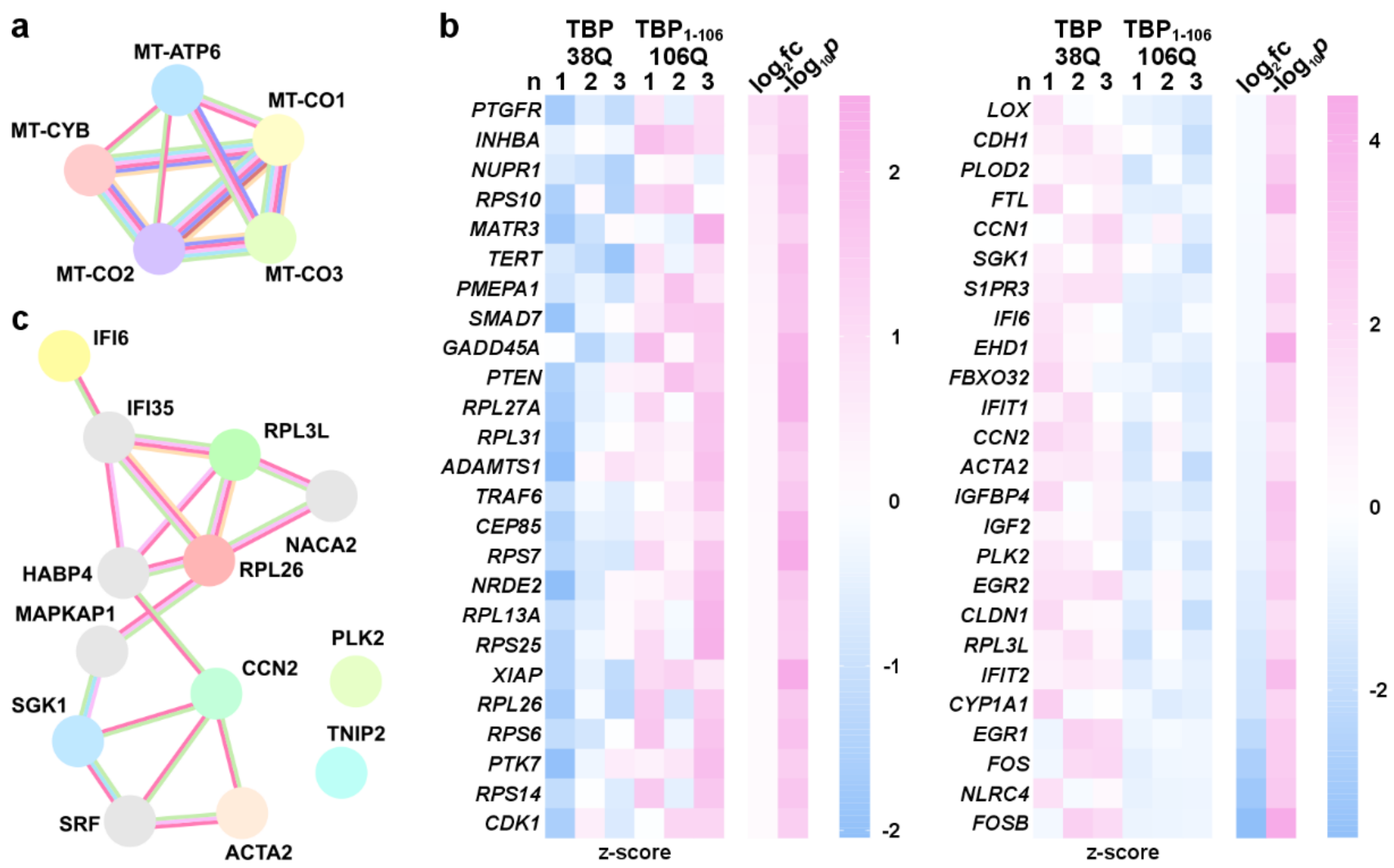

Supplementary Fig. 11: TBP fragments cause transcriptional perturbations in the AMOT-YAP1 signalling. a, STRING network of mitochondrial DEGs shared across TBP 106Q, TBP_{1-106} 106Q, and $TBP_{107-339}$. Blue lines indicate known interactions from curated databases. Lilac lines indicate experimentally determined interactions. Pink lines indicate co-expression. Yellow lines indicate genes that are predicted to be located in neighbourhoods. Purple lines indicate genes that are predicted to co-occur. Green lines indicate text mining. **c,** Heatmap of top 25 up- and downregulated DEGs downstream of the transcription coregulator YAP1 showing row-wise calculated z-scores, log_2fc and $-log_{10}p$ of TBP_{1-106} 106Q compared to TBP 38Q. n = 3 biological replicates. **d,** Extended STRING network of differentially regulated YAP1 downstream genes amongst DEGs shared within comparisons of TBP 106Q and TBP_{1-106} 106Q and $TBP_{107-339}$, TBP 106Q and TBP_{1-106} 106Q, TBP 106Q and $TBP_{107-339}$ as well as TBP_{1-106} 106Q and $TBP_{107-339}$. Predicted network members are marked in grey. Graphical representations of networks were obtained and adapted from STRING (string-db.org). Source data are provided as a Source Data file.

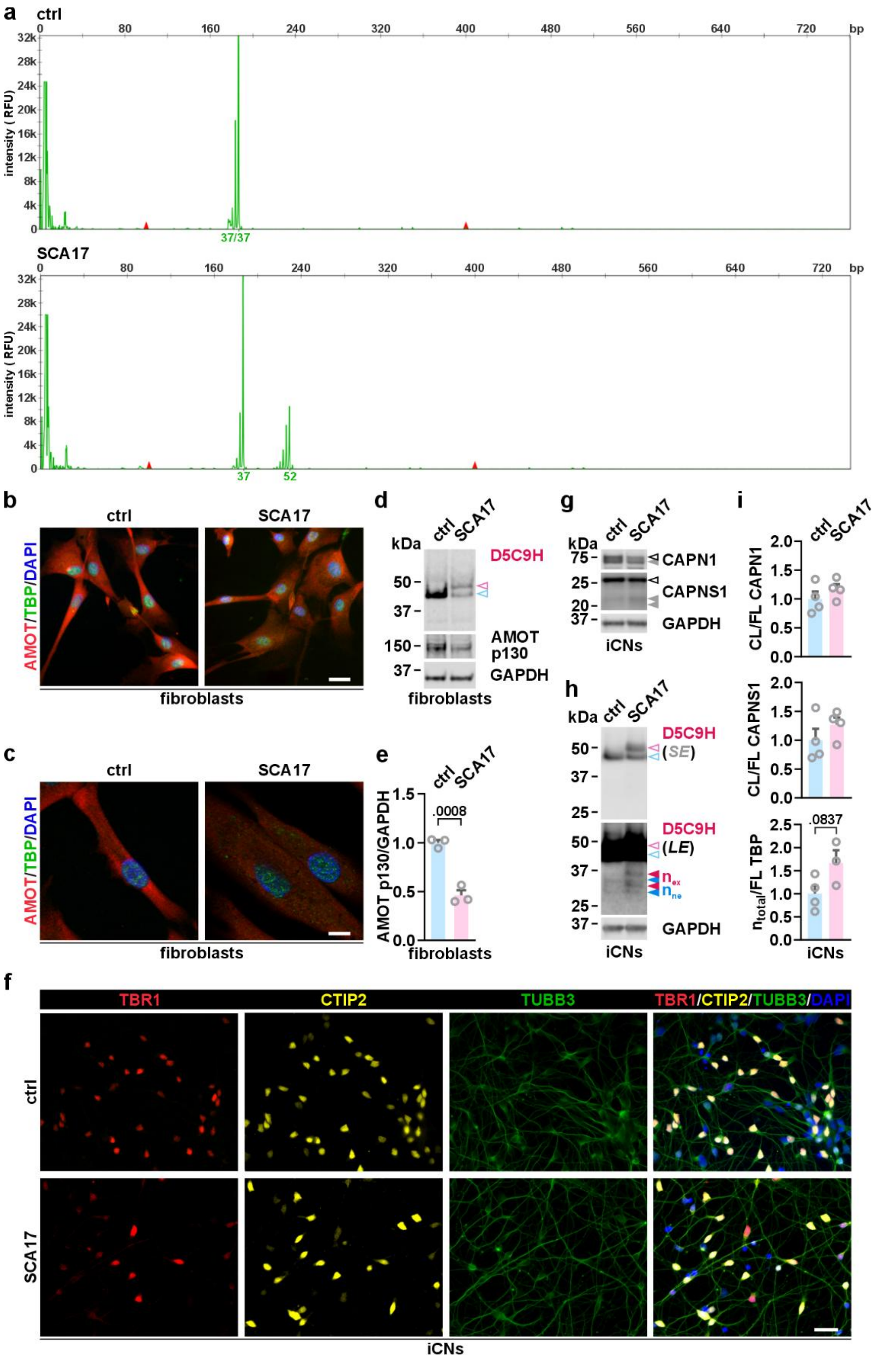

Supplementary Fig. 12: Analysis of SCA17 patient-derived fibroblasts and induced cortical neurons. a, Representative electropherograms of DNA fragment length analysis for *TBP* CAG/CAA repeats in genomic DNA obtained from fibroblasts of an unaffected individual (ctrl) and an SCA17 patient. Calculated repeat lengths (indicated in green) are shown beneath the most intense peaks. RFU, relative fluorescence units. **b, c,** Fluorescence microscopy of fibroblasts from an unaffected individual (ctrl) and an SCA17 patient immunostained for TBP

(green) and AMOT (red). DAPI was used as a nuclear counterstain (blue). Scale bars: 30 µm (**b**) 10 µm (**c**). **d,** Western blot analysis of TBP and AMOT p130 levels in fibroblasts of a ctrl and an SCA17 patient. GAPDH served as the loading control. Light blue-rimmed arrowhead marks full-length non-polyQ-expanded TBP and pink-rimmed arrowhead polyQ-expanded TBP. **e,** Densitometric analysis shows AMOT p130 normalised to the loading control. n = 3 passages. Student's t-test. **f,** Fluorescence microscopy of induced cortical neurons (iCNs) from an unaffected individual (ctrl) and an SCA17 patient immunostained for layer-specific cortical markers TBR1 (for layer 6; red) and CTIP2 (for layer 5; yellow), and for the pan-neuronal marker βIII-tubulin (TUBB3; green). DAPI was used as a nuclear counterstain (blue). Scale bar: 30 µm. **g,** Western blot analysis of calpain-1 (CAPN1) and the small calpain subunit 1 (CAPNS1) in iCNs of a ctrl and an SCA17 patient. GAPDH served as the loading control. Black-rimmed arrowheads indicate full-length (FL) proteins, while grey arrowhead shows respective cleavage (CL) products. **h,** Western blot analysis of TBP fragmentation in ctrl and SCA17 iCNs using the N-terminally binding TBP antibody D5C9H. GAPDH served as the loading control. Light blue-rimmed arrowheads mark non-polyQ-expanded FL-TBP and pink-rimmed arrowheads polyQ-expanded FL-TBP. Light blue arrowheads indicate N-terminal fragments of non-expanded TBP (n_{ne}) and pink arrowheads of polyQ-expanded TBP (n_{ex}). *SE*, short exposure. *LE*, long exposure. **i,** Densitometric analysis of cleaved CAPN1, cleaved CAPNS1, and total N-terminal (n_{total}) TBP fragments, normalised to the respective full-length protein. n = 3 (SCA17 in n_{total}/FL TBP) or 4 (ctrl and SCA17 in CL/FL CAPN1, and CL/FL CAPNS1; ctrl in n_{total}/FL TBP) biological replicates. Student's t-test. Bars represent means + s.e.m. All t-tests were two-tailed. p values are shown in the graphs. Source data are provided as a Source Data file.

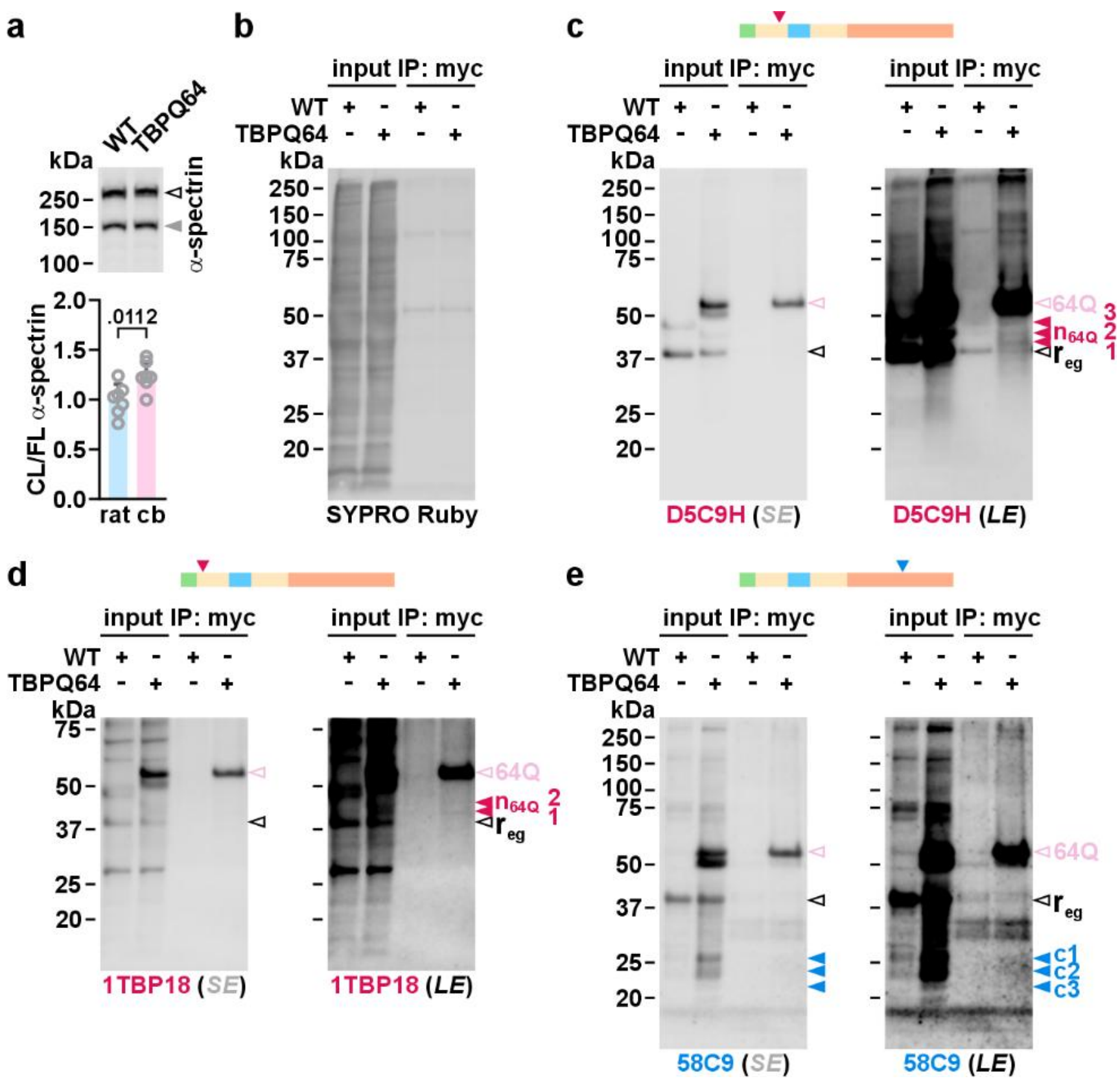

Supplementary Fig. 13: Calpain activation of occurrence of N-terminal TBP fragments in SCA17 rat cerebellum. a, Western blot analysis of cerebellar (cb) protein extracts from wild-type (WT) and transgenic SCA17 (TBPQ64) rats expressing myc-tagged polyQ-expanded human TBP with 64 glutamines. Calpain activation was analysed by detecting cleavage of the substrate α-spectrin. Black-rimmed arrowhead marks full-length α-spectrin, grey arrowhead marks calpain-dependent α-spectrin breakdown product. Densitometric analysis shows α-spectrin cleavage ratio (breakdown product (CL) normalized to full-length (FL) α-spectrin) in TBPQ64 rats relative to WT rats. *n* = 7 animals. Bars represent means + s.e.m. Two-tailed Student's *t*-test. *p* values are shown in the graphs. **b-e,** Western blot analysis of N-terminal TBP 64Q fragments after enrichment using myc tag-specific immunoprecipitation (IP). Full protein extracts from WT and TBPQ64 rat cerebellum (input) were loaded along the IP samples. SYPRO Ruby staining served as loading control and for validation of IP purity (in **b**). Membranes were probed with N-terminally binding TBP-specific antibodies (D5C9H in **c** and 1TBP18 in **d**) or the C-terminally binding TBP antibody 58C9 (in **e**). Light pink-rimmed arrowheads mark transgenic full-length myc-TBP 64Q and black-rimmed arrowheads endogenous rat TBP (r_{eg}). Red and blue arrowheads indicate N- and C-terminal (n1 - 3; c1 - 3) TBP fragments, respectively. The approximate binding sites of the antibodies used are illustrated above the corresponding blots. *SE*, short exposure; *LE*, long exposure. Source data are provided as a Source Data file.

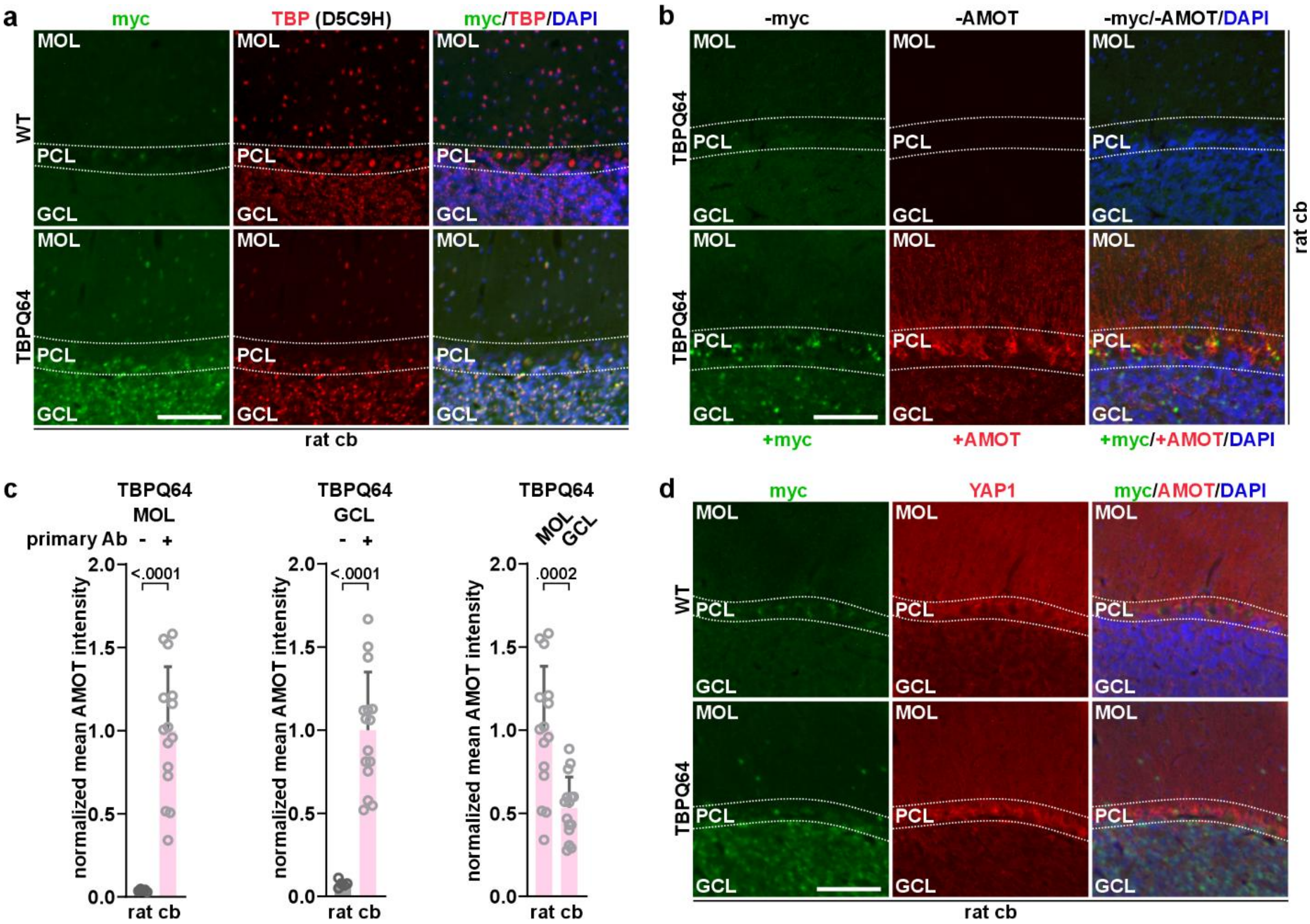

Supplementary Fig. 14: Fluorescence immunohistochemistry-based analysis of SCA17 rat cerebellum. a, Immunofluorescent staining and microscopy of cerebellum (cb) sections from 10-month-old wild-type (WT) and transgenic SCA17 (TBPQ64) rats expressing N-terminally myc-tagged, polyQ-expanded human TBP with 64 glutamines. Transgene expression was validated using a myc-tag specific antibody (in green). Total TBP was detected using the D5C9H antibody (in red). DAPI was used as a nuclear counterstain (blue). MOL, molecular layer; PCL, Purkinje cell layer; GCL, granular cell layer. Scale bar: 100 µm. **b,** Specificity of immunostaining for myc-tagged transgenic TBP (in green) and endogenous AMOT (antibody ABS1024; in red) in WT and TBPQ64 rat brain sections was tested in absence (-) or presence (+) of the primary antibody. DAPI was used as a nuclear counterstain (blue). Scale bar: 100 µm. **c,** Quantitative analysis of AMOT staining in the MOL and GCL of TBPQ64 rat cerebellum relative to primary antibody-negative control staining. Signals were normalized to section stained with the primary antibody. Additional normalization of GCL to MOL signals show a twofold higher AMOT intensity in the MOL. *n* = 1 (antibody-negative control) and 3 (with primary antibody) animals, 5 images per animal. Bars represent means + s.e.m. Two-tailed Unpaired *t*-test. *p* values are shown in the graphs. **d,** Immunofluorescent staining and microscopy of myc-TBP 64Q (in green) and YAP1 (in red) in WT and TBPQ64 rat cerebellum, shows reduced YAP1 signals in the MOL. Myc-TBP signals are shown in green, and AMOT signals in red. DAPI was used as a nuclear counterstain (blue). Scale bar: 100 µm. Source data are provided as a Source Data file.

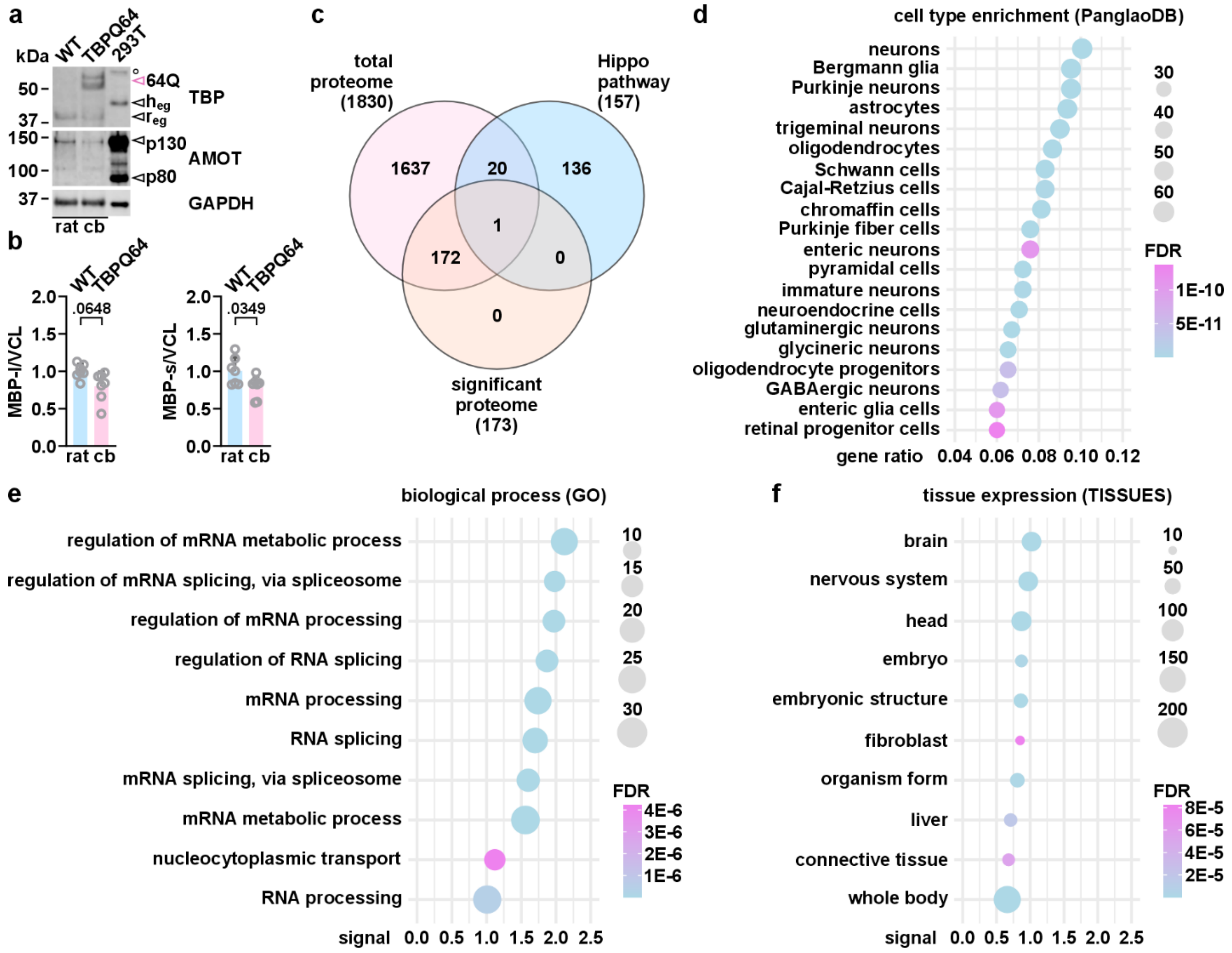

Supplementary Fig. 15: Proteomic analysis of SCA17 rat cerebellum. a, Western blot analysis of cerebellar (cb) protein extracts from wild-type (WT) and SCA17 TBPQ64 rats, loaded along a protein extract from 293T cells. PolyQ-expanded TBP (64Q) in the TBPQ64 rat, endogenous rat TBP (r_{eg}), and endogenous human TBP (h_{eg}), as well as AMOT isoforms p130 (in rat and 293T cells) and p80 (only in 293T cells) were detected. GAPDH served as the loading control. Circle (°) marks an unspecific protein band. **b,** Densitometric analysis of long (l) and short (s) isoforms of myelin basic protein (MBP) in cerebellar protein extracts from WT and TBPQ64 rats (for western blot detection see Fig. 9d), normalised to loading control vinculin (VCL). $n = 7$ animals. Student's *t*-test. **c,** Venn diagram illustrates overlap of 1830 detected and 173 significantly dysregulated proteins in rat cerebellum, of which 21 are members of the YAP1-regulated Hippo pathway (157 molecules; KEGG database, entry: hsa04390) and only one protein (RPL12) being significantly altered. **d,** PanglaoDB-based cell type enrichment analysis for entire detected LFQ proteome in TBPQ64 rat cerebellum. Bubble plots show ranking by gene ratio, with bubble colour indicating FDR and bubble size gene count. **e, f,** Bubble plot of top ten enrichments in the TBPQ64 rat cerebellum proteome for Biological Process (Gene Ontology; in **e**) and Tissue Expression (TISSUES; in **f**) obtained from STRING (string-db.org), ranked by signal. Bubble colour indicates FDR and bubble size protein counts.

Bars represent means + s.e.m. All *t*-tests were two-tailed. *p* values are shown in the graphs. Source data are provided as a Source Data file.

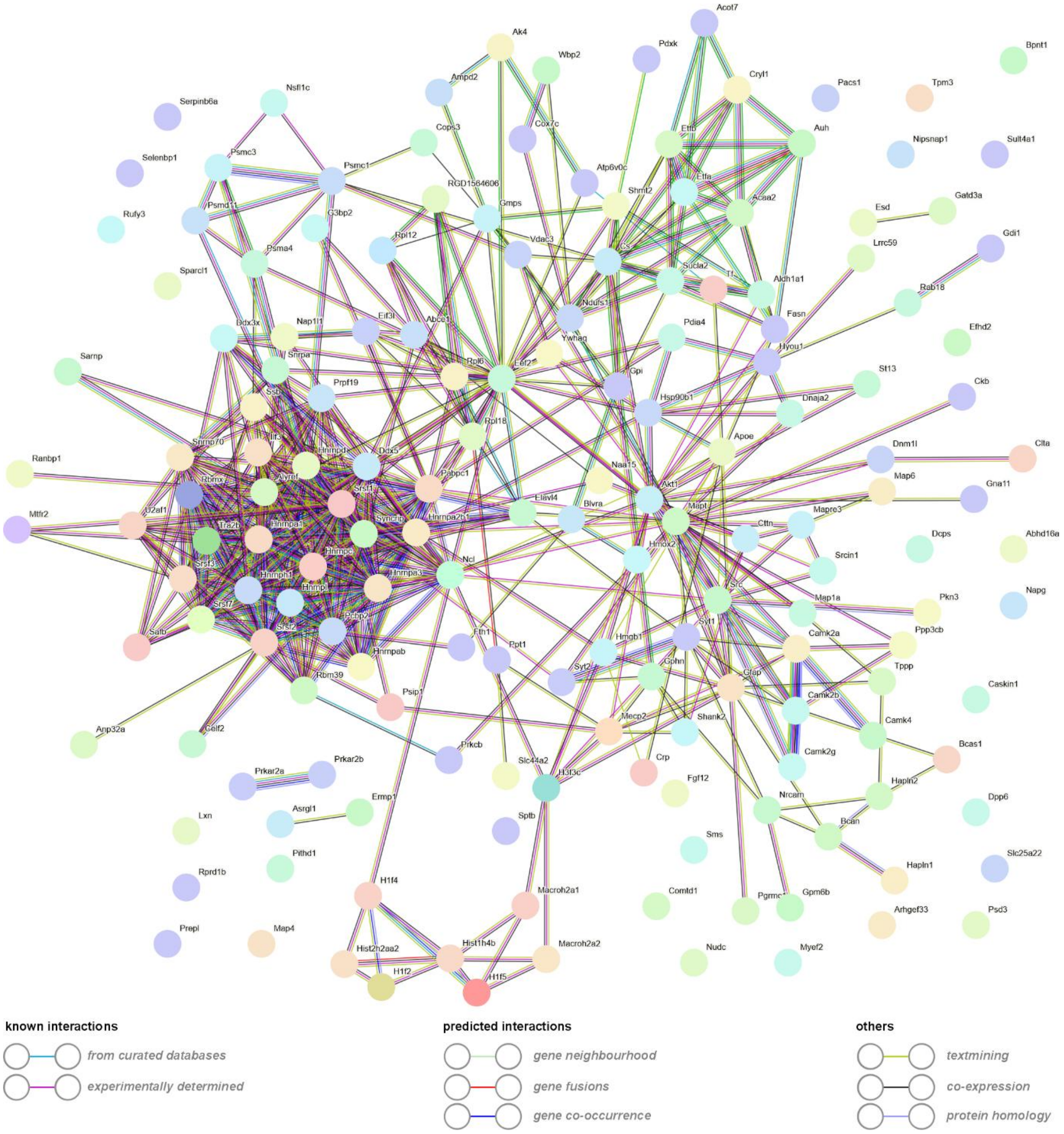

Supplementary Fig. 16: STRING network of the dysregulated cerebellar proteome in SCA17 rats. STRING network of differentially regulated proteins detected in SCA17 rat cerebellum compared to wild-type tissue (167/173 found in database). Graphical representations of networks were obtained and adapted from STRING (string-db.org). Source data are provided as a Source Data file.

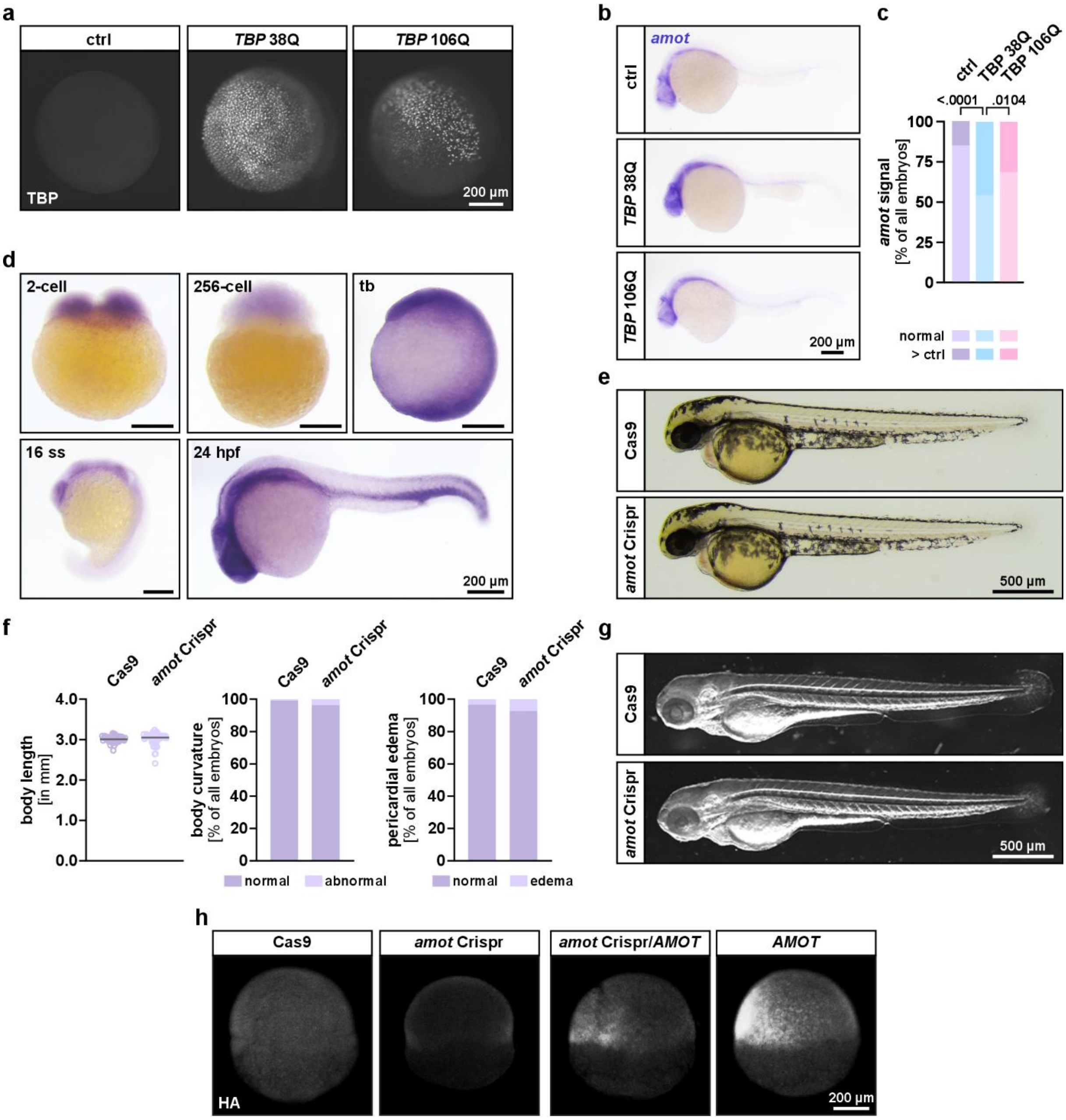

Supplementary Fig. 17: TBP overexpression, *amot* expression during embryonal development, as well as effects of *amot*-specific Crispr/Cas9 strategy and *AMOT* rescue in zebrafish. a, Antibody staining against human TBP upon injection of control RNA (ctrl) or capped RNA encoding TBP 38Q or TBP 106Q. 50-60% epiboly. **b, c,** *In situ* hybridization for *amot* (purple signals) in 27 hpf zebrafish embryos, injected with a control RNA (ctrl) or RNA encoding human TBP 38Q or TBP 106Q. *n* = 7 experiments with 180 (ctrl), 120 (TBP 38Q), and 185 (TBP 106Q) embryos in total. Fisher's exact test. **d,** Zebrafish *amot* (purple signals) is already provided maternally as shown by expression at the 2-cell and 256-cell stage. After maternal-to-zygotic transition, *amot* remains highly and ubiquitously expressed as shown at tailbud (tb) stage. During organogenesis (16 somite stage, ss), *amot* becomes restricted to neuronal and mesodermal structures. When the body plan has been established (24 hours post fertilization, hpf), transcripts can be detected in the developing vasculature and in

neuronal structures such as the neural tube. **e, f,** Morphology and phenotypical analysis, including body length, body curvature and occurrence of pericardial edema, in 48 hpf zebrafish embryos injected with Cas9 alone as control (Cas9) or a ribonucleoprotein consisting of both *amot*-specific guideRNAs and Cas9 (*amot* Crispr). **g,** Morphology of 72 hpf Cas9-injected control or *amot* crispant embryos. **h,** Antibody staining against HA-tagged human AMOT in zebrafish embryos (50-60% epiboly) upon injection of Cas9 alone, of guideRNAs and Cas9 (*amot* Crispr), of a combination of guideRNAs and Cas9 with capped RNA encoding HA-tagged human AMOT (*amot* Crispr/*AMOT*), or capped RNA for HA-AMOT alone (*AMOT*). All tests were two-tailed. Grey horizontal lines in graphs indicate the median. *p* values are shown in the graphs. Source data are provided as a Source Data file.

Supplementary Tables

Supplementary Table 1: TBP peptides derived from immunocapture-calpain-cleavage assay and identified by mass spectrometry.

Peptide	Start position	End position	Length	Total count	Count per exp.	# of exp. with hit
EQKLISEEDLN	346	356	11	1	0.33	1
EQKLISEEDLNMH	346	358	13	5	1.67	3
EQKLISEEDLNMHTGH	346	361	16	4	1.33	3
TGAKVRAEIY	313	322	10	2	0.67	2
SSGKMVCTGAKSEEQSRL	215	232	18	5	1.67	2
EAFENIYPILK	323	333	11	1	0.33	1
RARNAEYNPKRF	186	197	12	1	0.33	1
NAEYNPKRF	189	197	9	3	1.00	2
QATQGTSGQAPQLF	109	122	14	1	0.33	1
QSTSQQATQGTSGQAPQLF	104	122	19	1	0.33	1
SQQATQGTSGQAPQLF	107	122	16	3	1.00	3
STSQQATQGTSGQAPQLF	105	122	18	1	0.33	1
TSQQATQGTSGQAPQLF	106	122	17	1	0.33	1

Calpain cleavage-derived peptide termini are indicated in green. Chymotryptic termini are indicated in red. Peptides derived from the C-terminal myc tag of TBP are marked grey. Source data are provided as a Source Data file.

Supplementary Table 2. Primary antibodies for immunocytochemistry and immunohistochemistry staining.

Antibody/target	Host	Dilution	Clone/catalogue no.	Vendor
AMOT	rabbit	1:400	24550-1-AP	Proteintech
AMOT	rabbit	1:100*	ABS1024	Merck
ataxin-3 (ATXN3)	mouse	1:500	1H9-2/650401	BioLegend
calpain-1 (CAPN1)	rabbit	1:100	10538-1-AP	Proteintech
calpain-2 (CAPN2)	mouse	1:500	1E1F10/66977-1-Ig	Proteintech
calpain-5 (CAPN5)	rabbit	1:250	ab38943	Abcam
calpain-7 (CAPN7)	rabbit	1:250	ab28211	Abcam
calpain-10 (CAPN10)	rabbit	1:250	ab28220	Abcam
calpain-15 (CAPN15)	rabbit	1:100	33924-1-AP	Proteintech
calpain small subunit 1 (CAPNS1)	mouse	1:100	P1/MAB3083	Merck
calpastatin (CAST)	rabbit	1:50	sc-20779	Santa Cruz Biotechnology
CTIP2	rat	1:200	25B6/ab18465	Abcam
MAP2	chicken	1:2,500	Poly28225/822501	BioLegend
myc tag	mouse	1:500/1:50*	9B11/#2276	Cell Signaling Technology
myc tag	rabbit	1:500*	71D10/#2278	Cell Signaling Technology
TBP	mouse	1:500/1:50*	1TBP18/sc-56794	Santa Cruz Biotechnology
TBP	rabbit	1:250	#8515	Cell Signaling Technology
TBP	rabbit	1:100*	D5C9H/#44059	Cell Signaling Technology
TBR1	rabbit	1:100	EPR8138(2)/ab183032	Abcam
βIII-tubulin	mouse	1,1,000	SDL.3D10/T8660	Merck
ubiquitin (K48-linkage specific)	rabbit	1:100	APU2/ZRB2150	Sigma Aldrich/Merck
V5 tag	mouse	1:500	SV5-Pk1	Invitrogen
YAP1	rabbit	1:100*	13584-1-AP	Proteintech

Antibodies were diluted in ICC antibody diluent (1% (w/v) BSA, 0.5% (v/v) Triton X-100, and 0.02% (w/v) NaN_3 in 1× DPBS). *Antibodies used for immunohistochemistry were diluted accordingly in IHC antibody diluent (2% (v/v) normal goat serum, 3% (w/v) BSA, 0.1% (v/v) Triton X-100, and 0.02% (w/v) NaN_3 in 1× DPBS). **Antibody dilution of immunocytochemistry staining of induced cortical neurons.

Supplementary Table 3. Primary antibodies for membrane-based immunodetection.

Antibody/target	Host	Dilution	Clone/catalogue no.	Vendor
β-actin	mouse	1:5,000	AC-15/A5441	Sigma-Aldrich
AMOT	mouse	1:500	sc-166924	Santa Cruz Biotechnology
AMOT	mouse	1:500	sc-517353	Santa Cruz Biotechnology
AMOT	rabbit	1:500	ABS1024	Merck
AMOT	rabbit	1:4,000	24550-1-AP	Proteintech
AMOT	rabbit	1:1,000	16870-1-AP	Proteintech
ataxin-3 (ATXN3)	mouse	1:2,000	1H9/MAB5360	Merck
calpain-1 (CAPN1)	rabbit	1:1,000	ab39170	Abcam
calpain-2 (CAPN2)	mouse	1:1,000	1E1F10/66977-1-Ig	Proteintech
calpain small subunit 1 (CAPNS1)	mouse	1:500	P-1//MAB3083	Merck
calpastatin (CAST)	rabbit	1:1,000*	#4146	Cell Signaling Technology
CYR61/CCN1	rabbit	1:500	26689-1-AP	Proteintech
CTGF/CCN2	rabbit	1:500	25474-1-AP	Proteintech
GAPDH	mouse	1:2,000	0411/sc-47724	Santa Cruz Biotechnology
GAPDH	rabbit	1:5,000	10494-1-AP	Proteintech
Lamin A/C	mouse	1:1,000	E-1/sc-376248	Santa Cruz Biotechnology
Lamin A/C	rabbit	1:2,500	H-110/sc-20681	Santa Cruz Biotechnology
MAP1LC3B (LC3B)	rabbit	1:500*	#2775	Cell Signaling Technology
Myelin basic protein (MBP)	rabbit	1:1000	ab40390	Abcam
myc tag	mouse	1:500*	9B11/#2276	Cell Signaling Technology
myc tag	rabbit	1:500*	71D10/#2278	Cell Signaling Technology
polyglutamine (polyQ)	mouse	1:1,000	5TF1-1C2/MAB1574	Merck
SQSTM1/p62 (p62)	rabbit	1:1,000*	#5114	Cell Signaling Technology
αII-spectrin	mouse	1:1,000	AA6/MAB1622	Merck
TBP	mouse	1:500	1TBP18/sc-56794	Santa Cruz Biotechnology
TBP	mouse	1:1,000	58C9/T1827	Sigma-Aldrich
TBP	mouse	1:200	58C9/sc-421	Santa Cruz Biotechnology
TBP	rabbit	1:500*	#8515	Cell Signaling Technology
TBP	rabbit	1:500*	D5C9H/#44059	Cell Signaling Technology

TBP	rabbit	1:500*	D5G7Y/#12578	Cell Signaling Technology
α-tubulin	mouse	1:5,000	DM1A/CP06	Merck
α-tubulin	rabbit	1:1,000	11224-1-AP	Proteintech
ubiquitin (K48-linkage specific)	rabbit	1:1,000*	D9D5/#8081	Cell Signaling Technology
Vinculin (VCL)	rabbit	1:1,000*	E1E9V/#13901	Cell Signaling Technology
YAP1	rabbit	1:1,000	13584-1-AP	Proteintech

All antibodies were diluted in 1× TBS with 0.1% (v/v) Tween-20 and 0.02% NaN_3. *Antibody dilutions were supplemented with 5% (w/v) bovine serum albumin.
