# Supplementary material for: Calpain-mediated TBP cleavage disturbs neuronal AMOT-YAP1 signalling
Source: Nat Commun. 2026 Nov 9;17:11531. doi: 10.1038/s41467-026-79055-x (PMC13748621; doi:10.1038/s41467-026-79055-x)
Supplement: Supplementary file 2 [file 41467_2026_79055_MOESM2_ESM.pdf]

Title: Supplementary Data 1
Description: Proteins identified by mass spectrometry as dysregulated upon expression of polyQ-expanded TBP or TBP fragments in 293T cells.

Title: Supplementary Data 2
Description: Neuronal associations of proteins dysregulated in 293T cells expressing TBP1-106 106Q and grouped in an interaction network with AMOT.

Title: Supplementary Data 3
Description: YAP1 downstream genes dysregulated in 293T cells expressing TBP1-106 106Q compared TBP 38Q.

Title: Supplementary Data 4
Description: Diseases and functions identified by Ingenuity Pathway Analysis, based on differentially expressed genes in 293T cells expressing polyQ-expanded TBP or TBP fragments.

Title: Supplementary Software 1
Description: ImageJ/Fiji macro for semi-automated quantification of TBP inclusion bodies. The macro (Code_FIJImacro_aggregate_quantification.fiji.ijm) processes multichannel fluorescence microscopy images of immunostained cells. Users define the input and output folders, image file suffix, channel of interest and magnification, can preselect regions of interest, and adjust intensity thresholds before particle analysis. Measurements of individual particles are exported as an Excel file, and an image of the detected particles is saved for quality control. This macro was used to generate the data shown in Fig. 5 c) and Supplementary Fig. 9 d) and e). The code is available on Zenodo (https://doi.org/10.5281/zenodo.22231266).

Title: Supplementary Software 2.
Description: ImageJ/Fiji macro for semi-automated quantification of fluorescence signal intensity in tissue sections. The macro (Code_FIJImacro_signal_intensity_quantification_tissue.fiji.ijm) processes multichannel fluorescence microscopy images of immunostained rodent tissue. For each image, users select a region of interest and an adjacent background region. Within the region of interest, nuclei are counted automatically and signal intensities are measured and background-corrected. Results are exported as an Excel file, and images of the selected areas are saved for quality control. This macro was used to generate the data shown in Fig. 9 b) and Supplementary Fig. 14 c). The code is also available on Zenodo (https://doi.org/10.5281/zenodo.22231266).
