# Supplementary material for: Calpain-mediated TBP cleavage disturbs neuronal AMOT-YAP1 signalling
Source: Nat Commun. 2026 Nov 9;17:11531. doi: 10.1038/s41467-026-79055-x (PMC13748621; doi:10.1038/s41467-026-79055-x)
Supplement: Supplementary file 3 [file 41467_2026_79055_MOESM8_ESM.pdf]

nature portfolio

Corresponding author(s): Jonasz Jeremiasz Weber

Last updated by author(s): Sep 15, 2026

Reporting Summary

Nature Portfolio wishes to improve the reproducibility of the work that we publish. This form provides structure for consistency and transparency in reporting. For further information on Nature Portfolio policies, see our Editorial Policies and the Editorial Policy Checklist.

Statistics

For all statistical analyses, confirm that the following items are present in the figure legend, table legend, main text, or Methods section.

n/a	Confirmed	
[ ]	[x]	The exact sample size (n) for each experimental group/condition, given as a discrete number and unit of measurement
[ ]	[x]	A statement on whether measurements were taken from distinct samples or whether the same sample was measured repeatedly
[ ]	[x]	The statistical test(s) used AND whether they are one- or two-sided<br>*Only common tests should be described solely by name; describe more complex techniques in the Methods section.*
[x]	[ ]	A description of all covariates tested
[ ]	[x]	A description of any assumptions or corrections, such as tests of normality and adjustment for multiple comparisons
[ ]	[x]	A full description of the statistical parameters including central tendency (e.g. means) or other basic estimates (e.g. regression coefficient) AND variation (e.g. standard deviation) or associated estimates of uncertainty (e.g. confidence intervals)
[ ]	[x]	For null hypothesis testing, the test statistic (e.g. F, t, r) with confidence intervals, effect sizes, degrees of freedom and P value noted<br>*Give P values as exact values whenever suitable.*
[x]	[ ]	For Bayesian analysis, information on the choice of priors and Markov chain Monte Carlo settings
[x]	[ ]	For hierarchical and complex designs, identification of the appropriate level for tests and full reporting of outcomes
[x]	[ ]	Estimates of effect sizes (e.g. Cohen's d, Pearson's r), indicating how they were calculated

Our web collection on statistics for biologists contains articles on many of the points above.

Software and code

Policy information about availability of computer code

Data collection	Agilent BioTek Gen5, GeneMapper Software (version 6.1), Image Studio software (version 6.1.0.79), Leica LASX (4.8.2.29567), LightCycler 480 Software (1.5.62 SP3), NIS-Elements AR (v5.42), Quantstudio software, Zeiss AxioVision imaging software (version 4.8)
Data analysis	edgeR (v3.42.4), FIJI (with cell counter plug-in; version 20240605-1417), GeneMapper Software (version 6.1), GPS-CCD (v1.0), GraphPad Prism (v10.6.1), Integrative Genome Viewer (v2.15.4), Image Studio software (version 6.1), InteractiVenn, MaxQuant software package (v2.2.0.0) with integrated Andromeda search engine, Microsoft Excel (Office 16), ngs-bits, Perseus software package (v1.6.2.3), QIAGEN IPA (version 24.0.2), R (v4.2.276; ggplot2 package), STAR (v2.7.10b70), STRING (v12), Subread (v2.0.4). Custom Fiji macros developed for the analysis of microscopy images are provided as Supplementary Software and have been deposited at Zenodo (https://doi.org/10.5281/zenodo.22231266).

For manuscripts utilizing custom algorithms or software that are central to the research but not yet described in published literature, software must be made available to editors and reviewers. We strongly encourage code deposition in a community repository (e.g. GitHub). See the Nature Portfolio guidelines for submitting code & software for further information.

Data

Policy information about availability of data

All manuscripts must include a data availability statement. This statement should provide the following information, where applicable:

- Accession codes, unique identifiers, or web links for publicly available datasets
- A description of any restrictions on data availability
- For clinical datasets or third party data, please ensure that the statement adheres to our policy

All data required to support the findings of this study are available within the article, the supplementary information, or are available in publicly available data repositories. The RNA sequencing data generated in this study have been deposited in the NCBI Gene Expression Omnibus (GEO) database under accession code GSE346419 [https://www.ncbi.nlm.nih.gov/geo/query/acc.cgi?acc=GSE346419]. The mass spectrometry proteomics data generated in this study have been deposited to the ProteomeXchange Consortium via the PRIDE101 partner repository under accession code PXD083710 [https://www.ebi.ac.uk/pride/archive/projects/PXD083710]. Source Data are provided with this paper. Additional information can be provided upon request.

Research involving human participants, their data, or biological material

Policy information about studies with human participants or human data. See also policy information about sex, gender (identity/presentation), and sexual orientation and race, ethnicity and racism.

Reporting on sex and gender	Sex was not considered as a biological variable in this study and is therefore not reported. The SCA17 patient-derived fibroblast and iPSC lines, as well as the derived neurons, were analysed alongside age- and sex-matched control lines from an unaffected individual.
Reporting on race, ethnicity, or other socially relevant groupings	Race, ethnicity, or other socially relevant groupings were not considered relevant to this study and are therefore not reported. No research on human populations was conducted.
Population characteristics	See above.
Recruitment	Biological material was obtained from one individual with SCA17 and one age- and sex-matched unaffected control individual. The individual with SCA17 was receiving genetic counselling at the Department of Human Genetics, Ruhr University Bochum, Bochum, Germany, and voluntarily participated in the study upon request. The control individual was recruited as an unaffected, age- and sex-matched comparator. Written informed consent was obtained from both individuals. Given the limited sample size and the voluntary nature of participation, selection bias cannot be excluded. The use of age- and sex-matched individuals was intended to minimise potential confounding by these variables; however, findings from these patient-derived samples should be interpreted with caution and cannot be considered representative of the broader SCA17 population.
Ethics oversight	This study was approved by the Ethics Committee of the University of Tübingen. All procedures involving human participants were conducted in accordance with the principles of the Declaration of Helsinki. Informed written consent was obtained from the donor.

Note that full information on the approval of the study protocol must also be provided in the manuscript.

Field-specific reporting

Please select the one below that is the best fit for your research. If you are not sure, read the appropriate sections before making your selection.

☒ Life sciences ☐ Behavioural & social sciences ☐ Ecological, evolutionary & environmental sciences

For a reference copy of the document with all sections, see nature.com/documents/nr-reporting-summary-flat.pdf

Life sciences study design

All studies must disclose on these points even when the disclosure is negative.

Sample size	Sample sizes were not determined a priori by statistical methods. For functional analyses, we selected sample sizes in accordance with established practice in the field, guided by prior studies and the known variability of the respective assays. For zebrafish experiments, clutches of fertilized eggs were equally distributed into the different treatment groups. All experiments were done repeatedly and scored by different researchers.
Data exclusions	Only statistical outliers in cell culture-based analysis were excluded, based on ROUT test (Q = 1%). No data were excluded from rat tissue- and zebrafish-based analyses.
Replication	All experiments done in cell models, rat tissue, and zebrafish were done repeatedly and results could be successfully reproduced.
Randomization	No specific randomization was performed for the cell-based experiments or for analyses of available rat tissues, as these experiments did not involve prospective allocation of individual animals or samples into experimental groups. In zebrafish experiments, fertilized eggs from a single clutch were randomly distributed among the different treatment groups within each experiment.
Blinding	Whenever possible, data were analysed by more than one scientist. For the zebrafish experiments, blinding was not possible because the

phenotypes were readily apparent. For all other experiments, the study design and experimental procedures did not involve conditions in which investigator knowledge of group allocation could influence data acquisition or analysis. Therefore, blinding was not undertaken for these experiments.

Reporting for specific materials, systems and methods

We require information from authors about some types of materials, experimental systems and methods used in many studies. Here, indicate whether each material, system or method listed is relevant to your study. If you are not sure if a list item applies to your research, read the appropriate section before selecting a response.

Materials & experimental systems

n/a	Involved in the study
☐	☒ Antibodies
☐	☒ Eukaryotic cell lines
☒	☐ Palaeontology and archaeology
☐	☒ Animals and other organisms
☒	☐ Clinical data
☒	☐ Dual use research of concern
☒	☐ Plants

Methods

n/a	Involved in the study
☒	☐ ChIP-seq
☒	☐ Flow cytometry
☒	☐ MRI-based neuroimaging

Antibodies

Antibodies used

Primary:
αII-spectrin (mouse, AA6/MAB1622, Merck), α-tubulin (mouse, DM1A/CP06, Merck), α-tubulin (rabbit, 11224-1-AP, Proteintech), AMOT (mouse, sc-166924, Santa Cruz Biotechnology), AMOT (mouse, sc-517353, Santa Cruz Biotechnology), AMOT (rabbit, ABS1024, Merck), AMOT (rabbit, 16870-1-AP, Proteintech), AMOT (rabbit, 24550-1-AP, Proteintech), ataxin-3 (ATXN3) (mouse, 1H9/MAB5360, Merck), ataxin-3 (ATXN3) (mouse, 1H9-2/650401, BioLegend), β-actin (mouse, AC-15/A5441, Sigma-Aldrich), βIII-tubulin (mouse, SDL.3D10/T8660, Merck), calpain-1 (CAPN1) (rabbit, ab39170, Abcam), calpain-1 (CAPN1) (rabbit, 10538-1-AP, Proteintech), calpain-2 (CAPN2) (mouse, 1E1F10/66977-1-Ig, Proteintech), calpain-5 (CAPN5) (rabbit, ab38943, Abcam), calpain-7 (CAPN7) (rabbit, ab28211, Abcam) ,calpain-10 (CAPN10) (rabbit, ab28220, Abcam), calpain-15 (CAPN15) (rabbit, 33924-1-AP, Proteintech), calpain small subunit 1 (CAPNS1) (mouse, P1/MAB3083, Merck), calpastatin (CAST) (rabbit, #4146, Cell Signaling Technology), calpastatin (CAST) (rabbit, sc-20779, Santa Cruz Biotechnology), CTGF/CCN2 (rabbit, 25474-1-AP, Proteintech), CTIP2 (rat, 25B6/ab18465, Abcam), CYR61/CCN1 (rabbit, 26689-1-AP, Proteintech), GAPDH (mouse, 0411/sc-47724, Santa Cruz Biotechnology), GAPDH (rabbit, 10494-1-AP, Proteintech), GFP (mouse, clone 3E6, A-11120, Thermo Fisher Scientific), Lamin A/C (mouse, E-1/sc-376248, Santa Cruz Biotechnology), Lamin A/C (rabbit, H-110/sc-20681, Santa Cruz Biotechnology), MAP1LC3B (LC3B) (rabbit, #2775, Cell Signaling Technology), MAP2 (chicken, Poly28225/822501, BioLegend), Myelin basic protein (MBP) (rabbit, ab40390, Abcam), myc tag (mouse, 9B11/#2276, Cell Signaling Technology), myc tag (rabbit, 71D10/#2278, Cell Signaling Technology), polyglutamine (polyQ) (mouse, 5TF1-1C2/MAB1574, Merck), SQSTM1/p62 (p62) (rabbit, #5114, Cell Signaling Technology), TBP (mouse, 1TBP18/sc-56794, Santa Cruz Biotechnology), TBP (mouse, 58C9/T1827, Sigma-Aldrich), TBP (mouse, 58C9/sc-421, Santa Cruz Biotechnology), TBP (rabbit, #8515, Cell Signaling Technology), TBP (rabbit, D5C9H/#44059, Cell Signaling Technology), TBP (rabbit, D5G7Y/#12578, Cell Signaling Technology), TBR1 (rabbit, EPR8138(2)/ab183032, Abcam), ubiquitin (K48-linkage specific) (rabbit, D9D5/#8081, Cell Signaling Technology), ubiquitin (K48-linkage specific) (rabbit, APU2/ZRB2150, Sigma Aldrich), V5 tag (mouse, SV5-Pk1, Invitrogen), Vinculin (VCL) (rabbit, E1E9V/#13901, Cell Signaling Technology), YAP1 (rabbit, 13584-1-AP, Proteintech)

Secondary:
goat anti-mouse Alexa Fluor 488 (#4408, Cell Signaling Technology), donkey anti-mouse Alexa Fluor 568 (A10037; Thermo Fisher Scientific), goat anti-rabbit Alexa Fluor 488 (A27034, Thermo Fisher Scientific), goat anti-mouse Alexa Fluor 488 (A32723, Thermo Fisher Scientific), goat anti-mouse Alexa Fluor 555 (A-21424, Thermo Fisher Scientific), goat anti-rabbit Alexa Fluor 594 (A-11037, Thermo Fisher Scientific), goat anti-mouse Alexa Fluor 594 (A32742, Thermo Fisher Scientific), goat anti-rat Alexa Fluor 594 (A-11007, Thermo Fisher Scientific), goat anti-chicken Alexa Fluor 647 (A32933, Thermo Fisher Scientific), goat anti-mouse 680LT (P/N 926-68020, LI-COR Biosciences), goat anti-mouse 800CW (P/N 926-32210, LI-COR Biosciences), goat anti-rabbit 800CW (P/N 926-32211, LI-COR Biosciences), goat anti-mouse HRP (#A28177; Thermo Fisher Scientific), donkey anti-mouse HRP (715-035-150; Jackson ImmunoResearch Europe Ltd, Cambridgeshire, UK), goat anti-rabbit HRP (#G-21234; Thermo Fisher Scientific)

Validation

All antibodies were purchased from their respective manufacturers and validation was mainly based on available antibody profiles and validation statements. Primary antibodies crucial for the study were additionally validated in our laboratory using gene knock-in, knock-out or overexpression cell and animal models (including but not limited to antibodies directed against AMOT, ATXN3, CAPN1, CAPN2, CAST, polyglutamine, YAP1, and TBP).

Eukaryotic cell lines

Policy information about cell lines and Sex and Gender in Research

Cell line source(s)

HEK 293T (293T) (ATCC: CRL-3216),
SH-SY5Y (SY5Y) (ATCC: CRL-2266),
primary human fibroblasts, iPSCs, and IPSC-derived iCNs were generated in-house.

Authentication	All purchased cell lines (ATCC) were further tested in-house by the respective pipeline of our cytogenetics department for authentication.
Mycoplasma contamination	All cell lines were tested regularly for mycoplasma contamination and confirmed mycoplasma-negati
Commonly misidentified lines (See ICLAC register)	No commonly misidentified cell lines were used in this study.

Animals and other research organisms

Policy information about studies involving animals; ARRIVE guidelines recommended for reporting animal research, and Sex and Gender in Research

Laboratory animals	SCA17 rats (Rattus norvegicus): wild-type and TBPQ64 8.4 (of the Sprague Dawley strain), 10 months; Zebrafish (Danio rerio): EK and AB wild-type strains, tg(isl1:gfp). Only embryos younger than 5 days post fertilization were used.
Wild animals	The study did not involve wild animals.
Reporting on sex	Tissues from SCA17 rats derived all from male individuals. Based on their first characterization (Kelp et al. 2012) both sexes develop a comparable phenotype. The sex itself was not considered in the study design and should not influence the measured parameters. No sex-based analyses were performed as zebrafish embryos used in this study had not reached the stage when their biological sex would develop. Zebrafish embryos younger than 8-14 days post fertilization are hermaphroditic and could develop into either male or female fish.
Field-collected samples	The study did not involve samples collected from the field.
Ethics oversight	All experiments involving rats were conducted after approval by the local ethics committee at the Regierungspräsidium Tübingen, in accordance with the German Animal Welfare Act and the guidelines of the Federation of European Laboratory Animal Science Associations, based on European Union legislation (Directive 2010/63/EU).<br>Zebrafish husbandry and maintenance were approved by local authorities (Veterinary Care Unit at the University of Tübingen and the animal welfare commissioner of the regional board for scientific animal experiments in Tübingen, Germany). Experiments were conducted in agreement with the European Union Directive 86/609/EEC for the protection of animals used for experimental and other scientific purposes.

Note that full information on the approval of the study protocol must also be provided in the manuscript.

Plants

Seed stocks	No plants were used in this study.
Novel plant genotypes	See above.
Authentication	See above.
