# Supplementary material for: Calpain-mediated TBP cleavage disturbs neuronal AMOT-YAP1 signalling
Source: Nat Commun. 2026 Nov 9;17:11531. doi: 10.1038/s41467-026-79055-x (PMC13748621; doi:10.1038/s41467-026-79055-x)
Supplement: Supplementary file 4 [file 41467_2026_79055_MOESM9_ESM.pdf]

nature portfolio

Peer Review File

# Calpain-mediated TBP cleavage disturbs neuronal AMOT-YAP1 signalling

Corresponding Author: Dr Jonasz Weber

**This file contains all reviewer reports in order by version, followed by all author rebuttals in order by version.**

Version 0:

Reviewer comments:

Reviewer #1

(Remarks to the Author)
Review for “Calpain-mediated TBP cleavage”
This study by Eltemur et al. explores the role of calpain-mediated cleavage of TATA-binding protein (TBP), which is implicated in spinocerebellar ataxia type 17 (SCA-17) when its polyglutamine (polyQ) region expands beyond 42 repeats. Using in vitro enzyme cleavage assays and proteomics, the study identifies T106 as a key cleavage site. The resulting N-terminal fragments mislocalize to the cytoplasm and aggregate, while the C-terminal fragments remain in the nucleus. Multi-omics analyses reveal a dysregulation of neuronal pathways, particularly involving AMOT and YAP1 signaling. The study demonstrates that AMOT depletion, caused by TBP fragmentation, disrupts neuronal function and motor development in zebrafish, linking these findings to early SCA-17 pathogenesis. This work provides novel insights into the mechanisms behind TBP fragmentation and its downstream effects. The experiments are well-executed, with organized data presentations.
Overall, this is a robust and significant contribution to understanding the effects of TBP fragmentation in the pathogenesis of SCA-17, which may have implications for other polyglutamine disorders. The combination of molecular, cellular, and in vivo approaches strengthens the conclusions.
However, to enhance the clarity and impact of the study, several points need to be addressed. First, please discuss the discrepancy with findings from the same group (Cellular and Molecular Life Sciences, 2022, 79:262), which shows that the TBP C-terminus is localized in the cytoplasm, differing from the current study. An explanation for this discrepancy is warranted. Second, as shown by the author, the TBP and Calpain are not localized in the same cellular compartment. The calpain is present in the cytoplasm while TBP resides in the nucleus, please speculate on how cytoplasmic calpain is able to cleave nuclear TBP. Lastly, validating TBP fragmentation in SCA-17 human cells (such as fibroblasts or induced pluripotent stem cells) would enhance the study's impact.
Some minor suggestions: Please use common terminology when describing equipment, such as referring to the Axioplan 2 as a fluorescent microscope, as the term "epi-fluorescent" may be confusing. Additionally, in Figure 6h, "fibroblast" should be capitalized as "Fibroblast."

Reviewer #2

(Remarks to the Author)
The manuscript by Eltemur has provided further examination of calpain-mediated cleavage of TBP, the protein affected in SCA17. The authors report for the first time a calpain cleavage site at T106-S107 within TBP. The authors provide analysis of samples from immortal cell cultures, SCA17 patient fibroblasts, SCA17 transgenic rats and zebrafish. The key findings from this study include confirmation of the role of calpains in TBP cleavage, the influence of these TBP fragments on cell toxicity and inclusion body formation and suggestion of AMOT as a key player in calpain overactivity. The SCA17 field and calpain activity field will likely have interest in the findings.
Overall, the manuscript presents interesting findings. However, some findings, such as the presentation and quantification of various cleavage fragments, inclusion bodies and the zebrafish findings need further consideration and strengthening. Finally, some findings are overstated and require further evidence of support.

Major points:

1. A major component of this study is the finding that ‘angiomotin (AMOT) levels were significantly reduced in all fragment-expressing conditions (TBP1-106 38Q, TBP1-106 106Q, and TBP107-339), exhibiting the most pronounced fold changes (Fig. 6e, f) (line 233)’. Firstly, this sentence needs tweaking as it isn’t clear whether the sentence is saying that AMOT was reduced in all samples, with fragment-expressing conditions exhibiting the most pronounced fold changes, or instead, which group is it saying has the most profound changes? A prior line (line 218 ) describes “increased calpain activation in cells expressing polyQ-expanded full-length TBP and N-terminal TBP fragment”. Whilst that wording also needs clarifying (N-terminal TBP fragments also including 38Q or only 106Q?), Figure 6c-d shows that increased calpain activation is present in TBP 106Q, TBP1-106 106Q, and TBP107-339, but not TBP 38Q or TBP1-106 38Q. In comparison, AMOT levels were significantly reduced even in TBP1-106 38Q cells, which were not presenting with increased calpain activity. Further, whilst TBP107-339 shows signs of the greatest level of calpain overactivity, it has the smallest change in AMOT levels. With these findings in mind, the proposed role of decreased AMOT levels in calpain overactivity needs further justification.

2. In Figure 6H, the authors used SCA17 patient fibroblasts to validate AMOT proteomic findings. It would be interesting to confirm whether levels of calpain 1, calpastatin or cleaved spectrin from SCA17 patient fibroblasts aligned with findings shown in Figure 6C. If the authors have previously examined calpain-activity in SCA17 fibroblasts, they should direct the reader to their previous work within the text, otherwise they should add these additional experiments within this study. Further, it is unknown whether the c-terminal or n-terminal TBP fragments (~30-20kDa, described in Figures 1 and 2) are present in SCA17 patient fibroblasts, and whether the T106 and S107 TBP cleavage site was validated in SCA17 patient fibroblasts. If TBP cleavage fragments of a similar size are observed in SCA17 patient fibroblasts, this should be reported and the authors should comment on the clinical relevance of the findings in Figures 1 and 2 to strengthen their findings. If these cleavage fragments were not observed in SCA17 fibroblasts, the authors should provide a possible explanation for their absence.

3. In Figure 5 the authors describe a relationship between expression of short TBP fragments and decreased AMOT levels but in Figure 6, decreased AMOT levels are observed in SCA17 patient fibroblasts without any comment on the possible associated with TBP fragmentation. Abundance of TBP cleavage fragments need to be quantified in the SCA17 patient fibroblasts. If these fragments are not present and therefore AMOT is not recruited to the short TBP fragment inclusion bodies, what is the mechanism for reduced AMOT levels in these cells? Further, the total amount of TBP (full length and cleavage together) should be quantified in these samples, as it appears to be lower than in the control individual fibroblasts.

4. In cases where amount of protein cleavage such as calpastatin cleavage is being examined, the cleavage fragment, not just the full length band, should be shown and quantified.

5. The authors performed AMOT knockdown experiments in two cell lines; HEK293T (consistent with other data from this paper) and SHSY5Y cells as a 'neuronal model.' A more useful neuronal model for investigation would be neurons reprogrammed from the SCA17 patient fibroblasts.

Further, the reduction in AMOT levels in SHSY5Y cells (Figure 7G) is relatively modest compared to the knockdown effect in HEK293T cells (Figure 7F). Do the authors have any hypotheses as to why the knockdown seems to have been less effective in their 'neuronal' cell line?

6. The size of the “TBP inclusions” shown within Figure 5 are very large. Additional imaging, including z-stack projections, are required.

7. On page 10, the authors conclude: "Additionally, we explored whether AMOT levels declined due to its recruitment into IBs formed by polyQ-expanded TBP or N-terminal TBP fragments. Epi-fluorescence microscopy revealed that AMOT colocalised with IBs formed by N-terminal TBP fragments (Supplementary Fig. 4d)." Does AMOT form inclusions that were not TBP-positive, i.e is AMOT alone able to form inclusions or are 100% of these structures co-localised with TBP inclusions? Secondly, do the authors know if full-length or cleaved AMOT is being recruited to the inclusion bodies? If there are AMOT inclusion bodies that are not TBP positive, where are these inclusions located (nuclear or cytoplasmic localisation?)

8. In the discussion (page 14, line 405) the authors state "The presence of an N-terminal polyQ stretch in AMOT may underlie its co-aggregation into TBP-positive IBs." The authors have not previously stated that AMOT contains a polyQ stretch. This concept seems highly relevant to the key story of the manuscript and should be addressed earlier. Further, the relevance of the polyQ stretch within AMOT should also be mentioned earlier when AMOT is described as a calpain substrate.

9. In Figure 4C, the authors display a western blot demonstrating changes in K48 polyubiquitin chains, p62 or LC3 but there is no quantification to accompany the western blot shown in Figure 4C.

10. In Figure 8b, the authors describe differences in AMOT expression in zebrafish expressing TBP 38Q and 106Q. However, an older age, such as 27hpf or 72hpf should be examined so that the findings are not purely early developmental.

11. It needs to be stated that AMOT knockout in the CRISPR/Cas9 zebrafish is mosaic as F0 embryos are being examined. This examination of the injected animals will have potential effects of off target effects of the injection. Whilst control animals have also received cas9 injection, they have not received any guide RNA so the off target and injection related effects may be less. Examination of offspring from future generations would be more informative.

12. To obtain insights relating to the neuronal numbers, dopamine neuron numbers and tail coiling, the developmental stage of the animals examined needs to be confirmed and controlled for, such as through body length measurement.

13. The various zebrafish phenotypes should be rescued through coinjection of human AMOT mRNA to prove that the reported phenotypes are AMOT dependent rather than off target effects.

14. TBP and spectrin protein cleavage should be examined in the AMOT knockout zebrafish, to demonstrate the altered processing in that model.

15. The zebrafish findings put forward decreased motor neurons in the AMOT knockout zebrafish, but increased dopamine neuron numbers, increased aberrant motor neuron axons, increased spontaneous coiling, but decreased touch evoked responses. This combinations of phenotypes needs describing and discussion.

16. Line 416 states that “To investigate the neuronal role of AMOT in vivo, we genetically depleted amot in zebrafish embryos. This resulted in disrupted YAP1-associated Hippo signalling, impaired motor neuron development, and reduced motor function.” This is an overstatement. Disrupted YAP1-associated Hippo signalling is not demonstrated in the AMOT knockout zebrafish, two transcripts are shown to be present at altered abundance, but signalling disruption is not demonstrated. “reduced motor function” is mentioned, but increased spontaneous coiling is presented, so that statement needs rewording.

17. On page 8, lines 205-207 the authors state: "Conversely, the C206 terminal fragment TBP107-339, which contains the DNA-binding core domain, was primarily found in the nuclear fraction (Fig. 5g)." The authors should consider altering the working of this statement, as the inference from this statement is that expression is very low within the cytoplasm however the western blot image shown in Figure 5G shows the c-terminal fragment band is clearly visible within the cytoplasmic fraction, albeit at a relatively lower density. Perhaps a more suitable description would that this c-terminal fragment is found in both the cytoplasm and nucleus, however expression of the c-terminal fragment appears to be stronger in the nuclear fraction.

Minor points:
1. AMOT knockout in the zebrafish is shown in Supplementary Figure, this should be shown in Figure 6 instead, it is required within this study.
2. The schematic in Figure 3C shows 9 indicators of CAPN1 or chymotrypsin cleavage and 13 different amino acid sequences produced by this cleavage. The in silico results need further explanation to clearly explaining how the 10 peptides were identified, with further information including 1) how many cleavage sites were identified in total using the GPS-CCD tool, 2) which proteases were predicted to cleave at these sites, 3) how many sites were predicted to be calpain cleavage sites and 4) how did this compare to your in vitro calpain 1 data.
3. In Figure 5, the authors observed structural differences in inclusion body formation following expression of full-length TBP 106Q compared with TBP fragments 1-10638Q and 1-106106Q. In Supplementary Figure 4, it is shown that AMOT is recruited to the IBs produced by shorter TBP fragments and that this could explain the differences in localisation (nuclear vs perinuclear) and increased IB size. Another factor that could contribute, could be the presence or absence of NES or NLS sequences. Do the authors know whether there are NES or NLS signals within TBP and how the presence or absence of these signals could affect fragment localisation?
4. The authors state on line 149, page 7 "... a primary calpain-1 cleavage event takes place between T106 and S107." To provide further conclusive experimental evidence, the authors could perform a site directed mutagenesis and demonstrate absence of calpain cleavage following mutation of the calpain cleavage site.
5. In Figure 8J, the authors describe the effects of Amot knockdown on dopaminergic neurons. Why were dopaminergic neurons examined? What is the link between dopaminergic neurons and Amot? This rationale should be more clearly explained.
6. The findings presented in Supplementary Figures 4 and 5 are interesting. One interpretation is that polyQ containing proteins (either full-length or shorter fragments) recruit AMOT to inclusion bodies, leading to decreased soluble levels of AMOT. Have the authors examined whether AMOT colocalises with TBP107-339 which does not contain a polyQ stretch?
7. In the discussion, the authors mention that non-expanded TBP promotes AMOT levels. This point should be clarified as the current manuscript does not provide experimental evidence as to whether TBP 38Q increases or promotes AMOT level, i.e. does overexpression of TBP 38Q increase AMOT levels above endogenous AMOT levels?
8. No quantification of the data presented in Supplementary Figure 4A. Quantification of these westerns is needed to support the other findings within this figure.
9. The conclusion from Supplementary Figure 4 is that AMOT is a calpain substrate. To support the findings in Supplementary Figure 4, the authors should discuss whether the protein sequence of AMOT contains any calpain cleavage motifs.
10. In Supplementary Figure 3 and Figure 7, naming of experimental conditions alternates between TBP1-106 38Q or 38QF, TBP1-106 106Q or 106QF, and TBP107-339 or CF. For clarity, I would suggest keeping the naming of experimental conditions consistent with the bulk of the figures, i.e. TBP1-106 38Q, TBP1-106 106Q and TBP107-339.

Reviewer #3

(Remarks to the Author)
The manuscript “Calpain-mediated TBP cleavage disturbs neuronal AMOT-YAP1 2 signaling” is an interesting paper that shows that transcription factor TBP is cleaved by calpains, the site of cleavage is after residue T106, and the N-terminal fragment localizes to the cytoplasm. TBP is an essential transcription factor, and polyglutamine expansion in this TF causes SCA17. Proteomics analysis links the TBP fragments to AMOT and further analysis shows nice correlation with transcriptional changes. The work is rigorous, and the figures are well-constructed and informative. A deeper analysis of the

rat SCA17 model to validate their findings would strengthen the research.
Points to consider for revision are:
Point 1. The abstract requires a few more specific details. For example, “Multiomics analysis of what? SCA17 cells, SCA17 mice and zebrafish…
Point 2. Remove the word “Beyond transcription, ….. This does not make sense as the transcriptional activity is what is crucial for neurological function.
Point 3. “Despite its discovery over two decades ago, the molecular pathomechanisms driving selective neuronal loss in SCA17 remain unclear”. I don’t think the author’s current paper addresses selective neuronal loss, so maybe remove the word selective.
Point 4. The references are not cited appropriately and should be the references to the first studies. Under the toxic fragment hypothesis, proteolytic cleavage of disease-linked proteins is a key event in neurodegeneration, as seen in AD, PD, and polyQ disorders such as Huntington’s disease (HD), and SCA3, the following key references.
Point 5. Figure 1. Please state in the legend the cell type the TBP was immunopurified. Also, in panel 1d, the second western blot from left, the three bands (n1, n2 and n3) are not clear in the 38Q IP. Perhaps a darker exposure.
Point 6. The paper focus on calpain-1 which they state is predominantly localized to the cytoplasm. Other calpain family members have nuclear localization and some work on polyQ diseases addresses this. This is important because the model that the authors suggest is that TBP fragmentation by calpain-1 occurs in the cytoplasm, followed by the nuclear translocation of TBP fragments c1 and c2. It is likely that other calpain family members can cleave TBP in the nucleus and the model they generate is very artificial with overexpression. Calpain-1 overexpression will activate these enzymes.
Point 7. The conclusion for Figure 4 is reasonable “our findings indicate that N- and C-terminal TBP fragments derived from calpain cleavage at T106|S107 exhibit reduced protein levels. This reduction is attributed to high proteolytic turnover via the proteasomal pathway for the C-terminal fragment, while the N-terminal polyQ-containing fragments, particularly polyQ-expanded TBP1-106 106Q, demonstrate a high degree of aggregation”.
Point 8. It makes sense from the proteomics to evaluate AMOT but when it is introduced in the text it would be helpful to the reader to explain what it does functionally. Also, the authors do not do enrichment analysis of the proteome altered by the TBP1-106 106Q fragment, nor compare their results to more human-relevant models like fibroblasts or the proteome of the TBP polyQ expansion rat, which would strengthen its significance.
Point 8. The analysis of the SCA17 rat model is rather limited for the paper. It would be reasonable to see the generation of the relevant calpain fragment in this model, an neoepitope antibody to the cleavage product, western blot and IHC of the key players in this paper for validation of the mechanism. Generally the paper focuses on mechanistic studies in 293T cells and this may not be relevant to SCA17 and needs further validation.

Reviewer #4

(Remarks to the Author)
I co-reviewed this manuscript with one of the reviewers who provided the listed reports. This is part of the Nature Communications initiative to facilitate training in peer review and to provide appropriate recognition for Early Career Researchers who co-review manuscripts.

Version 1:

Reviewer comments:

Reviewer #1

(Remarks to the Author)
Review for NCOMMS-25-39340A
Calpain-mediated TBP cleavage disturbs neuronal AMOT-YAP1 signalling
This is a revised manuscript, I appreciate the authors’ efforts to perform additional human cell experiment and added discussions to clarify the current study discrepancy with their previous work.
I have carefully read the revised manuscript and still have some concerns that need the author to address to enhance the manuscript’s strength.
Figure 8, This is a new figure showing that the SCA-17 iPSC also showed TBP fragmentation and dysregulated AMOT. While it is a plus using patient cells, the immunofluorescent staining of AMOT and the western blot of AMOT seem not correlate well. It looks like the western blot shows AMOT in SCA-17 iPSC is significantly decreased, however, the immunostaining of the cells does not look much different compared to the control iPSC, except the cell body may look smaller. The AMOT staining in Figure S12 also has the same problem. The authors might need to use a different antibody or use a different antibody dilution for the immunostaining of the cells. The control and SCA17 patient iPSC should indicate the polyQ number.
Minor:
Supplement S5 Legend does not have a description for figure “a “. Need to add it for less confusion.
The control and SCA17 patient iPSC should indicate the polyQ number.

Reviewer #2

(Remarks to the Author)
The manuscript is greatly improved.

All provided feedback has been responded to.

Inclusion of induced cortical neurons in addition to the SCA17 patient fibroblast findings, and expansion of those findings to cover additional components that had been requested is a great improvement and appreciated.

The authors denote the generated cells as cortical neurons, but the only validation provided is MAP2 staining. Because MAP2 is a pan-neuronal marker, it is insufficient to confirm regional identity, functional subtype, or culture purity. To scientifically support the claim that these are indeed cortical neurons, the manuscript would require inclusion of specific validation markers (e.g., PAX6, FOXG1, TBR1, and vGLUT1) via immunocytochemistry or RT-qPCR.

Additional requested rescue experiments and validation experiments within the zebrafish findings are well received (especially supp Figure 17e-g).

Some minor points:
*It looks like Supplementary Figure 12d (in figure legend) should be g.
*Within response 5) within rebuttal document a new Supplementary Figure 8e-f is described, but this cannot be found in the figures. It is likely Supp Figure 12e-g, which is fine.

Reviewer #3

(Remarks to the Author)
I am satified with the careful responses to my review and the revised experiments and edits to the manuscript.

**Open Access** This Peer Review File is licensed under a Creative Commons Attribution 4.0 International License, which permits use, sharing, adaptation, distribution and reproduction in any medium or format, as long as you give appropriate credit to the original author(s) and the source, provide a link to the Creative Commons license, and indicate if changes were made.
In cases where reviewers are anonymous, credit should be given to 'Anonymous Referee' and the source.
The images or other third party material in this Peer Review File are included in the article’s Creative Commons license, unless indicated otherwise in a credit line to the material. If material is not included in the article’s Creative Commons license and your intended use is not permitted by statutory regulation or exceeds the permitted use, you will need to obtain permission directly from the copyright holder.
To view a copy of this license, visit https://creativecommons.org/licenses/by/4.0/

**RESPONSE TO REVIEWER COMMENTS**

Reviewer #1 (Remarks to the Author):

Review for "Calpain-mediated TBP cleavage"

This study by Eltemur et al. explores the role of calpain-mediated cleavage of TATA-binding protein (TBP), which is implicated in spinocerebellar ataxia type 17 (SCA-17) when its polyglutamine (polyQ) region expands beyond 42 repeats. Using in vitro enzyme cleavage assays and proteomics, the study identifies T106 as a key cleavage site. The resulting N-terminal fragments mislocalize to the cytoplasm and aggregate, while the C-terminal fragments remain in the nucleus. Multi-omics analyses reveal a dysregulation of neuronal pathways, particularly involving AMOT and YAP1 signaling. The study demonstrates that AMOT depletion, caused by TBP fragmentation, disrupts neuronal function and motor development in zebrafish, linking these findings to early SCA-17 pathogenesis. This work provides novel insights into the mechanisms behind TBP fragmentation and its downstream effects. The experiments are well-executed, with organized data presentations.

Overall, this is a robust and significant contribution to understanding the effects of TBP fragmentation in the pathogenesis of SCA-17, which may have implications for other polyglutamine disorders. The combination of molecular, cellular, and in vivo approaches strengthens the conclusions.

**Response:** We are grateful to the Reviewer for their positive evaluation of our work, for recognizing the relevance of our findings, and for providing us with the opportunity to revise the manuscript.

However, to enhance the clarity and impact of the study, several points need to be addressed.

First, please discuss the discrepancy with findings from the same group (Cellular and Molecular Life Sciences, 2022, 79:262), which shows that the TBP C-terminus is localized in the cytoplasm, differing from the current study. An explanation for this discrepancy is warranted.

**Response:** We thank the Reviewer for this important observation and question. The discrepancy arose from limitations in the study design of our previous work. In the fractionation assays presented in our earlier publication, all observed fragments were initially interpreted as calpain-dependent. However, our subsequent analyses revealed that some fragments originated independently of calpain activity. Moreover, additional non-specific bands migrating at molecular weights comparable to the expected fragments contributed to their misidentification. Inclusion of calpain-specific controls, such as calpain inhibition or overexpression, in the earlier study would have clarified this issue. To address and explain this point, we have modified and extended the following sentence in the Discussion section:

> "C-terminal TBP fragments containing the DNA-binding core domain localised to the nucleus – contrary to our earlier findings[28], which may be explained by a misidentification of the previously observed fragments in cytoplasmic fractions due to

> the lack of calpain-specific control conditions. However, the current observation is consistent with predicted C-terminal nuclear localisation signals[15] and previously reported karyopherin-mediated nuclear shuttling in yeast, which depends on a recognition motif located near the protein's C-terminal core domain[54, 55]."

Second, as shown by the author, the TBP and Calpain are not localized in the same cellular compartment. The calpain is present in the cytoplasm while TBP resides in the nucleus, please speculate on how cytoplasmic calpain is able to cleave nuclear TBP.

**Response:** The Reviewer's suggestion addresses an important aspect of calpain-mediated TBP fragmentation. In our previous study, we described a limited spatial overlap between TBP and calpains in the cytoplasm, due to the predominantly nuclear localization of TBP and the absence of calpain-1 and calpain-2 from the nucleus. We hypothesize that, following TBP translation in the cytoplasm, a short temporal and spatial window exists during which cytoplasmic calpains may cleave the protein before it is imported into the nucleus, thereby potentially modulating TBP nuclear import.

This mechanism may also explain the retention of the N-terminal, polyQ-containing fragment in the cytoplasm and the nuclear import of C-terminal TBP fragments, rather than requiring post-cleavage export of N-terminal TBP breakdown products, which would be unlikely given the absence of nuclear export signals in the TBP N-terminus.

In response to comments from Reviewer #3, we further confirmed by fluorescence microscopy that all calpains expressed in our cell model are localized to the cytoplasm. In addition, knockdown experiments demonstrated that calpain-1 represents the primary source of TBP cleavage, while *CAST* knockdown further enhanced formation, particularly of the c1 fragment. These results are shown in the revised and expanded Supplementary Figure 2c-f and the new Supplementary Figure 3. These observations are summarized in the final sentence of the second Results paragraph:

> "These results indicate that TBP fragmentation by calpains occurs in the cytoplasm, followed by the nuclear translocation of TBP fragments c1 and c2."

To further elaborate on this point, we have added the following statement to the Discussion:

> "In the present study, we confirmed our initial findings and demonstrate that calpain-dependent TBP cleavage – given the predominantly cytoplasmic localisation of these proteases – is a non-nuclear process that affects cytoplasmic TBP after translation but prior to its reported nuclear import[30]."

Lastly, validating TBP fragmentation in SCA-17 human cells (such as fibroblasts or induced pluripotent stem cells) would enhance the study's impact.

**Response:** We agree with the Reviewer that validation of TBP fragmentation in SCA17 patient-derived cells is essential to substantiate the physiological relevance of calpain involvement. We established a new collaboration with our colleagues from the German Center for

Neurodegenerative Diseases (DZNE, Tübingen) to generate iPS cells derived from our SCA17 fibroblast line and a sex- and age-matched control, which were differentiated into cortical neurons (iCNs). Using this model, we confirm baseline TBP fragmentation, which is elevated in SCA17 iCNs, and demonstrate enhanced calpain-dependent TBP cleavage upon ionomycin-induced activation. We further provide data (please, see new Figure 8) that calpains efficiently cleave TBP in SCA17 iCNs, resulting in fragment patterns mirroring those observed in our cell line-based system. We also demonstrate that calpains are overactivated in SCA17 iCNs and AMOT levels are reduced similar to our findings in 293T cells, SCA17 rat cerebellum and zebrafish embryos.

Some minor suggestions: Please use common terminology when describing equipment, such as referring to the Axioplan 2 as a fluorescent microscope, as the term "epi-fluorescent" may be confusing.

**Response:** We have revised the terminology used to describe our equipment.

Additionally, in Figure 6h, "fibroblast" should be capitalized as "Fibroblast."

**Response:** We thank the Reviewer for their careful reading of the manuscript. As we have consistently used lowercase labelling for non-gene/protein terms throughout our figures (e.g., “input”, “control”, “rat”, and “zebrafish”), we prefer to maintain the non-capitalized form of “fibroblasts” in these panels for consistency.

Reviewer #2 (Remarks to the Author):

The manuscript by Eltemur has provided further examination of calpain-mediated cleavage of TBP, the protein affected in SCA17. The authors report for the first time a calpain cleavage site at T106-S107 within TBP. The authors provide analysis of samples from immortal cell cultures, SCA17 patient fibroblasts, SCA17 transgenic rats and zebrafish. The key findings from this study include confirmation of the role of calpains in TBP cleavage, the influence of these TBP fragments on cell toxicity and inclusion body formation and suggestion of AMOT as a key player in calpain overactivity. The SCA17 field and calpain activity field will likely have interest in the findings.

Overall, the manuscript presents interesting findings. However, some findings, such as the presentation and quantification of various cleavage fragments, inclusion bodies and the zebrafish findings need further consideration and strengthening. Finally, some findings are overstated and require further evidence of support.

**Major points:**

1. A major component of this study is the finding that 'angiomotin (AMOT) levels were significantly reduced in all fragment-expressing conditions (TBP1-106 38Q, TBP1-106 106Q, and TBP107-339), exhibiting the most pronounced fold changes (Fig. 6e, f) (line 233)'. Firstly, this sentence needs tweaking as it isn't clear whether the sentence is saying that AMOT was reduced in all samples, with fragment-expressing conditions exhibiting the most pronounced fold changes, or instead, which group is it saying has the most profound changes? A prior line (line 218) describes "increased calpain activation in cells expressing polyQ-expanded full-length TBP and N-terminal TBP fragment". Whilst that wording also needs clarifying (N-terminal TBP fragments also including 38Q or only 106Q?), Figure 6c-d shows that increased calpain activation is present in TBP 106Q, TBP1-106 106Q, and TBP107-339, but not TBP 38Q or TBP1-106 38Q. In comparison, AMOT levels were significantly reduced even in TBP1-106 38Q cells, which were not presenting with increased calpain activity. Further, whilst TBP107-339 shows signs of the greatest level of calpain overactivity, it has the smallest change in AMOT levels. With these findings in mind, the proposed role of decreased AMOT levels in calpain overactivity needs further justification.

**Response:** We thank the reviewer for this detailed and insightful comment and agree that clarification of the respective statements is necessary. We have therefore revised the text, also incorporating suggestions from Reviewer #3, as follows:

> "Western blot analysis of calpain markers α-spectrin, calpastatin, and calpain-1 revealed increased calpain activation in cells expressing full-length TBP 106Q and the N-terminal fragment $TBP_{1\text{-}106}$ 106Q (Fig. 6b-d)."
>
> "Strikingly, levels of angiomotin (AMOT), a member of the motin family linked to cell motility, angiogenesis, and regulation of YAP1-dependent Hippo signalling[39], were significantly reduced in 293T cells expressing $TBP_{1\text{-}106}$ 38Q, $TBP_{1\text{-}106}$ 106Q, or $TBP_{107\text{-}339}$ compared to cells expressing full-length TBP 38Q (Fig. 6e, f). The most pronounced reductions were observed in cells expressing the N-terminal fragments $TBP_{1\text{-}106}$ 38Q and 106Q."

Regarding the apparent discrepancy between calpain activation and AMOT reduction, we agree that AMOT dysregulation cannot be attributed solely to calpain overactivity. Our data support a multifactorial model involving (i) transcriptional dysregulation, (ii) co-aggregation-dependent protein sequestration, and (iii) calpain-mediated proteolytic cleavage. We explain this in the revised manuscript (please, see sixth Results section).

Within this framework, calpain overactivation represents one of several contributing factors as we reveal that transcriptional impairment – arising from polyQ expansion or loss of the C-terminal TBP domain – is the predominant driver of AMOT reduction (Fig. 6g, 7b, and new Fig. 7c), while co-aggregation (Supplementary Fig. 9a, b, e, f) and calpain-dependent cleavage provide additional, secondary contributions. This model explains why AMOT reduction is observed even in conditions without marked calpain activation and why the strongest decrease occurs in $TBP_{1–106}$ 106Q-expressing cells, where all three mechanisms converge.

To reflect this interpretation, we have added the following summary statement to the Discussion:

> "AMOT reduction appears to be multifactorial, resulting from transcriptional dysregulation, co-aggregation, and calpain-dependent fragmentation."

2. In Figure 6H, the authors used SCA17 patient fibroblasts to validate AMOT proteomic findings. It would be interesting to confirm whether levels of calpain 1, calpastatin or cleaved spectrin from SCA17 patient fibroblasts aligned with findings shown in Figure 6C. If the authors have previously examined calpain-activity in SCA17 fibroblasts, they should direct the reader to their previous work within the text, otherwise they should add these additional experiments within this study. Further, it is unknown whether the c-terminal or n-terminal TBP fragments (~30-20kDa, described in Figures 1 and 2) are present in SCA17 patient fibroblasts, and whether the T106 and S107 TBP cleavage site was validated in SCA17 patient fibroblasts. If TBP cleavage fragments of a similar size are observed in SCA17 patient fibroblasts, this should be reported and the authors should comment on the clinical relevance of the findings in Figures 1 and 2 to strengthen their findings. If these cleavage fragments were not observed in SCA17 fibroblasts, the authors should provide a possible explanation for their absence.

**Response:** We agree with the reviewer that the initial data from SCA17 patient-derived fibroblasts were not sufficient to fully support the findings obtained in 293T cells and SCA17 rat cerebellum. To strengthen the physiological relevance of our study, and in response to the other reviewers' suggestions, we established an additional patient-derived model system.

In collaboration with our colleagues at the German Center for Neurodegenerative Diseases (DZNE), we generated induced pluripotent stem cells (iPSCs) from SCA17 patient fibroblasts and differentiated them into induced cortical neurons (iCNs). In this additional model we assessed TBP expression, and detected increased baseline cleavage of polyQ-expanded TBP, elevated calpain activity, and the calpain dependency of TBP processing. Importantly, we confirmed the presence of TBP cleavage fragments comparable in size to those observed in 293T cells and SCA17 rat tissue.

The corresponding data, which strongly support the initial findings, are presented in the new Figure 8 and Supplementary Figure 12e-g, and are described in the revised Results and Discussion sections.

3. In Figure 5 the authors describe a relationship between expression of short TBP fragments and decreased AMOT levels but in Figure 6, decreased AMOT levels are observed in SCA17 patient fibroblasts without any comment on the possible associated with TBP fragmentation. Abundance of TBP cleavage fragments need to be quantified in the SCA17 patient fibroblasts. If these fragments are not present and therefore AMOT is not recruited to the short TBP fragment inclusion bodies, what is the mechanism for reduced AMOT levels in these cells? Further, the total amount of TBP (full length and cleavage together) should be quantified in these samples, as it appears to be lower than in the control individual fibroblasts.

**Response:** As outlined in our response to Point 2, we extended our analyses to SCA17 patient-derived induced cortical neurons and therefore discontinued additional analyses of fibroblasts to further investigate the relationship between polyQ-expanded TBP and AMOT dysregulation in a physiologically relevant context.

In this model, we confirmed a reduction in AMOT levels in SCA17 neurons compared to neurons derived from unaffected controls. Consistent with our previous observations, these cells also exhibit increased baseline calpain activity. In contrast to fibroblasts, we did not observe differences in total TBP levels between control and patient-derived neurons.

Notably, we did not detect TBP aggregates by microscopy in the neuronal model. However, the absence of detectable aggregates in induced neurons derived from patients with polyQ disorders has been widely reported and is therefore not unexpected. This suggests that AMOT reduction in this context is likely driven primarily by the observed calpain overactivation and/or polyQ-expanded TBP-related transcriptional dysregulation, rather than sequestration into inclusion bodies.

These additional data are presented in the new Figure 8 and Supplementary Figure 12e-g, and are described in the corresponding sections of the Results and Discussion.

4. In cases where amount of protein cleavage such as calpastatin cleavage is being examined, the cleavage fragment, not just the full length band, should be shown and quantified.

**Response:** We appreciate the Reviewer's suggestion and agree that inclusion of cleavage fragments, in addition to full-length proteins, can strengthen the assessment of calpain-mediated proteolysis. Accordingly, we routinely analyse multiple established calpain substrates to monitor proteolytic activity, including α-spectrin, calpain-1, and calpastatin (CAST), and, where appropriate, additional substrates depending on the cell type or tissue context.

However, the detection and quantification of cleavage fragments depend on several factors, including antibody sensitivity and epitope position as well as fragment stability. Based on our extensive experience with calpain substrates, α-spectrin – as well as βIII-tubulin and p35 in neuronal systems – provides robust and reproducible cleavage patterns with stable fragments

(Weber et al., 2017, *Brain*; PMID: 28334907; Weber et al., 2022, *CMLS*: PMID: 35482253). In contrast, CAST fragmentation, although detectable, is less suitable as a quantitative readout under basal or moderately induced conditions. CAST cleavage fragments are typically low in abundance and prone to rapid secondary degradation, resulting in increased variability.

To illustrate this, we provide representative data showing CAST cleavage upon CAPN1 overexpression, where fragmentation is sufficiently induced to be detected but remains variable across the three major fragment species (C1–C3) (Reviewer Figure 1). The observed variability likely reflects sequential degradation processes from C1 to C3. In addition, CAST expression itself is not significantly altered across our experimental conditions (Reviewer Figure 2), supporting the interpretation that observed changes primarily reflect proteolytic processing rather than altered expression levels.

Furthermore, many of our western blot analyses were optimized for the detection of full-length CAST and other proteins, often involving membrane segmentation (e.g., between ~75 kDa and ~100 kDa regions), which precluded reliable detection of smaller CAST fragments. As a result, retrospective quantification of CAST cleavage is not feasible for most datasets, and additional experiments would require new protein samples, which are no longer available in sufficient quantities.

Nevertheless, wherever technically feasible and biologically informative, we have included fragment-based analyses – for example, quantification of AMOT cleavage in Supplementary Figure 8b.

In summary, we are confident that our multi-marker approach, combined with both qualitative and quantitative analyses across independent experimental systems, provides robust evidence for calpain activity and its role in the mechanisms described in this study.

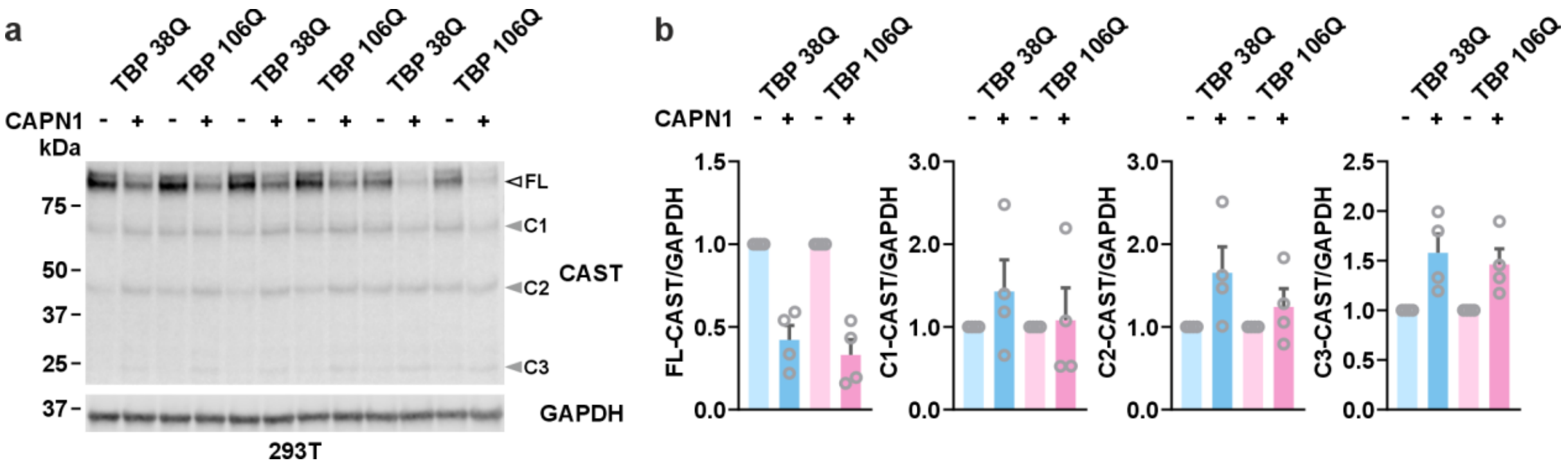


**Reviewer Figure 1: Calpain-1-dependent CAST cleavage in 293T cells expressing TBP 38Q or 106Q. a,** Western blot analysis of CAST in 293T cells transfected with myc-TBP 38Q or 106Q along with calpain-1 or an empty vector. CAST was detected with a specific antibody. GAPDH served as a loading control. **b,** Full-length (FL) CAST and CAST fragments C1, C2, and C3 were quantified and normalised to GAPDH. *n* = 4. Bars show means + s.e.m.

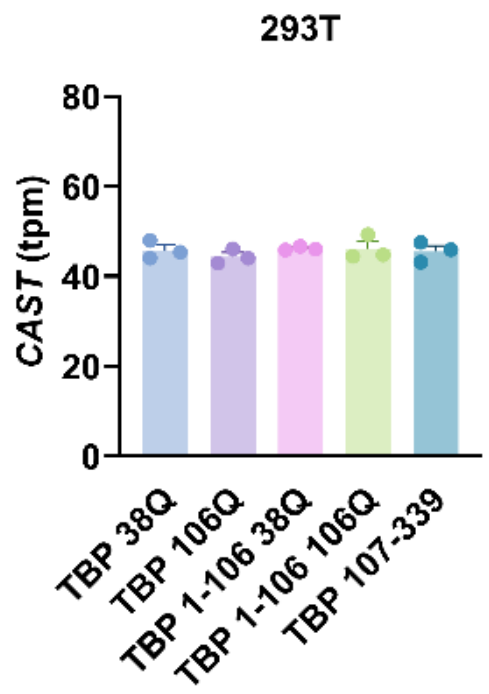


**Reviewer Figure 2: CAST expression in 293T cells transfected with different myc-TBP constructs.** Expression is shown as transcripts per million (tpm). Bars show means + s.e.m.

5. The authors performed AMOT knockdown experiments in two cell lines; HEK293T (consistent with other data from this paper) and SHSY5Y cells as a 'neuronal model.' A more useful neuronal model for investigation would be neurons reprogrammed from the SCA17 patient fibroblasts.
Further, the reduction in AMOT levels in SHSY5Y cells (Figure 7G) is relatively modest compared to the knockdown effect in HEK293T cells (Figure 7F). Do the authors have any hypotheses as to why the knockdown seems to have been less effective in their 'neuronal' cell line?

**Response:** We thank the reviewer for highlighting this important point. We agree that patient-derived model systems are, in principle, more physiologically relevant, although they also present technical limitations, such as reduced transfectability, which we encountered in previous studies. To validate key findings obtained in overexpression-based cell models, we have now included experiments using SCA17 patient-derived neurons, generated in collaboration with our partners at the DZNE in Tübingen, as mentioned in our responses to Points 2 and 3 (see new Figure 8 and new Supplementary Figure 8e-g).

Regarding the differences in knockdown efficiency, we routinely use both cell models, 293T and SH-SY5Y cells, in our laboratory, allowing us to directly compare their performance. SH-SY5Y cells generally exhibit lower growth rates and metabolic activity and – most relevant to the reviewer's question – a reduced capacity for DNA or RNA uptake during transfection compared to cell lines such as 293T or HeLa. Consequently, this leads to lower expression levels of plasmid-encoded proteins and reduced knockdown efficiency of target genes, as observed for *AMOT*. Even with optimized transfection conditions that preserve cell viability, the efficiency achieved in 293T cells cannot be matched in SH-SY5Y cells.

6. The size of the "TBP inclusions" shown within Figure 5 are very large. Additional imaging, including z-stack projections, are required.

**Response:** We thank the reviewer for this important suggestion. Indeed, we have consistently observed large inclusion bodies in overexpression-based cell models, not only for TBP but also for other polyQ-expanded proteins such as ataxin-1, ataxin-3, and huntingtin. In particular, fragment constructs of these aggregation-prone proteins often exhibit an increased propensity to form large inclusions.

To further support our findings for TBP, we reanalysed TBP inclusions using confocal microscopy and now provide additional overview images as well as z-stack projections in the new Figure 5b and Supplementary Figure 5c. These data further illustrate the presence, frequency, three-dimensional structure, and subcellular localization of TBP inclusions in our overexpression model.

In addition, to assess aggregation independently of the cellular context, we included data from our newly established membrane-based inclusion body detection assay using epifluorescence microscopy. These analyses confirm the presence of SDS-insoluble aggregates and highlight differences in aggregation behaviour between full-length TBP 106Q and the $TBP_{1–106}$ 106Q fragment. The corresponding data are presented in Supplementary Figure 9c-f.

7. On page 10, the authors conclude: "Additionally, we explored whether AMOT levels declined due to its recruitment into IBs formed by polyQ-expanded TBP or N-terminal TBP fragments. Epi-fluorescence microscopy revealed that AMOT colocalised with IBs formed by N-terminal TBP fragments (Supplementary Fig. 4d)." Does AMOT form inclusions that were not TBP-positive, i.e is AMOT alone able to form inclusions or are 100% of these structures co-localised with TBP inclusions? Secondly, do the authors know if full-length or cleaved AMOT is being recruited to the inclusion bodies? If there are AMOT inclusion bodies that are not TBP positive, where are these inclusions located (nuclear or cytoplasmic localisation?)

**Response:** We thank the reviewer for raising this interesting point. Using immunofluorescence analysis, we indeed observed cytoplasmic accumulations of AMOT (see Supplementary Figures 9a, g and 10c), which occurred independently of TBP or ataxin-3 overexpression and were not restricted to specific TBP species. However, these structures did not resemble the characteristic aggregates formed by polyglutamine-expanded proteins and were not positive for aggregation-associated markers such as polyubiquitination (Supplementary Figure 9g). We therefore interpret these structures as potentially representing functional assemblies or soluble reservoirs of AMOT, such as membrane-less organelles or biomolecular condensates, as described for other proteins and intracellular components (PMIDs: 28935776, 28225081).

Consistent with this interpretation, analysis using our membrane-based aggregation assay demonstrated that these AMOT-positive structures are SDS-soluble, in contrast to the SDS-insoluble aggregates formed by polyQ-expanded TBP (Supplementary Figure 9e, f). In the same assay, we also confirmed co-localization of AMOT within TBP aggregates. These data further support our initial observation that many, but not all, N-terminal TBP fragment-derived inclusion bodies are AMOT-positive. Conversely, AMOT-positive structures can occur independently of TBP aggregates and are predominantly cytoplasmic.

Regarding the nature of AMOT species recruited to inclusion bodies, co-aggregation was detected using a C-terminally binding AMOT antibody, suggesting that at least a fraction of AMOT is present in its full-length form within these structures. However, we cannot exclude the additional presence of cleaved AMOT species, and further studies will be required to resolve the precise composition and aggregation behaviour of AMOT in this context.

8. In the discussion (page 14, line 405) the authors state "The presence of an N-terminal polyQ stretch in AMOT may underlie its co-aggregation into TBP-positive IBs." The authors have not previously stated that AMOT contains a polyQ stretch. This concept seems highly relevant to the key story of the manuscript and should be addressed earlier. Further, the relevance of the polyQ stretch within AMOT should also be mentioned earlier when AMOT is described as a calpain substrate.

**Response:** We thank the Reviewer for these important suggestions. We have expanded two appropriate sentences in our Results section to feature the information on the presence of the polyQ stretch in the p130 isoform, which now read as follows:

> “To confirm this reduction, we analysed AMOT – present as a polyQ-stretch-containing p130 (UniProt ID: Q4VCS5-1) and a p80 (UniProt ID: Q4VCS5-2) isoform – by western blotting of protein extracts from 293T cells expressing full-length TBP or TBP fragments as well as mock controls transfected with an empty vector to assess the effect of TBP 38Q on AMOT baseline expression.”

> “Additionally, we explored whether AMOT levels declined due to recruitment into IBs formed by polyQ-expanded TBP or N-terminal TBP fragments, potentially driven by the polyQ stretch at the N-terminus of the p130 isoform.”

9. In Figure 4C, the authors display a western blot demonstrating changes in K48 polyubiquitin chains, p62 or LC3 but there is no quantification to accompany the western blot shown in Figure 4C.

**Response:** We now provide the quantification of the western blot detections shown in Figure 4c for all three proteins, which can now be found in the new Supplementary Figure 5a.

10. In Figure 8b, the authors describe differences in AMOT expression in zebrafish expressing TBP 38Q and 106Q. However, an older age, such as 27hpf or 72hpf should be examined so that the findings are not purely early developmental.

**Response:** We have expanded this analysis to older embryos and show now that significant differences in angiomotin expression persist also at a later stage. The data has been included in the Supplementary Figure 17b, c.

11. It needs to be stated that AMOT knockout in the CRISPR/Cas9 zebrafish is mosaic as F0 embryos are being examined. This examination of the injected animals will have potential effects of off target effects of the injection. Whilst control animals have also received cas9 injection, they have not received any guide RNA so the off target and injection related effects may be less. Examination of offspring from future generations would be more informative.

**Response:** In order to comply with 3R regulations and to reduce the number of animals used in experiments, we refrained from generating stable zebrafish lines and from analysing larvae older than 4 days post fertilization. Therefore, all experiments were done in embryos and transiently. As crispants can have the limitation of mosaic editing, we genotyped all embryos undergoing motoneuron analysis and considered only those showing an edited band in the

PCR as *amot* LOF embryos. In order to be transparent about this, we have added explaining sentences to the Results and Methods section.

> “As this may result in mosaic gene editing and can hence increase the variability of the phenotype, we genotyped each embryo.”

> “Gene editing in every individual embryo was verified by PCR amplification of exon 2 before further analysis. In addition, the editing capacity of the gRNAs was assessed and by Sanger sequencing (Microsynth AG, Göttingen, Germany) of exon 2 PCRs in edited and control-injected embryos.”

As requested by the reviewer, we furthermore assessed embryos injected not only with Cas9 protein, but also with ribonucleoprotein complex consisting of Cas9 protein and a non-editing control gRNA (Review Figure 3). Such embryos were indistinguishable to “Cas9-only” embryos so that we are confident that our initial conclusions presented in the manuscript are valid.

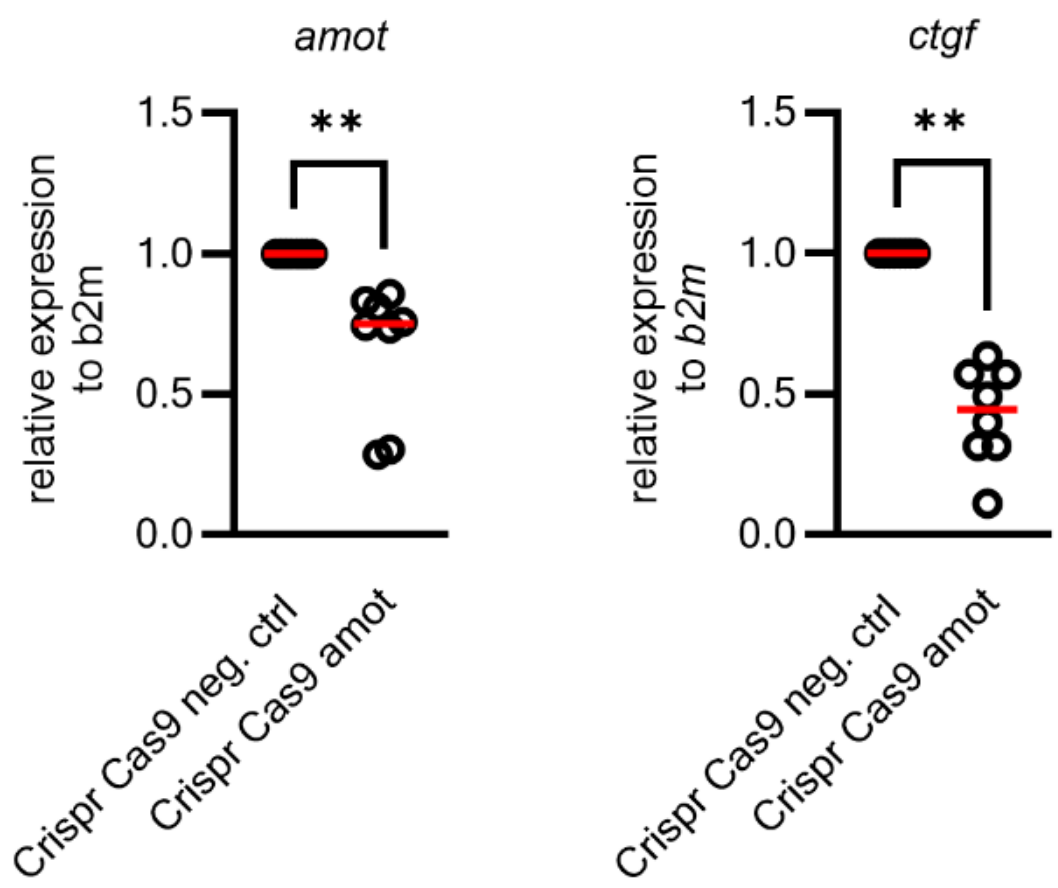


**Reviewer Figure 3: Control experiment for Crispr-based gene editing of amot in zebrafish embryos.** qPCR analysis of *amot* and *ctgf* expression in zebrafish embryos injected with either with Cas9 protein and a non-editing control gRNA as control (Crispr Cas9 neg. ctrl) or Cas9 along with *amot*-specific gRNAs (Crispr Cas 9 amot). $n$ = 8 experiments. Two-tailed Wilcoxon test with $^{**}p = 0.078$.

12. To obtain insights relating to the neuronal numbers, dopamine neuron numbers and tail coiling, the developmental stage of the animals examined needs to be confirmed and controlled for, such as through body length measurement.

**Response:** We did not see developmental delays upon *amot* loss-of-function or gross morphological changes which is consistent with previous reports (PMIDs: 17699752, 19590046). However, we took the concerns of Reviewer #2 very seriously. In the revised manuscript, we included additional images and phenotype analysis showing that embryos have the same length and an indistinguishable appearance as control embryos. The data is shown in the Supplementary Figure 17e-g, and referenced to in the text.

13. The various zebrafish phenotypes should be rescued through coinjection of human AMOT mRNA to prove that the reported phenotypes are AMOT dependent rather than off target effects.

**Response:** As suggested by the reviewer, we included rescue data for several of the observed phenotypes in the revised manuscript. This new data is included as in Figure 10f-i, and k and Supplementary Figure 17h.

14. TBP and spectrin protein cleavage should be examined in the AMOT knockout zebrafish, to demonstrate the altered processing in that model.

**Response:** We thank the reviewer for this valuable suggestion. We attempted to assess TBP and spectrin cleavage in the AMOT knockout zebrafish model by western blot analysis. However, despite multiple independent experiments, we were unable to obtain reproducible and robust data suitable for reliable interpretation.
This limitation is likely attributable to a combination of factors, including suboptimal detection by antibodies raised against antigens of mammalian origin. In light of these technical constraints, we concluded that further analysis would not yield sufficiently reliable results within the scope of this study and therefore did not include these data in the revised manuscript.

15. The zebrafish findings put forward decreased motor neurons in the AMOT knockout zebrafish, but increased dopamine neuron numbers, increased aberrant motor neuron axons, increased spontaneous coiling, but decreased touch evoked responses. This combinations of phenotypes needs describing and discussion.

**Response:** For more clarity, we have added the following sentences in the Results section:

> "As a measure of ataxia, we performed simple behavioural tests routinely used to assess mechanosensory and motoric functions in zebrafish embryos[48, 49]. Depletion of *amot* by Crispr/Cas9 manifested in more spontaneous movements in 24 hours post fertilization (hpf) zebrafish embryos as well as loss of touch response and touch-evoked swimming at 48 hpf, suggesting general motor impairments, which were rescued by overexpression of human AMOT (Fig. 10f-h, Supplementary Fig. 17h)."

16. Line 416 states that "To investigate the neuronal role of AMOT in vivo, we genetically depleted amot in zebrafish embryos. This resulted in disrupted YAP1-associated Hippo signalling, impaired motor neuron development, and reduced motor function." This is an overstatement. Disrupted YAP1-associated Hippo signalling is not demonstrated in the AMOT knockout zebrafish, two transcripts are shown to be present at altered abundance, but signalling disruption is not demonstrated. "reduced motor function" is mentioned, but increased spontaneous coiling is presented, so that statement needs rewording.

**Response:** We changed the sentence as suggested to avoid an overstatement:

“This resulted in downregulation of YAP1 target genes, impaired motor neuron development, and altered motor function.”

17. On page 8, lines 205-207 the authors state: "Conversely, the C206 terminal fragment TBP107-339, which contains the DNA-binding core domain, was primarily found in the nuclear fraction (Fig. 5g)." The authors should consider altering the working of this statement, as the inference from this statement is that expression is very low within the cytoplasm however the western blot image shown in Figure 5G shows the c-terminal fragment band is clearly visible within the cytoplasmic fraction, albeit at a relatively lower density. Perhaps a more suitable description would that this c-terminal fragment is found in both the cytoplasm and nucleus, however expression of the c-terminal fragment appears to be stronger in the nuclear fraction.

**Response:** We thank the reviewer for raising this relevant point and agree that a less absolute description of $TBP_{107–339}$ localization is more appropriate. Our initial interpretation was primarily based on immunofluorescence analyses, which show a predominantly nuclear localization of the C-terminal TBP fragment, comparable to full-length TBP 38Q (Figure 5a).

We acknowledge that the fractionation assay presents a more nuanced picture, with a detectable signal also present in the cytoplasmic fraction, as correctly noted by the reviewer, and confirmed by us when reanalyzing the fractionation using myc tag-specific antibody (new Fig. 5i-j). Given the known limitations of fractionation approaches with respect to compartmental purity, we consider the microscopy-based assessment to be more representative of the overall localization pattern. To better reflect the combined evidence, we have revised the corresponding statement in the manuscript as follows:

> “Conversely, the C-terminal fragment $TBP_{107-339}$, which contains the DNA-binding core domain, is enriched in the nuclear fraction, consistent with microscopy data, while lower levels remain detectable in the cytoplasmic fraction (Fig. 5i, j, Supplementary Fig. 5b). In summary, whereas C-terminal TBP predominantly localises to the nucleus, N-terminal polyQ-expanded TBP fragments mislocalise to the cytoplasm, where their aggregation-prone nature promotes the formation of perinuclear IBs.”

**Minor points:**

1. AMOT knockout in the zebrafish is shown in Supplementary Figure, this should be shown in Figure 6 instead, it is required within this study.

**Response:** We appreciate that the Reviewer values this data. The data panel has been moved to an appropriate position and can now be found in Figure 10b.

2. The schematic in Figure 3C shows 9 indicators of CAPN1 or chymotrypsin cleavage and 13 different amino acid sequences produced by this cleavage. The in silico results need further explanation to clearly explaining how the 10 peptides were identified, with further information including 1) how many cleavage sites were identified in total using the GPS-CCD tool, 2) which proteases were predicted to cleave at these sites, 3) how many sites were predicted to be calpain cleavage sites and 4) how did this compare to your in vitro calpain 1 data.

**Response:** We thank the reviewer for this important point and are happy to clarify our approach. Figure 3c indicates in total 13 calpain-1-derived cleavage sites (yellow dashed lines) and 5 chymotrypsin cleavage sites (pink dashed lines) (We have added this numerical information in the corresponding figure legend). The GPS-CCD tool (PMID: 21533053) is an established prediction algorithm that identifies calpain cleavage sites based on a dataset of 368 experimentally validated sites across 130 proteins, independent of specific calpain isozymes. Consistent with this, our previous in vitro studies on ataxin-3 and TBP (PMID: 28334907; PMID: 35482253) demonstrated that the two best-characterized calpains, calpain-1 and calpain-2, generate highly similar cleavage patterns. Thus, predicted sites are considered calpain-specific but are not restricted to a single isozyme, in line with the current understanding that calpains do not follow strict isozyme-specific sequence motifs.

For cleavage site prediction, we applied the highest confidence threshold (normalized to 1), with values above this cutoff considered indicative of potential cleavage sites (Figure 3d). After excluding a polyQ stretch–related prediction artefact, a total of 30 potential sites were identified across the TBP sequence.

Mass spectrometry analysis detected 13 TBP-derived peptides with at least one terminus not attributable to chymotrypsin cleavage (which occurs after F, Y, or W). Peptides consistent with chymotryptic cleavage at both termini were not observed. Comparison of these non-chymotryptic termini with predicted calpain cleavage sites revealed that all 13 peptides aligned with predicted sites. Three peptides were located within the C-terminal tag and were therefore excluded as non-physiological. The remaining 10 peptides were thus considered to originate from calpain-mediated cleavage. Notably, one peptide was consistently detected across all experimental replicates, corresponding to cleavage between T106 and S107, which we therefore define as a prominent calpain cleavage site.

To improve clarity, we have revised the corresponding sentences in the Results section as follows:

> "Subsequent MS analysis identified 13 distinct peptides derived from myc-tagged TBP, each containing one terminus not attributable to chymotryptic cleavage (Supplementary Table 1). Comparison of these termini with *in silico* calpain cleavage site predictions for TBP (UniProt ID: P20226) using the GPS-CCD tool revealed that 10 peptides corresponded to predicted calpain cleavage sites within the TBP sequence, whereas three mapped to the C-terminal tag and were excluded (Fig. 3c, d; Supplementary Table 1)."

3. In Figure 5, the authors observed structural differences in inclusion body formation following expression of full-length TBP 106Q compared with TBP fragments 1-10638Q and 1-106106Q. In Supplementary Figure 4, it is shown that AMOT is recruited to the IBs produced by shorter TBP fragments and that this could explain the differences in localisation (nuclear vs perinuclear) and increased IB size. Another factor that could contribute, could be the presence or absence of NES or NLS sequences. Do the authors know whether there are NES or NLS signals within TBP and how the presence or absence of these signals could affect fragment localisation?

**Response:** Based on the current state of knowledge, TBP does not contain classical NES or NLS sequences. In yeast, nuclear import of TBP has been described to depend on a non-classical binding by karyopherin Kpn114p, the yeast homolog of human importin-9. The relevant studies have been cited in our manuscript (Pemberton et al. 1999; Morehouse et al. 1999; Liao et al. 2020).

The interaction between Kpn114p and yeast TBP has been characterized in detail (Liao et al., 2020, PMID: 32484313; Liao et al., 2023, PMID: 37684250), demonstrating that binding occurs near the N-terminus of the C-terminal core domain of yeast TBP (amino acids 60-240), corresponding to amino acids 158-339 of human TBP. These findings may explain why, in our experiments, the $TBP_{107-339}$ localises to the nucleus, whereas N-terminal $TBP_{1-106}$ fragments, which lack the interaction region, remain cytoplasmic.

This interpretation is reflected in the following sentence within our Discussion:

> "However, the current observation is consistent with predicted C-terminal nuclear localisation signals[15] and previously reported karyopherin-mediated nuclear shuttling in yeast, which depends on a recognition motif located near the protein's C-terminal core domain[54, 55]."

4. The authors state on line 149, page 7 "... a primary calpain-1 cleavage event takes place between T106 and S107." To provide further conclusive experimental evidence, the authors could perform a site directed mutagenesis and demonstrate absence of calpain cleavage following mutation of the calpain cleavage site.

**Response:** We agree with the reviewer that additional experimental validation of the identified cleavage site is important to support its functional relevance. Although such validation is technically challenging, we addressed this point using a mutagenesis strategy previously established in our studies on ataxin-3 (PMID: 28334907).

Specifically, we mutated amino acids 105–108 surrounding the predicted cleavage site to tryptophan (W) residues. This approach resulted in a marked reduction of TBP cleavage at the T106|S107 site, as evidenced by decreased generation of the N1 and C1 fragments in both wild-type and polyQ-expanded TBP. As anticipated, this mutation also led to an increased production of alternative, lower-molecular-weight C-terminal fragments, likely reflecting the utilization of adjacent downstream calpain cleavage sites.

The mutagenesis strategy, together with the corresponding experimental results, is presented in the new Figure 3f-h and Supplementary Figure 4.

5. In Figure 8J, the authors describe the effects of Amot knockdown on dopaminergic neurons. Why were dopaminergic neurons examined? What is the link between dopaminergic neurons and Amot? This rationale should be more clearly explained.

**Response:** We have added a sentence in the Results section and modified this section in the Discussion:

> "As in the context of HD, a related condition, changes in the dopaminergic system have been reported[50-52], we additionally examined the zebrafish brain. Here, loss of Amot impacted the dopaminergic system by increasing the number of dopaminergic neurons, …"

> "Very little is known about early cellular or molecular changes in the development of SCA17. When the disease has progressed, however, a reduction in dopamine transporter expression or activity has been reported[71]. Our study shows that *amot* depletion increases dopamine transporter levels and produces a higher number of dopaminergic neurons – features also described in the context of HD[50-52]. Importantly, overexpression of the dopamine transporter has been shown to eventually cause dopaminergic neuron loss[72]. Thus, we speculate that early upregulation of the dopaminergic system may precede the loss of dopaminergic activity observed at later stages."

6. The findings presented in Supplementary Figures 4 and 5 are interesting. One interpretation is that polyQ containing proteins (either full-length or shorter fragments) recruit AMOT to inclusion bodies, leading to decreased soluble levels of AMOT. Have the authors examined whether AMOT colocalises with TBP107-339 which does not contain a polyQ stretch?

**Response:** Our immunofluorescence-based microscopy (Figure 5a), fractionation assays, (Figure 5i, j; Supplementary Figure 5b) and filter retardation assay analyses (Figure 4g) demonstrate that the $TBP_{107–339}$ fragment localizes primarily to the nucleus and does not form aggregates. Consistent with these properties, we did not observe colocalization with the cytoplasmically localized AMOT protein via microscopy or evidence of co-aggregation (see revised Supplementary Figure 9a).

7. In the discussion, the authors mention that non-expanded TBP promotes AMOT levels. This point should be clarified as the current manuscript does not provide experimental evidence as to whether TBP 38Q increases or promotes AMOT level, i.e. does overexpression of TBP 38Q increase AMOT levels above endogenous AMOT levels?

**Response:** Our statement in the Discussion is based on western blot analyses of AMOT in 293T cells overexpressing TBP compared to empty vector/mock-transfected controls (Figure 6g, Supplementary Figure 6c), as well as on zebrafish experiments using *amot*-specific in situ hybridization (ISH) (Figure 10a; Supplementary Figure 17b, c). Both approaches demonstrate a significant increase in endogenous AMOT/*amot* levels in the presence of non-polyQ-expanded (wild-type) TBP, with the cell-based analysis showing an approximately twofold increase relative to mock controls, while polyQ-expanded TBP fails to increase AMOT expression to the same degree.

To further strengthen this experimental data, we performed qPCR analysis of 293T cells overexpressing TBP 38Q or TBP 106, compared to cells transfected with an empty vector. Our

analysis reproduced the effects detected on protein level in 293T cells and by ISH in zebrafish embryos, and is shown in Figure 7c and described as follows in the Results section:

> "To further evaluate the TBP-dependent modulation of AMOT expression, we performed qPCR analysis in 293T cells transfected with TBP 38Q, TBP 106Q, or an empty vector. Importantly, and corresponding to our western blot data, TBP 38Q increased expression of AMOT, while TBP 106Q showed a deficiency in *AMOT* upregulation (Fig. 7c)."

To further highlight the basis of this statement for the reader, we have expanded the respective sentences in the Discussion as follows:

> "Among the affected factors, we identified AMOT as significantly downregulated in cells expressing polyQ-expanded TBP or its fragments, whereas non-polyQ-expanded TBP appeared to promote *AMOT* expression, as demonstrated in TBP-overexpressing 293T cells compared to mock-transfected controls at both the mRNA and protein level. […] In SCA17 rats, the presence of polyQ-expanded TBP similarly led to reduced AMOT levels, particularly in the molecular layer of the cerebellum. A comparable regulatory pattern was observed in zebrafish: overexpression of non-polyQ-expanded TBP increased *amot* mRNA levels, whereas polyQ-expanded TBP failed to promote *amot* expression, suggesting an evolutionarily conserved mechanism."

8. No quantification of the data presented in Supplementary Figure 4A. Quantification of these westerns is needed to support the other findings within this figure.

**Response:** At the Reviewer's request, we have provided quantification of markers shown in Supplementary Figure 3a (now Supplementary Figure 8b), including levels of full-length and calpain-cleaved AMOT p130. In addition, we demonstrate the inhibitory effects of two further calpain-specific inhibitors, calpeptin (CLP) and calpain inhibitor III (CI-III), by western blot, and provide quantitative analysis of the inhibitory effect of CI-III on calpain-mediated AMOT p130 cleavage. Corresponding revisions have been made in the Results section.

9. The conclusion from Supplementary Figure 4 is that AMOT is a calpain substrate. To support the findings in Supplementary Figure 4, the authors should discuss whether the protein sequence of AMOT contains any calpain cleavage motifs.

**Response:** We thank the Reviewer for this helpful suggestion. We have now included a calpain cleavage site prediction for AMOT using the GPS-CCD tool, presented in the new Supplementary Figure 9a. The *in-silico* analysis identifies several sites along the AMOT sequence with high predicted cleavage likelihood, including two prominent sites at the N-terminus and a broader region containing multiple potential cleavage sites within the ~200 most C-terminal amino acids. This cluster of predicted sites may account for the fragments detected in Supplementary Figure 9b. A reference to these findings has been added to the Result section.

10. In Supplementary Figure 3 and Figure 7, naming of experimental conditions alternates between TBP1-106 38Q or 38QF, TBP1-106 106Q or 106QF, and TBP107-339 or CF. For clarity, I would suggest keeping the naming of experimental conditions consistent with the bulk of the figures, i.e. TBP1-106 38Q, TBP1-106 106Q and TBP107-339.

**Response:** We agree with the Reviewer that a consistent labelling of experimental conditions is more comprehensible for the reader. We followed the Reviewer's suggestion and revised Figure 6 and 7, as well as Supplementary Figures 6 (former Supplementary Figure 3) and 11 accordingly.

**Reviewer #3 (Remarks to the Author):**

The manuscript “Calpain-mediated TBP cleavage disturbs neuronal AMOT-YAP1 2 signaling” is an interesting paper that shows that transcription factor TBP is cleaved by calpains, the site of cleavage is after residue T106, and the N-terminal fragment localizes to the cytoplasm. TBP is an essential transcription factor, and polyglutamine expansion in this TF causes SCA17. Proteomics analysis links the TBP fragments to AMOT and further analysis shows nice correlation with transcriptional changes. The work is rigorous, and the figures are well-constructed and informative. A deeper analysis of the rat SCA17 model to validate their findings would strengthen the research.

**Response:** We thank the Reviewer for their positive assessment of our work and for their valuable suggestions to further improve its validity and relevance.

**Points to consider for revision are:**

Point 1. The abstract requires a few more specific details. For example, “Multiomics analysis of what? SCA17 cells, SCA17 mice and zebrafish…

**Response:** We thank the Reviewer for pointing out the lack of detail in the abstract. We have now added specific information on the models used, which now reads as follows:

> “SCA17 cell line-based multi-omics analyses revealed that TBP fragments alter pathways linked to neuronal function and neurodegeneration, identifying AMOT as a key player. Further analyses in SCA17 cell lines, patient-derived fibroblasts and induced neurons as well as in rat and zebrafish models confirmed AMOT’s pivotal role in neurons, regulated by TBP and mediated via YAP1-dependent signalling.”

Point 2. Remove the word “Beyond transcription, ….. This does not make sense as the transcriptional activity is what is crucial for neurological function.

**Response:** We agree that the phrase “Beyond transcription” is misleading and have removed it from the respective sentence.

Point 3. “Despite its discovery over two decades ago, the molecular pathomechanisms driving selective neuronal loss in SCA17 remain unclear”. I don’t think the author’s current paper addresses selective neuronal loss, so maybe remove the word selective.

**Response:** We concur that our study does not address selective neuronal loss or vulnerability and have therefore removed the term “selective” from the sentence.

Point 4. The references are not cited appropriately and should be the references to the first studies. Under the toxic fragment hypothesis, proteolytic cleavage of disease-linked proteins is a key event in neurodegeneration, as seen in AD, PD, and polyQ disorders such as Huntington’s disease (HD), and SCA3, the following key references.

**Response:** We thank the Reviewer for raising this important point. We have revised the reference list accordingly and now include key early studies describing proteolytic cleavage events in the exemplary neurodegenerative disorders mentioned. While this selection is not intended to be exhaustive, it ensures appropriate citation of primary work in the field.

Point 5. Figure 1. Please state in the legend the cell type the TBP was immunopurified. Also, in panel 1d, the second western blot from left, the three bands (n1, n2 and n3) are not clear in the 38Q IP. Perhaps a darker exposure.

**Response:** We thank the Reviewer for this comment. The legend to Figure 1 already specified the cell model ("Full protein extracts from TBP-transfected 293T cells (input) were loaded along the IP samples." For additional clarity, we have now also included the cell line in the opening sentence of panel b: "Western blot analysis of TBP fragmentation after immunoprecipitation (IP) from 293T cell extracts, followed by a calpain cleavage assay with calpain-1 (CCA)."
In addition, we have replaced the D5C9H detection in Figure 1d with a one of longer exposure to improve the visibility of fragments n1–3.

Point 6. The paper focus on calpain-1 which they state is predominantly localized to the cytoplasm. Other calpain family members have nuclear localization and some work on polyQ diseases addresses this. This is important because the model that the authors suggest is that TBP fragmentation by calpain-1 occurs in the cytoplasm, followed by the nuclear translocation of TBP fragments c1 and c2. It is likely that other calpain family members can cleave TBP in the nucleus and the model they generate is very artificial with overexpression. Calpain-1 overexpression will activate these enzymes.

**Response:** The Reviewer raises an important point. In our in vitro and cell-based assays, we primarily focused on calpain-1, which – together with calpain-2 – is among the best-characterized members of this protease family. While our data clearly demonstrate that calpain-1 can mediate TBP cleavage, we cannot exclude the possibility that other calpains, including those with potential nuclear localization, may also contribute.

We note that our initial submission already included ionomycin-based calpain activation assays with inhibitor controls, demonstrating TBP cleavage independent of calpain-1 overexpression in our cell model (Supplementary Figure 2a, b). In response to requests from Reviewers #1 and #3 for further validation of our hypothesis that TBP cleavage occurs prior in the cytoplasm prior to nuclear import, we performed additional experiments.

Previous studies have established that TBP is translated in the cytoplasm and subsequently translocated to the nucleus (Morehouse et al., 1999, PMID: 10535958; Pemberton et al., 1999, PMID: 1038552). Using our transcriptome data (*n* = 3), we identified calpains expressed at appreciable levels in our cell model, including CAPN1, *CAPN2*, CAPN7, and *CAPN15* (44, 86, 49, and 67 TPM, respectively), while *CAPN5* and *CAPN10* were expressed at lower levels (10 and 15 TPM), and *CAPN6* and *CAPN12* were minimally expressed (1 and 2 TPM; Supplementary Figure 2c).

We then assessed the subcellular localization of the relevant calpains, excluding negligibly expressed isoforms. Database analysis (e.g., The Human Protein Atlas) indicated that only calpain-5 and calpain-15 may exhibit partial nuclear localization. However, recent studies report predominantly cytoplasmic or membrane-associated localization for these proteases (Gal et al., 2022, PMID: 35643222; Noguchi et al., 2025, PMID: 41380969). To experimentally validate this, we performed immunocytochemistry and fluorescence microscopy for the expressed calpains, as well as for the small calpain subunit CAPNS1 and calpastatin (CAST). All proteins displayed predominantly cytoplasmic localization, although with varying degrees of subcellular compartmentalization (Supplementary Figure 2d). These findings support the cytoplasm as the primary site of calpain-mediated TBP cleavage.

To assess the contribution of endogenous calpains, we performed knockdown experiments targeting calpain-1, calpain-2, and CAST, and analysed TBP fragment patterns at baseline. *CAPN1* knockdown markedly reduced TBP fragment c1 levels, whereas CAPN2 knockdown had no detectable effect (Supplementary Figure 2e, f). In contrast, CAST knockdown increased TBP cleavage, as expected (Supplementary Figure 3).

These data have been incorporated into the revised manuscript, and the following statement has been added to the Discussion:

> "In the present study, we confirmed our initial findings and demonstrate that calpain-dependent TBP cleavage – given the predominantly cytoplasmic localisation of these proteases – is a non-nuclear process that affects cytoplasmic TBP after translation but prior to its reported nuclear import[30]."

While our results strongly support calpain-1 as the primary mediator of TBP cleavage in our cell models, we cannot exclude that other calpains may contribute under different cellular contexts or in specific tissues. To acknowledge this, we have added the following limitation to the Discussion:

> "However, further research is required to determine whether this mechanism is conserved across cell types and tissues."

Point 7. The conclusion for Figure 4 is reasonable "our findings indicate that N- and C-terminal TBP fragments derived from calpain cleavage at T106|S107 exhibit reduced protein levels. This reduction is attributed to high proteolytic turnover via the proteasomal pathway for the C-terminal fragment, while the N-terminal polyQ-containing fragments, particularly polyQ-expanded TBP1-106 106Q, demonstrate a high degree of aggregation".

**Response:** We thank the Reviewer for this positive assessment. To further improve clarity and conciseness, we have revised the concluding sentence of Figure 4 as follows:

> "In summary, TBP fragments generated by calpain cleavage at T106|S107 display reduced protein levels, driven by rapid proteasomal turnover of the C-terminal fragment and pronounced aggregation of N-terminal polyQ-containing fragments, particularly $TBP_{1–106}$ 106Q."

Point 8. It makes sense from the proteomics to evaluate AMOT but when it is introduced in the text it would be helpful to the reader to explain what it does functionally. Also, the authors do not do enrichment analysis of the proteome altered by the TBP1-106 106Q fragment, nor compare their results to more human-relevant models like fibroblasts or the proteome of the TBP polyQ expansion rat, which would strengthen its significance.

**Response:** We thank the reviewer for these helpful comments. First, we have included a brief, referenced description of the originally described function of AMOT at its first mention in the manuscript, which reads as follows:

> "Strikingly, levels of angiomotin (AMOT), a member of the motin family linked to cell motility, angiogenesis, and regulation of YAP1-dependent Hippo signalling[39], were significantly reduced in 293T cells expressing $TBP_{1-106}$ 38Q, $TBP_{1-106}$ 106Q, or $TBP_{107-339}$ compared to cells expressing full-length TBP 38Q (Fig. 6e, f)."

Second, as suggested, we reanalysed the dysregulated proteome associated with the $TBP_{1-106}$ 106Q fragment. Although the number of significantly altered proteins was too limited to permit robust pathway or gene enrichment analyses, the identified proteins showed a tendency to cluster in interconnected networks linked to AMOT (Supplementary Figure 6b). Notably, literature curation revealed that all identified proteins have previously been linked to neuronal pathways, supporting the relevance of this dataset in the context of neurodegenerative disease, despite the use of a general cell model such as 293T cells. These annotations and references are provided in the new Supplementary Table 3 and have been incorporated into the manuscript text.

In addition, we performed proteomic analyses of SCA17 rat cerebellum samples. While the detection of YAP1 signalling components and overlap with the 293T dataset was limited, likely reflecting technical constraints of the proteomics approach (Supplementary Figure 15c), we identified alternative dysregulated proteins and pathways that further support a link between YAP1 signalling and SCA17-associated neuropathological changes.

Further details are provided in our response to Point 9, as well as in the new data presented in the new Figure 9 and new Supplementary Figure 13, 14, 15, and 16.

Point 9. The analysis of the SCA17 rat model is rather limited for the paper. It would be reasonable to see the generation of the relevant calpain fragment in this model, an neoepitope antibody to the cleavage product, western blot and IHC of the key players in this paper for validation of the mechanism. Generally the paper focuses on mechanistic studies in 293T cells and this may not be relevant to SCA17 and needs further validation.

**Response:** We thank the reviewer for this valuable suggestion and have taken substantial steps to expand our dataset by incorporating additional model systems. Specifically, we included SCA17 patient-derived neurons (presented in the new Figure 8 and new Supplementary Figure 12) and, as suggested, extended the analysis of our SCA17 rat model beyond the previously used 293T and SH-SY5Y cells, patient-derived fibroblasts, and the zebrafish model.

In addition to western blot-based analysis of AMOT levels (Figure 9c) and the IP-facilitated first-time detection of N-terminal polyQ-expanded TBP fragments in SCA17 rat cerebellum (Supplementary Figure 13b-e), we performed histological analyses, which confirmed comparable effects on AMOT at the tissue level (Figure 9a, b).

We further expanded the analysis of YAP1 signalling by including additional downstream markers, particularly CCN1 and CCN2, and demonstrated that SCA17 rats exhibit reduced levels of myelin basic protein (MBP) (Figure 9d), consistent with recently reported CCN1-associated myelination deficits in the brain (PMID: 41407862).

However, we decided against generating neo-epitope–specific antibodies, as suggested by the reviewer, despite their potential value. This decision is based on our previous experience with neo-epitope antibody generation against calpain-derived ataxin-3 fragments, which we had described in an earlier study (PMID: 28334907). Despite years of immunization and optimization efforts, we have, to date, been unable to obtain suitable antibodies for calpain-cleaved ataxin-3.

As noted under point 8, we also performed proteomic analyses of the cerebellar tissue. In addition to pathway and gene enrichment signatures linked to neuronal dysfunction and neurodegeneration related to disease such as ALS, PD, and SCAs, these analyses revealed a significant overrepresentation of downregulated proteins associated with oligodendrocytes, the myelinating cells of the central nervous system.

Together, these data – presented in the new Figure 9 and new Supplementary Figures 13, 14, 15, and 16, and described in the corresponding Results and Discussion sections – provide strong in vivo support for our findings from cell-based models.

**Reviewer #4 (Remarks to the Author):**

I co-reviewed this manuscript with one of the reviewers who provided the listed reports. This is part of the Nature Communications initiative to facilitate training in peer review and to provide appropriate recognition for Early Career Researchers who co-review manuscripts.

**Response:** We highly appreciate the Reviewer's comments and suggestions, which were developed in collaboration with Reviewer #2, and we hope that our revisions have addressed them satisfactorily.

**RESPONSE TO REVIEWER COMMENTS**

Reviewer #1 (Remarks to the Author):

Review for NCOMMS-25-39340A
Calpain-mediated TBP cleavage disturbs neuronal AMOT-YAP1 signalling
This is a revised manuscript, I appreciate the authors' efforts to perform additional human cell experiment and added discussions to clarify the current study discrepancy with their previous work.
I have carefully read the revised manuscript and still have some concerns that need the author to address to enhance the manuscript's strength.

**Response:** We thank the reviewer for their careful evaluation of our revised manuscript and for acknowledging our efforts to include additional human cell experiments and to clarify discrepancies with our previous work. We appreciate your constructive feedback and the remaining concerns raised, which we believe will further strengthen the manuscript. We address each of these points in detail below.

Figure 8, This is a new figure showing that the SCA-17 iPSC also showed TBP fragmentation and dysregulated AMOT. While it is a plus using patient cells, the immunofluorescent staining of AMOT and the western blot of AMOT seem not correlate well. It looks like the western blot shows AMOT in SCA-17 iPSC is significantly decreased, however, the immunostaining of the cells does not look much different compared to the control iPSC, except the cell body may look smaller. The AMOT staining in Figure S12 also has the same problem. The authors might need to use a different antibody or use a different antibody dilution for the immunostaining of the cells. The control and SCA17 patient iPSC should indicate the polyQ number.

**Response**: We thank the reviewer for pointing out this discrepancy between the immunofluorescence and western blot data. The initially presented images were selected to illustrate the subcellular distribution of the proteins of interest (AMOT and TBP), rather than to reflect the observed reduction in AMOT levels in induced cortical neurons (iCNs) and fibroblasts.
To address this concern, we have performed additional immunofluorescence microscopy and now include representative images that better reflect the decrease in AMOT levels. These updated panels are shown in Figure 8a and Supplementary Figure 12b and c.

Minor:
Supplement S5 Legend does not have a description for figure "a ". Need to add it for less confusion.

**Response:** We thank the reviewer for pointing this out. While the corresponding panel description was present in the figure legend, the label "a" was inadvertently omitted during the revision process. We have now corrected this error.

The control and SCA17 patient iPSC should indicate the polyQ number.

**Response:** We thank the reviewer for this important comment. While the polyQ (CAG/CAA) repeat length of the SCA17 patient was unintentionally omitted, the repeat length of the unaffected control individual and the derived cell lines had not been determined previously.
To address this, we performed repeat length analysis for both the SCA17 and control lines. The SCA17 patient carries 37 CAG/CAA repeats on the wild-type allele and 52 repeats on the pathogenic expanded allele of *TBP*. In contrast, the control line shows homozygous repeat lengths of 37 CAG/CAA repeats on both alleles.
We have now included this information in the text of the manuscript, along with a description of the method and the corresponding electropherograms as Supplementary Figure 12a.

Reviewer #2 (Remarks to the Author):

The manuscript is greatly improved.

All provided feedback has been responded to.

Inclusion of induced cortical neurons in addition to the SCA17 patient fibroblast findings, and expansion of those findings to cover additional components that had been requested is a great improvement and appreciated.

**Response:** We thank the reviewer for the positive evaluation of our revised manuscript. We appreciate your recognition that the manuscript has improved and that your comments have been addressed.
We are particularly grateful for your acknowledgment of the inclusion of induced cortical neurons alongside the SCA17 patient fibroblast data, our additional validation experiments for our zebrafish models, and the expansion of our analyses.
We have addressed the remaining comments and suggestions as follows.

The authors denote the generated cells as cortical neurons, but the only validation provided is MAP2 staining. Because MAP2 is a pan-neuronal marker, it is insufficient to confirm regional identity, functional subtype, or culture purity. To scientifically support the claim that these are indeed cortical neurons, the manuscript would require inclusion of specific validation markers (e.g., PAX6, FOXG1, TBR1, and vGLUT1) via immunocytochemistry or RT-qPCR.

**Response:** We thank the reviewer for highlighting the need for additional validation to support the cortical identity of the generated neurons. We agree that MAP2 alone is insufficient to confirm regional specification, functional subtype, or culture purity.
In response, we have now included representative microscopy panels showing the cortical markers TBR1 and CTIP2, as well as the pan-neuronal marker βIII-tubulin, for both control and SCA17 lines. These results are presented in the updated Supplementary Figure 12f with the corresponding figure legend, and are also briefly referenced in the relevant Results section.

Additional requested rescue experiments and validation experiments within the zebrafish findings are well received (especially supp Figure 17e-g).

Some minor points:
*It looks like Supplementary Figure 12d (in figure legend) should be g.

**Response:** We thank the reviewer for their careful reading of the manuscript. We have corrected this error; the panel previously referred to as Supplementary Figure 12d is now correctly labelled as Supplementary Figure 12i following the inclusion of the new panels 12a and 12f.

*Within response 5) within rebuttal document a new Supplementary Figure 8e-f is described, but this cannot be found in the figures. It is likely Supp Figure 12e-g, which is fine.

**Response:** We also thank the reviewer for identifying the numbering inconsistency in the previous rebuttal document. We appreciate that the reviewer correctly associated our response with the relevant content in Supplementary Figure 12.

Reviewer #3 (Remarks to the Author):

I am satified with the careful responses to my review and the revised experiments and edits to the manuscript.

**Response:** We sincerely thank the reviewer for their positive assessment of our revisions. We greatly appreciate your acknowledgment of the careful responses, additional experiments, and improvements made to the manuscript. Your feedback has been invaluable in strengthening the quality and clarity of our work.
